# Can Serendipity Still Hold Any Surprises in the Coordination Chemistry of Mixed-Donor Macrocyclic ligands? The Case Study of Pyridine-Containing 12-Membered Macrocycles and Platinum Group Metal ions Pd^II^, Pt^II^, and Rh^III^

**DOI:** 10.3390/molecules26051286

**Published:** 2021-02-27

**Authors:** Alessandra Garau, Giacomo Picci, Massimiliano Arca, Alexander J. Blake, Claudia Caltagirone, Greta De Filippo, Francesco Demartin, Francesco Isaia, Vito Lippolis, Anna Pintus, M. Andrea Scorciapino, M. Carla Aragoni

**Affiliations:** 1 Dipartimento di Scienze Chimiche e Geologiche, Università degli Studi di Cagliari, S.S. 554 bivio per Sestu, 09042 Monserrato, Cagliari, Italy; agarau@unica.it (A.G.); gpicci@unica.it (G.P.); marca@unica.it (M.A.); ccaltagirone@unica.it (C.C.); gdefilip@unica.it (G.D.F.); isaia@unica.it (F.I.); apintus@unica.it (A.P.); scorciapino@unica.it (M.A.S.); aragoni@unica.it (M.C.A.); 2 School of Chemistry, University of Nottingham, University Park, Nottingham NG7 2RD, UK; alexanderjohnblake@outlook.com; 3 Dipartimento di Chimica, Università degli Studi di Milano, via Golgi 19, 20133 Milano, Italy; francesco.demartin@unimi.it

**Keywords:** macrocyclic ligands, palladium, platinum, Rhodium, DFT-Calculations

## Abstract

This study investigates the coordination chemistry of the tetradentate pyridine-containing 12-membered macrocycles **L^1^**-**L^3^** towards Platinum Group metal ions Pd^II^, Pt^II^, and Rh^III^. The reactions between the chloride salts of these metal ions and the three ligands in MeCN/H_2_O or MeOH/H_2_O (1:1 *v*/*v*) are shown, and the isolated solid compounds are characterized, where possible, by mass spectroscopy and ^1^H- and ^13^C-NMR spectroscopic measurements. Structural characterization of the 1:1 metal-to-ligand complexes [Pd(**L^1^**)Cl]_2_[Pd_2_Cl_6_], [Pt(**L^1^**)Cl](BF_4_), [Rh(**L^1^**)Cl_2_](PF_6_), and [Rh(**L^3^**)Cl_2_](BF_4_)·MeCN shows the coordinated macrocyclic ligands adopting a folded conformation, and occupying four coordination sites of a distorted square-based pyramidal and octahedral coordination environment for the Pd^II^/Pt^II^, and Rh^III^ complexes, respectively. The remaining coordination site(s) are occupied by chlorido ligands. The reaction of **L_3_** with PtCl_2_ in MeCN/H_2_O gave by serendipity the complex [Pt(**L^3^**)(μ-1,3-MeCONH)PtCl(MeCN)](BF_4_)_2_·H_2_O, in which two metal centers are bridged by an amidate ligand at a Pt1-Pt2 distance of 2.5798(3) Å and feature one square-planar and one octahedral coordination environment. Density Functional Theory (DFT) calculations, which utilize the broken symmetry approach (DFT-BS), indicate a singlet *d*^8^-*d*^8^ Pt^II^-Pt^II^ ground-state nature for this compound, rather than the alleged *d*^9^-*d*^7^ Pt^I^-Pt^III^ mixed-valence character reported for related dinuclear Pt-complexes.

## 1. Introduction

Macrocyclic chemistry is a very important and active area of chemical science with implications in a wide variety of applications, such as analytical chemistry, separation science, catalysis, and medicinal chemistry [1,2,3,4,5,6], and also in the development of fundamental aspects of supramolecular chemistry, such as molecular recognition, host-guest interactions, design of sensors, and smart artificial molecular devices [7,8,9,10].

Novel macrocyclic chemical structures—differing in molecular shape, architecture, flexibility, arrangement of structural groups, binding sites, and reactive functions—continue to be developed, with the aim of improving performances in the chemical functions of interest by achieving better control over the strength, selectivity, and dynamics of the binding processes of a variety of cationic, anionic, neutral, organic, and inorganic substrates.

However, the basic aspects of coordination chemistry of macrocyclic ligands towards different substrates, particularly metal ions, continue to be a fascinating area of research in the quest for systems capable of forcing the metal center to adopt unusual coordination geometries and/or oxidation states within stable complexes. For this purpose, the hard-soft nature of donor atoms and their spatial disposition, the cavity size, and flexibility of macrocyclic ligands are the most important parameters that define the coordination properties of these systems in relation to the stereo-electronic requirements of the metal ions of interest [11,12].

In this context, we have been engaged in the development of mixed N/O/S-donor macrocycles featuring rigid heterocyclic moieties, such as pyridine (*py*) [13,14,15,16,17,18,19,20,21], and 1,10-phenanthroline (*phen*) [22,23,24,25,26,27,28,29,30,31] as integral parts of the macrocyclic structure, which is completed by an aliphatic portion carrying different donor atoms. These systems proved to be highly efficient and selective ionophores in solid-phase extraction, selective transport, preparation of PVC-based ion-selective electrodes, and fluorimetric chemosensors for some transition and heavy metal ions. On the other hand, the conformational constraints on the aliphatic portion of these cyclic systems determined by the rigid heteroaromatic moieties, along with the fact that these heteroaryl frameworks carry one or more borderline N-donor atoms and are excellent π-acceptors groups, can be useful factors in expanding the scope of forcing unusual coordination behaviors, especially on *d*^8^ transition metal ions, such as Pd^II^ and Pt^II^, having very strict stereo-electronic requirements [32,33].

**Figure 1:**
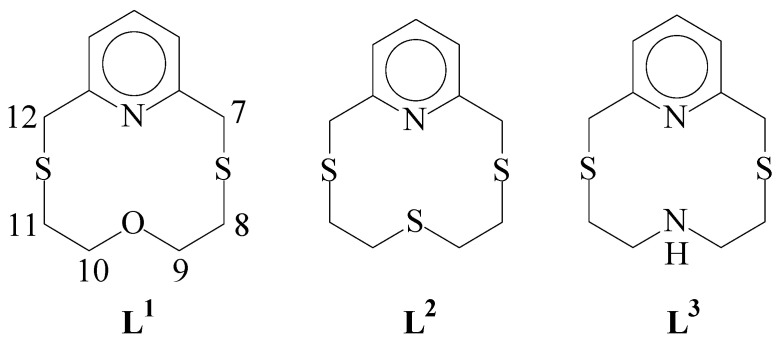
Pyridine-based macrocyclic ligands are considered in this study.

We report herein the results of our investigation of the coordination chemistry of tetradentate macrocycles **L^1^**-**L^3^** (Figure 1) to platinum group metals, particularly Pd^II^, Pt^II^, and Rh^III^. **L^1^**-**L^3^** contain one N- and two S-donor atoms as present in *bis*(thiomethyl)pyridine and complete their donor set with an aliphatic linker featuring the extra donor atom O(**L^1^**), S(**L^2^**), and NH(**L^3^**) (Figure 1). Although these ligands, especially **L^3^**, have found interesting applications in the development of fluorescent chemosensors for heavy metal ions [13,14,15,16,17,18,19,20,21], their coordination chemistry is largely undeveloped.

In fact, in the case of **L^1^**, only the X-ray crystal structure of the neutral complex [Cu(**L^1^**)Cl_2_] is known [34], while for **L^3^**, only the 1:1 complex cations [Cu(**L^3^**)]^2+^ and [Zn(**L^3^**)]^2+^ have been reported as nitrate salts [13]. In the case of **L^2^**, the polymeric complexes [Ag(**L^2^**)]_n_(CF_3_CO_2_)_n_·nH_2_O [35] and [Ag(**L^2^**)]_n_(NO_3_)_n_ [36] were structurally characterized along with the discrete 1:1 complex cations [Cu(**L^2^**)]^2+^ [35] and [Hg(**L^2^**)Cl]^+^ [37] (NO_3_^−^ and HgCl_3_^−^ counter-anions, respectively), and the binuclear compound [Ni_2_(**L^2^**)_2_Cl_2_](BF_4_)_2_·1.5MeNO_2_ [22].

The present study is strictly related to the previous ones performed on macrocyclic ligands similar to **L^1^** and **L^2^** but featuring the *phen* moiety instead of the *py* unit [22,32,33,38]. In those cases, the nature of the donor atom sets, the conformational constraints determined by the *phen* unit on the thioether linkers of the two pentadentate rings, and the locked [4+1] coordination sphere imposed on the Pd^II^ and Pt^II^ ions in their 1:1 complexes were responsible for the stabilization of the corresponding low-valent complexes of Pd^I^ and Pt^I^ [32,33]. The crystal structures of **L^1^** and **L^2^** are known [34,39]. In both structures, the aliphatic chain of the rings is tilted over the plane containing the pyridine unit—presumably because of the repulsion between the two sulfur atoms close to the aromatic ring [34]. The other donor atom, independently of its nature (oxygen in the case of **L^1^** and sulfur in the case of **L^2^**), adopts exodentate orientations with the lone pairs of electrons (LPs) pointing out of the ring cavity. Therefore, a conformational change is required for these ligands (and presumably also for **L^3^**) to coordinate a metal center with all four donor atoms. A similar situation is observed in the case of the analog of **L^1^** but featuring a *phen* moiety instead of the *py* unit, in the free ligand for which the crystal structure is known [33]. In this case, the explanation given for the conformational behavior observed for **L^1^** and **L^2^** cannot be applied, as the S-donors would be too far apart even in a completely planar conformation of the ligand. The tendency of the LPs on the S-donors to occupy exodentate positions pointing out of the ring cavity, with the effect of maximizing the number of *gauche* placements about the C-S bonds, seems more likely to be responsible for the tilted conformation also observed in the *phen* analogous of **L^1^**. In both kinds of macrocyclic ligand, therefore, a conformational change in the aliphatic chains is required upon coordination to bring the lone pair(s) of all donors to adopt endodentate orientations suitable for metal coordination [13,22,32,33,34,35,36,38,39].

## 2. Results

### 2.1. Coordination Chemistry of **L^1^** towards Pd^II^, Pt^II^, Rh^III^

The reaction of **L^1^** with one molar equivalent of PdCl_2_ in refluxing MeOH/H_2_O (1:1 *v*/*v*), followed by reduction of the volume of the reaction mixture under vacuum and slow evaporation in the air of the remaining solvent (water), afforded reddish prismatic crystals. Analytical data (Fast Atom Bombardment (FAB) Mass Spectrum, Figure S1 in the Supplementary Materials (SM), and elemental analysis in the Materials and Methods Section) indicate a Pd/**L^1^** molar ratio higher than 1:1 in the obtained compound. 

The ^13^C-NMR spectrum of the complex recorded in CD_3_CN solution at 25 °C shows only three peaks for the aromatic fragment of the macrocyclic ligand (δ_C_ = 122.8, 140.5, 164.1 ppm) and three for the aliphatic chain (δ_C_ = 45.0, 45.9, 65.4 ppm, Figure S2 in SM), thus suggesting that the complex exists in solution in only one form having a C_s_ symmetry with a symmetry plane passing through the N-donor atom of the ligand.

With respect to the free macrocycle, the carbon atoms next to S-donors are deshielded [δ_C_ = 45.0 (36.6) and 45.9 (30.2) ppm for C7/C12 and C8/C11, respectively; see Figure 1 for the numbering scheme adopted, values in parentheses refer to the free macrocycle], whereas those next to the aliphatic O-donor are slightly shielded [δ_C_ = 65.4 (66.7) ppm for C9/C10; see Figure 1]. The ^13^C-NMR chemical shifts do not change on changing the temperature in the range allowed by the solvent CD_3_CN. These data are consistent with a coordination sphere imposed in solution by **L^1^** at the Pd^II^ with possibly the O-donor atom weakly interacting with the metal center.

The ^1^H-NMR spectrum of the complex recorded in CD_3_CN solution at 25 °C (Figure S3) exhibits six distinct groups of aliphatic protons at 2.74–2.78 (multiplet), 3.32–3.34 (multiplet), 3.54–3.59 (multiplet), 4.07–4.10 (multiplet), 4.45 (doublet) and 4.88 (doublet) ppm each integrating for 2 protons, the assignments of which have been made on the basis of ^1^H-^13^C-NMR Heteronuclear Single Quantum Correlation (HSQC) experiments in CD_3_CN (see Experimental Section and Figure S4). 

The doublets at 4.45 and 4.88 ppm are due to an AB spin system (*J* = 18.5 Hz) for each pair of protons on C7 and C12. This is confirmed by the observation in the HSQC that both doublets correlate with the same ^13^C resonance at 45.0 ppm. The same is found for the multiplets mentioned above, whose multiplicity is indicative of an AA’BB’ spin system. The four multiplets can be distinguished in two pairs, each correlating with a single ^13^C resonance in the HSQC spectrum. These observations indicate the presence of a symmetry plane bisecting the pyridine ring and passing through the metal ion in the complex structure, while the two geminal protons in each of the methylene groups are magnetically inequivalent. This is in agreement with inequivalent dispositions assumed by the protons on C7 and C12 (above and below the plane of the pyridine moiety) as a consequence of the ligand complexation.

**Figure 2:**
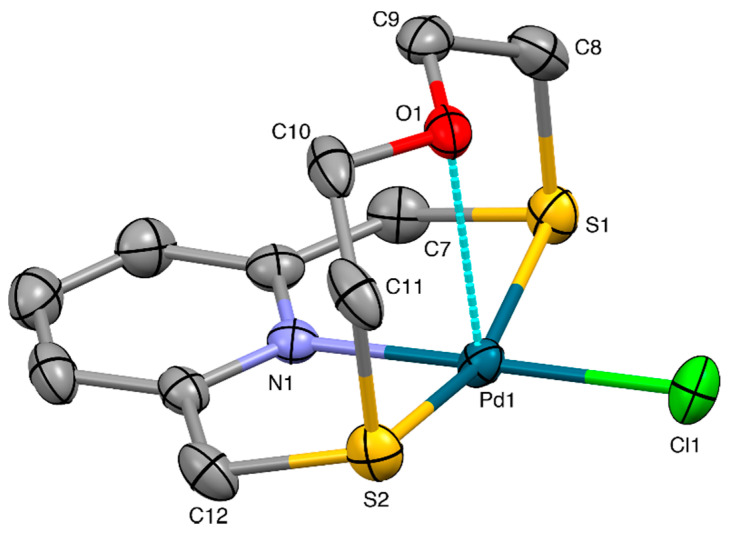
View of the [Pd(**L^1^**)Cl]^+^ complex cation in [Pd(**L^1^**)Cl]_2_[Pd_2_Cl_6_] with the numbering scheme adopted. Displacement ellipsoids are drawn at a 30% probability level. H-atoms are omitted for clarity. Selected bond distances (Å): Pd1-N1 2.013(3), Pd1-S1 2.3062(10), Pd1-S2 2.2915(10), Pd1-Cl1 2.2984(11), Pd1-O1 2.654(3); angles (°): N1-Pd1-S1 86.52(9), N1-Pd1-S2 86.17(9), N1-Pd1-Cl1 179.14(9), N1-Pd1-O1 89.5(1), S1-Pd1-S2 162.32(4), S1-Pd1-Cl1 94.34(4), S1-Pd1-O1 81.69(6), S2-Pd1-Cl1 92.99(4), S2-Pd1-O1 82.17(6), O1-Pd1-Cl1 90.57(6).

An X-ray diffraction analysis was undertaken on the isolated reddish crystals to ascertain the ligation and stereochemistry of this complex. The crystal structure confirms the formation of the compound [Pd(**L^1^**)Cl]_2_[Pd_2_Cl_6_] containing [Pd(**L^1^**)Cl]^+^ complex cations (Figure 2) balanced by [Pd_2_Cl_6_]^2–^ counter-anions and with the tetradentate macrocyclic ligand imposing a [3+1] coordination sphere at the Pd^II^. The N-donor of the pyridine unit, Pd1-N1 2.013(3) Å, the two S-donors of the aliphatic linker, Pd1-S1 2.3062(10), Pd1-S2 2.2915(10) Å, and a Cl^−^ ligand, Pd1-Cl1 2.2984(11) Å are bound in a square-planar arrangement to the metal atom. The O-donor occupies an apical site at a distance of 2.654(3) Å from the metal center, which is much less than the sum (3.10 Å) of the relevant van der Waals radii [40]. A similar type of Pd···O interaction [2.779(4) Å] was observed in the complex cation [Pd([15]aneN_2_OS_2_)]^2+^ ([15]aneN_2_OS_2_ = 1-oxa-7,10-dithia-4,13-diazacyclopentadecane) [41], while much longer Pd···O interactions were found in the 1:1 complex of Pd^II^ with the pentadentate macrocyclic ligand similar to **L^1^**, but having a *phen* unit instead of the *py* moiety [2.935(4) Å with the O-donor lying above the N_2_PdS_2_ coordination plane] [33], and in the half-sandwich complex [Pd([9]aneS_2_O)Cl_2_] ([9]aneS_3_ = 1,4,7-trithiacyclononane), in which the macrocyclic ligand assumes a facial [2S + O] coordination mode at the metal center with the oxygen atom lying above the Cl_2_PdS_2_ coordination plane at a Pd···O distance of 2.968(3) Å [42]. The Pd-O vector is almost perpendicular to the Pd^II^ coordination plane as a consequence of the folded conformation adopted by **L^1^** in the complex cation [Pd(**L^1^**)Cl]^+^ (Figure 2), which resembles an open book with the spine along the line connecting the S1-Pd1-S2 atoms and the N1-Pd1-O1 hinge angle of 89.5(1)°.

The charge neutrality of the complex is guaranteed by the dinuclear planar [Pd_2_Cl_6_]^2–^ anion featuring two Pd^II^ metal centers in a square-planar coordination sphere, which is determined by four coordinated chloride anions, two of which bridging the metal ions.

The [Pd_2_Cl_6_]^2–^ anions, which lie on a crystallographic inversion center, are sandwiched by two [Pd(**L^1^**)Cl]^+^ cations and interact with them through CH···Pd and CH···Cl interactions of 2.67 and 2.71 Å, respectively (Figure S5 in SM). CH···Cl interactions among anions and complex cations ranging between 2.76 and 2.89 Å (Figure S6), together with intermolecular CH···Cl (2.73, 2.85 Å) and CH···O (2.80 Å) interactions among complex cations, contribute to determine the crystal packing in this compound (Figures S6 and S7).

Following the same synthetic procedures adopted for the synthesis of [Pd(**L^1^**)Cl]_2_[Pd_2_Cl_6_], we reacted **L^1^** with PtCl_2_ in refluxing MeOH/H_2_O (1:1 *v*/*v*). Yellow crystals were obtained after the addition of excess NH_4_BF_4_ to the reaction mixture, evaporation of MeOH under the vacuum, and subsequent crystallization in the air of the remaining aqueous solution by slow evaporation. The FAB mass spectrum of the compound (Figure S8) exhibits peaks with the correct isotopic distribution for [Pt(**L^1^**)Cl]^+^ (*m/z* = 472). These data, together with elemental analysis, confirm the formulation [Pt(**L_1_**)Cl](BF_4_) for the isolated compound. ^1^H- and ^13^C-NMR spectra (Figures S9 and S10, respectively) of the complex in CD_3_CN show features very similar to those observed for [Pd(**L^1^**)Cl]_2_[Pd_2_Cl_6_], including the evidence of the AB spin system (*J* = 18.0 Hz) for the doublets at 4.62 and 4.85 ppm for each pair of protons on C7 and C12, respectively, and of the AA’BB’ spin system for the other methylene groups resonating at a lower frequency (assignments are made on the basis of ^1^H-^13^C-NMR HSQC experiments in CD_3_CN, Figure S11). This strongly suggests a very similar structure for the complexes formed with Pd^II^ and Pt^II^. Furthermore, the carbon atoms next to S-donors are deshielded [δ_C_ = 46.7 (36.6) and 47.4 (30.2) ppm for C7/C12 and C8/C11, values in parentheses refer to the free macrocycle], whereas those next to the aliphatic O-donor are slightly upshifted [δ_C_ = 66.4 (66.7) ppm for C9/C10].

**Figure 3:**
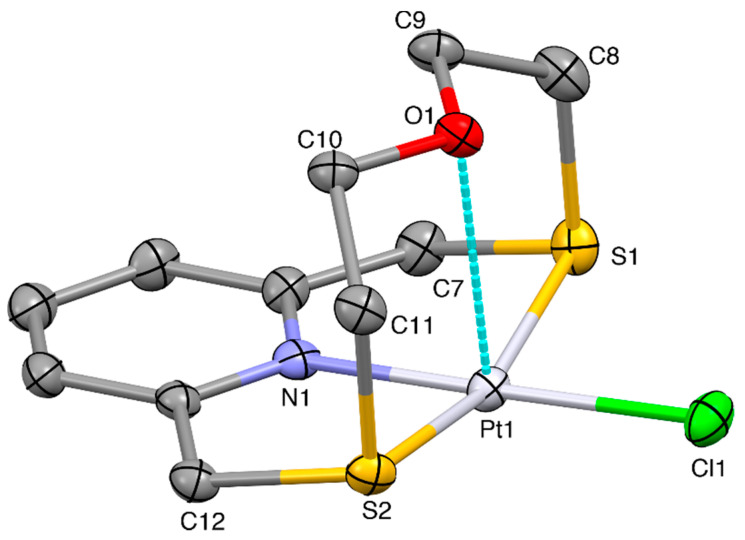
View of the [Pt(**L^1^**)Cl]^+^ complex cation in [Pt(**L^1^**)Cl](BF_4_) with the numbering scheme adopted. Displacement ellipsoids are drawn at a 30% probability level. H-atoms are omitted for clarity. Selected bond distances (Å): Pt1-N1 2.010(4), Pt1-S1 2.2753(14), Pt1-S2 2.2804(14), Pt1-Cl1 2.3008(15), Pt1-O1 2.752(4); angles (°): N1-Pt1-S1 86.48(14), N1-Pt1-S2 86.95(14), N1-Pt1-Cl1 178.99(13), N1-Pt1-O1 89.5(2), S1-Pt1-S2 163.59(6), S1-Pt1-Cl1 92.76(6), S1-P1-O1 82.71(9), S2-Pt1-Cl1 93.61(6), S2-P1-O1 82.21(9), O1-Pt1-Cl1 89.80(9).

An X-ray diffraction analysis was undertaken on the obtained yellow crystals to ascertain the nature of this complex. The crystal structure confirms the formation of the complex cation [Pt(**L^1^**)Cl]^+^ (Figure 3) balanced by a BF_4_^−^ counter-anion.

**Figure 4:**
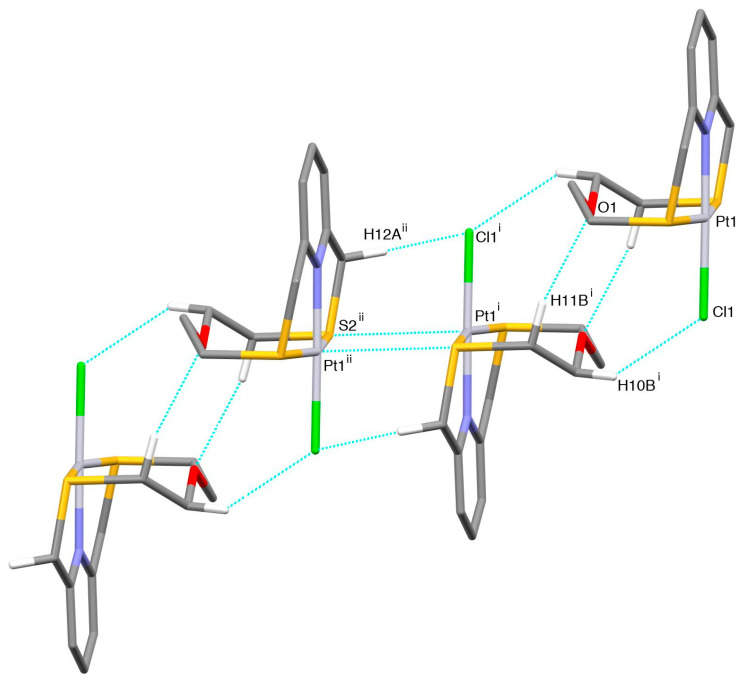
Partial view along the [−1,1,0] direction of the packing of [Pt(**L^1^**)Cl]^+^ cations in [Pt(**L^1^**)Cl](BF_4_). Only H atoms involved in the relevant H-bonds are shown for clarity. Dimers of the complex cation featuring C-H···O and C-H···Cl bonds [H10B^i^···Cl1 2.89, C10^i^···Cl1 3.751(7) Å, C10^i^-H10B^i^···Cl1 146°, H11B^i^···O1 2.43, C11^i^···O1 3.397(9)Å, C11^i^-H11B^i^···O1 167°] are held together by weak Pt1^i^···S^ii^ [Pt1^i^···S2^ii^ 3.625(2) Å] contacts and C-H···Cl H-bonds [H12A^ii^···Cl1^i^ 2.88, C12^ii^···Cl1^i^ 3.533(6) Å, C12^ii^-H12A^ii^···Cl1^i^ 124°] to form chains which run along the [001] direction. Symmetry codes: i = 1 − x, 1 − y, −z; ii = x, y, −1 + z.

The coordination environment at the metal center is very similar to that observed in the case of [Pd(**L^1^**)Cl]^+^ with the macrocyclic ligand adopting the typically folded conformation and imposing a [NS_2_+O] coordination sphere at the Pt^II^ metal ion, which reaches an overall square-based pyramidal geometry thanks to the coordination of a Cl^−^ ligand in the equatorial plane (Figure 3). The O-donor occupies the apical site of the square-pyramid at a distance of 2.752(4) Å from the metal center, which is slightly longer than the Pd···O distance observed in the complex cation [Pd(**L^1^**)Cl]^+^. In the crystal packing, two units of complex cation interact via Pt···S and CH···Cl contacts of 3.625(2) Å and 2.88 Å, respectively, with the relevant equatorial coordination planes facing each other (Figure 4). Dimers of this kind interact head-to-tail via CH···Cl and CH···O H-bonds of 2.89 and 2.43 Å, respectively, to form zig-zag chains running along the [001] direction.

The zig-zag chains of [Pd(**L^1^**)Cl]^+^ complex cations are joined in the crystal via CH···F H-bonds ranging from 2.38 to 2.48 Å involving BF_4_^−^ counter anions (Figure S12).

A synthetic procedure similar to that adopted for the synthesis of [Pt(**L^1^**)Cl](BF_4_) was also employed for the preparation of the 1:1 complex of **L^1^** with Rh^III^. Yellow crystals were obtained after the addition of excess NH_4_PF_6_ to the reaction mixture of RhCl_3_·H_2_O and **L^1^** in MeCN/H_2_O (1:1 *v*/*v*), removal of the solvent under vacuum, and crystallization of the resulting solid from MeCN by slow diffusion of Et_2_O vapors. The elemental analysis and the FAB mass spectrum (Figure S13) of the obtained crystals, which exhibits peaks with the correct isotopic distribution for [Rh(**L^1^**)Cl_2_]^+^ (*m/z* = 414), confirm the formulation [Rh(**L^1^**)Cl_2_](PF_6_) for the isolated compound. Similar to the case of Pd^II^ and Pt^II^, the ^1^H-NMR spectrum reflects an AB spin system for each pair of protons on C7 and C12 (doublets at 5.01 and 5.25 ppm with *J* = 18.6 Hz, Figures S14–S16) and AA’BB’ spin system for the other methylene protons (four multiplets at 4.04–4.07, 3.57–3.62, 3.48–3.51 and 3.36–3.40 ppm). The two doublets showed correlation with a single ^13^C resonance at 46.0 ppm in the HSQC spectrum (Figure S16). The four multiplets can be divided into two pairs, with the two ^1^H multiplets at 4.04–4.07 and 3.36–3.40 ppm showing correlation with a single ^13^C resonance at 74.1 ppm, while the other two ^1^H multiplets at 3.57–3.62 and 3.48–3.51 ppm showed correlation with the same ^13^C resonance at 40.5 ppm, in the HSQC spectrum. These observations strongly indicate a coordination mode of the ligand analogous to that observed in the Pd^II^ and Pt^II^ complexes of **L^1^**.

**Figure 5:**
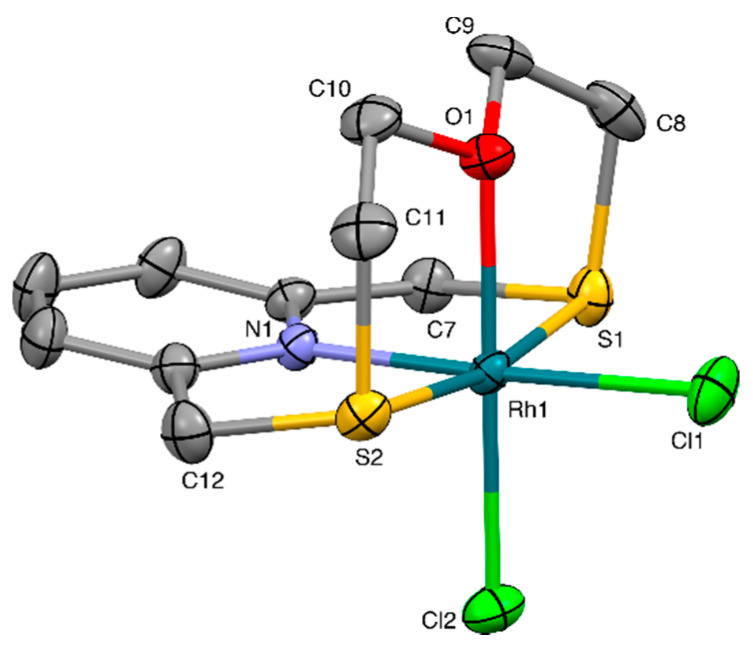
View of the [Rh(**L^1^**)Cl_2_]^+^ complex cation in [Rh(**L^1^**)Cl_2_](PF_6_) with the numbering scheme adopted. Displacement ellipsoids are drawn at a 30% probability level. H-atoms are omitted for clarity. Selected bond distances (Å): Rh1-N1 2.015(3), Rh1-O1 2.088(2), Rh1-S1 2.2851(10), Rh1-S2 2.3056(10), Rh1-Cl1 2.3318(11), Rh1-Cl2 2.3001(10); angles (°): N1-Rh1-O1 89.92(11), N1-Rh1-S1 87.20(8), N1-Rh1-S2 86.48(8), N1-Rh1-Cl1 177.55(9), N1-Rh1-Cl2 89.96(9), S1-Rh1-O1 86.60(7), S1-Rh1-Cl1 91.94(4), S1-Rh1-Cl2 92.57(4), S1-Rh1-S2 170.23(3), S2-Rh1-O1 85.96(7), S2-Rh1-Cl1 94.07(4), S2-Rh1-Cl2 94.86(4), O1-Rh1-Cl1 87.74(8), O1-Rh1-Cl2 179.16(7), Cl1-Rh1-Cl2 92.37(4).

An X-ray diffraction analysis was undertaken on the obtained yellow crystals showing the presence of [Rh(**L^1^**)Cl_2_]^+^ complex cations counterbalanced by PF_6_^−^ anions in the crystal structure. The complex cations feature a Rh^III^ ion in a distorted octahedral environment defined by the four donor atoms of a macrocycle **L^1^** and two chlorido ligands (Figure 5). The structure of the cation is conditioned by the meridional coordination of the 2,6-bis(thiomethyl)pyridine unit, as is observed in the value of the S-Rh-S angle, 170.23(3)°. The O-donor is located perpendicular to the *pseudo*-plane defined by the metal ion, the pyridine ring, and the two thioether sulfur atoms. The folded conformation, adopted by **L^1^** as in the cases of [M(**L^1^**)Cl]^+^ complex cations (M = Pd, Pt), leaves the two coordination sites occupied by two Cl^−^ ligands in a relative *cis* orientation. 

**Figure 6:**
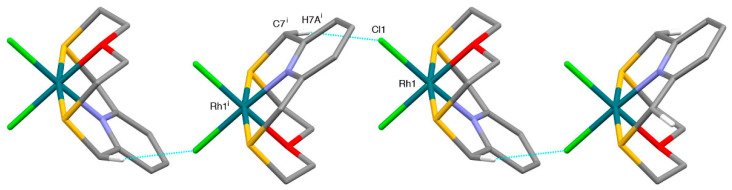
Partial view along the [100] direction of [Rh(**L^1^**)Cl_2_]^+^ complex cations joined head to tail via C-H···Cl bonds to form zig-zag chains running along the [010] direction in [Rh(**L^1^**)Cl_2_](PF_6_). Only H atoms involved in the relevant H-bonds are shown for clarity. Cl1···H7A^i^ 2.66, Cl1···C7^i^ 3.567(4) Å, Cl1-H7A^i^···C7^i^ 157°. Symmetry code: i = −x, −½ + y, 3/2 − z.

While the Rh-N, Rh-S, and Rh-Cl bond distances are similar to the corresponding bond lengths observed in the [M(**L^1^**)Cl]^+^ complex cations (M = Pd, Pt), the Rh-O bond length [2.088(2) Å] is much shorter, in agreement with the stereoelectronic requirements of Pd^II^, Pt^II^, and Rh^III^ in their coordination chemistry. In the crystal packing, [Rh(**L^1^**)Cl_2_]^+^ units are joined head to tail via CH···Cl H-bonds of 2.66 Å to form zig-zag chains, which run along the [010] direction (Figure 6). As in the case of the compound [Pd(**L^1^**)Cl](BF_4_), H-bonded chains of [Rh(**L^1^**)Cl_2_]^+^ units are joined via CH···F bonds of lengths 2.33–2.53 Å involving PF_6_^−^ counter-anions to afford a 3-dimensional network (Figure S17).

### 2.2. Coordination Chemistry of **L^2^** towards Pd^II^, Pt^II^, Rh^III^

The reaction of **L^2^** with one molar equivalent of PdCl_2_ or PtCl_2_ in refluxing MeCN/H_2_O (1:1 *v*/*v*) afforded orange and yellow microcrystalline powders, respectively (see Experimental Section). Unfortunately, we were not able to grow crystals suitable for X-ray diffraction analysis. However, mass spectra (Figures S18, S19) and elemental analyses suggest the formation of 1:1 metal-to-ligand complexes having the formulation [M(**L^2^**)Cl]Cl (M = Pd^II^ and Pt^II^). 

^1^H-NMR spectra of the complex [Pd(**L^2^**)Cl]Cl (Figure S21) and [Pt(**L^2^**)Cl]Cl (Figure S23) recorded in D_2_O and CD_3_CN, respectively, show features that are very similar to those observed for the respective complexes with **L^1^**, including the evidence of an AB spin system for each pair of protons on C7 and C12 (numbering scheme as in Figure 1) [doublets at 4.70 and 5.17 ppm (*J* = 18.8 and 18.4 Hz) for the Pd^II^ compound, 3.94 and 4.05 ppm (*J* = 12.8 Hz) for the Pt^II^ compound]. The carbon atoms next to the S-donors are all deshielded [δ_C_ = 46.9 (36.2), 48.0 (30.9), 33.2 (29.9) ppm for C7/C12, C8/C11, and C9/C10, respectively, in the case of the Pd^II^ complex (Figure S22, values in parentheses refer to the free macrocycle); 39.8, 39.2 and 34.3 ppm for the Pt^II^ complex (Figure S24)]. These results suggest a [3+1] coordination mode of **L^2^** at the Pd^II^ and Pt^II^ metal centers within a square-based coordination sphere, similar to those observed for **L^1^** in the case of the complex cations [Pd(**L^1^**)Cl]^+^ and [Pt(**L^1^**)Cl]^+^ (see above), respectively, with presumably stronger interactions of the metal ions with the apical S-donor. Interestingly, the free macrocycle **L^2^** is reported to prefer a “chair-like” conformation in which the central S-donor is oriented in the opposite direction with respect to the site perpendicular to the plane containing the remaining NS_2_ donor set, and a conformational change is necessary to interact with the *d*_z_^2^ orbital of the coordinated transition metal ion [39].

No crystals could be grown for the brown solid isolated from the reaction of **L^2^** with one molar equivalent of RhCl_3_·H_2_O in MeCN/H_2_O (1:1 *v*/*v*) followed by the addition of excess NH_4_PF_6_. Elemental analysis and the FAB mass spectrum (Figure S20) of the isolated compound showing peaks with the correct isotopic distribution for both [Rh(**L^2^**)Cl_2_]^+^ (*m/z* = 430) and for [Rh(**L^2^**)Cl]^+^ (*m/z* = 396) suggest the presence in the solid crude product of a 1:1 complex having the formulation [Rh(**L^2^**)Cl_2_](PF_6_). 

Surprisingly, the ^1^H- and ^13^C-NMR spectra of the Rh^III^ complex (Figures S25 and S26, respectively) isolated with **L^2^** recorded in CD_3_CN clearly show two distinct complexes in solution. Two series of homologous resonances can be seen, both for the aromatic and the methylene protons. Homologous resonances differ in position and relative intensity, but share the same fine structure, with very similar *J* couplings. By the relative intensity of 1.3, we can distinguish one major and one minor species, separated by a ΔG of 0.65 kJ mol^−1^. As far as the methylene resonances are concerned, both species are characterized by two doublets around 5 ppm corresponding to an AB spin system and showing scalar correlation with the same ^13^C resonance in the HSQC spectrum (Figure S27). Both the species are characterized by four multiplets between 2.5 and 4.0 ppm corresponding to an AA’BB’ spin system and distinguished in couples by showing correlation with the same ^13^C resonance in the HSQC spectrum. Definitely, the two complexes appear to be very similar. Since **L^1^** formed the complex cation [Rh(**L^1^**)Cl_2_]^+^ with Rh^III^, the major and minor species observed for **L^2^** could be tentatively assigned to the *cis* and the *trans* configurations of the two coordinated chloride ions. However, the latter is not compatible with the AB spin system observed for each pair of protons on C7 and C12, which are expected to be equivalent and to appear as a singlet in the ^1^H-NMR spectrum. In order to clarify this point further and to characterize the two species in more detail, we acquired NOESY spectra on both the **L^1^** and **L^2^** complexes with Rh^III^. Their analyses (see SI for discussion and Figure S28 for the molecular model compatible with NMR measurements) clearly point out that in the case of **L^2^** and Rh^III^, the dichlorido complex is formed together with another species in which one chloride, at least, is substituted by some other ligands (presumably a solvent molecule, likely MeCN). All attempts to isolate the two complexes by chromatography were unsuccessful. Data available on the solid-state are not conclusive on the presence of the complex featuring only one coordinated chlorido ligand (see above). While the peak at *m*/*z* 430 can be unambiguously assigned to the species [Rh(**L^2^**)Cl_2_]^+^, the peak at *m*/*z* = 396, which can be assigned to [Rh(**L^2^**)Cl]^+^, could either derive from [Rh(**L^2^**)Cl_2_]^+^ by loss of one chlorido ligand or from the other complex by the loss of the coordinated solvent molecule.

### 2.3. Coordination Chemistry of **L^3^** towards Pd^II^, Pt^II^, Rh^III^

The reaction of **L^3^** with one molar equivalent of PdCl_2_ in refluxing MeCN/H_2_O (1:1 *v*/*v*), followed by the addition of excess NH_4_PF_6_, the reduction of the solvent volume under vacuum, and the slow evaporation in the air of the remaining solvent (water), afforded a brown microcrystalline powder. The FAB mass spectrum of the compound exhibits a peak at *m*/*z* = 381 with the correct isotopic distribution expected for the cation [C_11_H_16_ClN_2_PdS_2_]^+^ (see Figure S29 in the SM). This, together with elemental analytical data, support the formation of a 1:1 complex having the formulation [Pd(**L^3^**)Cl](PF_6_). 

Indeed, as already observed for the Pd^II^ complexes with **L^1^** and **L^2^** (see above), the ^13^C-NMR chemical shifts for the Pd^II^ complex with **L^3^** in CD_3_CN solution shows only three peaks for the aromatic region and three for the aliphatic chain (Figure S30), thus suggesting that the complex exists in solution in only one form having a C_s_ symmetry with a symmetry plane passing through the two N-donor atoms of the ligand. With respect to the free macrocycle, the carbon atoms next to S-donors are deshielded [δ_C_ = 45.8 (37.8) and 43.3 (31.7) ppm for C7/C12 and C8/C11, respectively; (values in parentheses refer to the free macrocycle)], whereas those next to the aliphatic N-donor are slightly deshielded [δ_C_ = 48.2 (47.0) ppm for C9/C10]. These features have also been observed in the ^13^C-NMR spectra of the 1:1 complexes of Pd^II^ and Pt^II^ with pentadentate macrocyclic ligands similar to **L^1^**-**L^3^**, but having a *phen* unit instead of the *py* moiety [32,33], which showed a [4 + 1] coordination sphere at the metal centers with the central donor atom in the aliphatic linker occupying the apical site of a distorted square-based pyramid with a long-range interaction to the metal atom, and the ligand adopting a folded conformation. A similar coordination mode of **L^3^** to the Pd^II^ center can be suggested for the complex cation [Pd(**L^3^**)Cl]^+^ in which the [4 + 1] pyramidal coordination sphere would be reached thanks to a chloride anion occupying one site in the basal coordination plane (see Pd^II^ and Pt^II^ complexes of **L^1^** and **L^2^** above). 

This hypothesis is supported by the ^1^H-NMR spectrum of the complex in D_2_O (Figure S31) that exhibits four distinct groups of aliphatic protons at 3.03–3.06 (multiplet), 3.54–3.60 (multiplet), 4.61 (doublet), and 5.07 (doublet) ppm integrating for four, four, two, and two protons, respectively. The doublets at 4.61 and 5.07 ppm define an AB spin system for each pair of protons on C7 and C12 (Figure 1) (*J* = 18.6 and 18.0 Hz, respectively, assignment of the chemical shift is made for the analogy with the ^1^HNMR shits observed for the Pd^II^, Pt^II^, and Rh^III^ complexes of **L^1^**, whose assignment is made via ^1^H-^13^C-heteronuclear correlation, HSQC, experiments, see below), which agrees with inequivalent dispositions assumed by these protons (above and below the plane of the pyridine moiety) as a consequence of metal complexation. 

The reaction of **L^3^** with one molar equivalent of PtCl_2_ in refluxing MeCN/H_2_O (1:1 *v*/*v*), followed by the addition of excess NH_4_BF_4_, the reduction of the solvent volume under vacuum, and the slow evaporation in the air of the remaining solvent (water), serendipitously afforded a few red-orange single crystals suitable for X-ray diffraction analysis. We repeatedly tried the complexation in MeCN/H_2_O and also under different experimental conditions changing the solvent mixture by replacing MeCN with other solvents (MeNO_2_/H_2_O, MeOH/H_2_O, THF/H_2_O), but no reaction occurred, and the sole unreacted ligand was always recovered.

**Figure 7:**
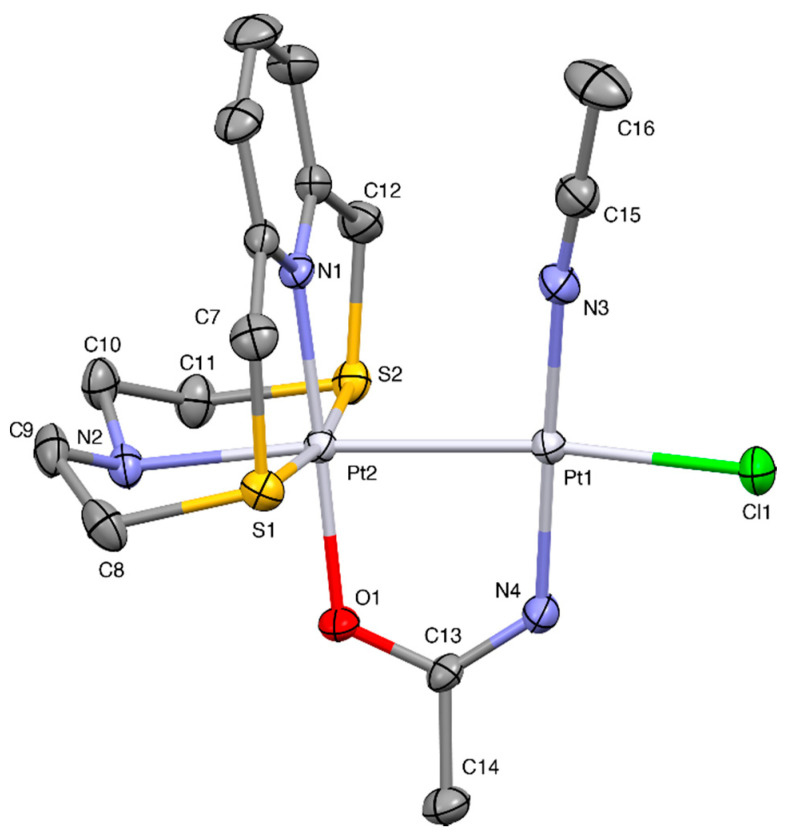
A view of the [Pt(**L^3^**)(μ-1,3-MeCONH)PtCl(MeCN)]^2+^ cation in [Pt(**L^3^**)(μ-1,3-MeCONH)PtCl(MeCN)](BF_4_)_2_·H_2_O with labelling scheme adopted. Displacement ellipsoids are drawn at a 30% probability level. H-atoms are omitted for clarity reasons. Selected bond distances (Å): Pt1-N3 1.971(4); Pt1-N4 1.981(4), Pt1-Cl1 2.3433 (11), Pt1-Pt2 2.5798(3), Pt2-N1 2.006(3), Pt2-O1 2.018(3), Pt2-N2 2.237(3), Pt2-S1 2.2926(10), Pt2-S2 2.3067(10), N4-C13 1.274(5), O1-C13 1.290(4); and angles (°): N3-Pt1-N4 176.46(15), N3-Pt1-Cl1 89.16(11), N3-Pt1-Pt2 98.39(11), N4-Pt1-Pt2 83.11(11), Cl1-Pt1-Pt2 172.44(3), N1-Pt2-O1 177.98(12), N1-Pt2-N2 91.81(13), O1-Pt2-N2 86.92(12), N1-Pt2-S1 87.28(9), O1-Pt2-S1 91.09(9), N2-Pt2-S1 87.27(9), N1-Pt2-S2 86.61(9), O1-Pt2-S2 94.86(9), N2-Pt2-S2 86.02(9), S1-Pt2-S2 170.77(4), N1-Pt2-Pt1 94.45(9), O1-Pt2-Pt1 86.86(8), N2-Pt2-Pt1 173.64(10), S1-Pt2-Pt1 94.19(3), S2-Pt2-Pt1 93.17(3).

The crystal structure determination revealed the unusual complex [Pt(**L^3^**)(μ-1,3-MeCONH)PtCl(MeCN)](BF_4_)_2_·H_2_O. The asymmetric unit consists of a binuclear [Pt(**L^3^**)(μ-1,3-MeCONH)PtCl(MeCN)]^2+^ complex cation involving a bridging amidate ligand likely formed from the hydrolysis of acetonitrile solvent [43,44,45] (Figure 7), BF_4_^−^ counter-anions and a co-crystallized water molecule. In the dimeric unit, one platinum atom is six-coordinated in a distorted octahedral geometry, being surrounded by the four donor atoms from the macrocyclic ligand **L^3^** [Pt2-N1 2.006(3), Pt2-N2 2.237(3), Pt2-S1 2.2926(10), Pt2-S2 2.3067(10) Å] the O-donor atom from the amidate bridge [Pt2-O1 2.018(3) Å], and the other platinum atom [Pt1-Pt2 2.5798(3) Å]. Pt1 is four-coordinated in a square-planar geometry, due to the additional coordination of an acetonitrile molecule [Pt1-N3 1.971(4) Å], a chlorido ligand [Pt1-Cl1 2.3433(11) Å], and the N-donor atom from the amidate bridging ligand [Pt1-N4 1.981(4) Å] (Figure 7). The maximum deviation from the least-squares plane calculated through the atoms Pt1, Cl1, N3, N4, Pt2 is 0.04 Å for N4. The average coordination plane at the Pt1 atom, which also comprises the two N-donors from the macrocyclic ligand and the two donors from the amidate bridging ligand, is almost perpendicular to the plane containing the pyridine ring and the Pt2, S1, S2, and O1 donors with the interplanar angle being 89.09° (Figure 7). **L^3^** adopts the folded conformation already observed in the complex cations [Cu(**L^3^**)]^2+^ and [Zn(**L^3^**)]^2+^ [13] resembling an open book with the spine along the line connecting the S1-Pt2-S2 atoms and the N1-Pt2-N2 hinge angle of 91.81(13)°. The aliphatic tertiary nitrogen is, therefore, located almost perpendicularly to the *pseudo*-plane defined by the metal ion, Pt2, the pyridine ring, the S-donors, and the amidate O-donor, in *trans*-position with respect to the other platinum atom, Pt1 (Figure 7). The four-coordinated platinum atom, Pt1, features the other platinum atom and the chlorido ligand in mutually *trans*-positions, giving an almost linear Cl-Pt-Pt-N arrangement in the binuclear cation, with the other two *trans*-positions being occupied by the coordinated acetonitrile molecule and the amidate N-donor. It is interesting to note that **L^3^** binds metal atoms with almost equivalent M-N1 and M-N2 bond lengths [13]. In contrast, in the complex cation, the bond distance Pt2-N2 = 2.237(3) Å is longer than the Pt2-N1 = 2.006(3) Å due to the higher *trans-*influence of coordinating Pt1 compared to O1 donor atoms, thus suggesting a donor–acceptor nature for Pt-Pt bond, also confirmed by the short Pt1-Pt2 distance [2.5798(3) Å] consistent with a metal-metal bond.

**Figure 8:**
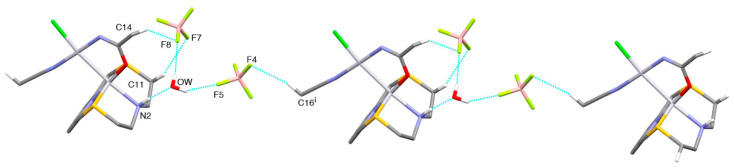
Partial view of complex cations interacting with BF_4_^−^ anions and H_2_O molecules to form chains extending along the [001] direction in [Pt(**L^3^**)(μ-1,3-MeCONH)PtCl(MeCN)](BF_4_)_2_·H_2_O. H-atoms not involved in H-interactions were omitted for clarity. N2H···OW 2.04(4), N2···OW 2.910(6) Å, N2-H···OW 174(4)°, F8···HW2 2.02(5), OW···F8 2.775(7) Å, OW-HW2···F8 145(4)°, F7···H11B 2.62, C11···F7 3.43(7) Å, C11-H11B···F7 141°, F8···H14A 2.48, C14···F8 3.283(5) Å, C14-H14A···F8 141°, F5···HW1 2.45(6), OW···F5 2.970(6) Å, OW-HW1···F5 119(6)°, F4···H16B^i^ 2.59, C16^i^···F4 3.52(7) Å, C16^i^-H16B^i^···F4 161°. Symmetry code: i = x, y, 1 + z.

The binuclear [Pt(**L^3^**)(μ-1,3-MeCONH)PtCl(MeCN)]^2+^ complex cations interact through H-bonds involving the BF_4_^−^ anions and the water molecule forming head-to-tail chains running along the crystallographic [001] direction (Figure 8). Symmetry-related chains pack through H-bonds involving the complex cations and the BF_4_^−^ anions (Figure S32).

Platinum binuclear complexes similar to [Pt(**L^3^**)(μ-1,3-MeCONH)PtCl(MeCN)]^2+^ are quite rare, the only other two known examples being the neutral two-electron mixed-valence complexes [Pt_2_^I,III^(tfepma)_2_X_4_] [X = Cl, Br; tfepma = *bis*(*bis*(trifluoroethoxy)-phosphino)methylamine] obtained by X_2_ photo-elimination from homobimetallic meridionally coordinated Pt^III^ tri-halides bridged by two neutral tfepma ligands, [Pt_2_^III,III^(tfepma)_2_X_6_] [46,47]. 

To get a deeper insight into the structural features of diplatinum-based discrete complexes, we have performed a search in the Cambridge Structural Database [48] for all compounds of this type containing a Pt-Pt bond, sorted on the coordination number around each platinum atom. 

**Figure 9:**
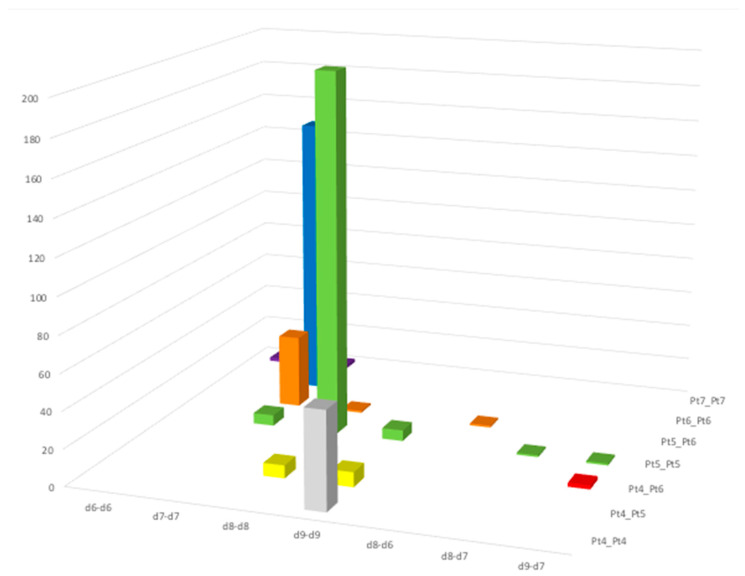
2D view of the number of structurally characterized diplatinum-based fragments formally belonging to *d^n^*-*d^n^* and mixed-valence *d^n^*-*d^m^* discrete binuclear complexes (*n*, *n* = 6, 7, 8, 9; *n*, *m* = 6, 8; 7, 8; and 7, 9) against the coordination number displayed by the platinum ions.

The structurally characterized diplatinum systems were assigned to the suites of *d^n^*-*d^n^* and mixed-valence *d^n^*-*d^m^* complexes (*n*, *n* = 6, 7, 8, 9; *n*, *m* = 6, 8; 7, 8; and 7, 9), based on the reported formal oxidation states. Figure 9 shows the number of fragments found for the different suites *versus* the coordination number displayed by the platinum centers.

Indeed, an examination of the data reported in Figure 9 shows that reportedly mixed-valence *d^n^*-*d^m^* complexes are quite rare, with only five examples known in the literature, among the 505 items found [49,50,51,52,53] beside the complexes [Pt_2_^I,III^(tfepma)_2_X_4_] (X = Cl, Br) [46,47]. The category with the higher number of items (212) is that of complexes formally featuring two penta-coordinated Pt^II^ ions, which can be envisaged as two square-planar complexes interacting through long Pt-Pt bonds ranging from 2.53 to 3.41 Å, with a mean value as long as 2.94 Å and an overall distorted square-based pyramidal environment for both metal ions. (*d*^8^-*d*^8^, Pt5-Pt5 green column in Figure 9). Quite numerous (155) are also the binuclear complexes formally featuring two Pt^III^ ions sharing one of the six bonds in a distorted octahedral coordination for both metals, with Pt^III^-Pt^III^ bond lengths in the range 2.39–3.08 and a mean value of 2.61 Å (*d*^7^-*d*^7^, Pt6-Pt6 blue column in Figure 9). For this kind of binuclear system, a significant number of structures (40 items) featuring the two metal ions in a different coordination environment (distorted octahedral/square-based pyramidal) are reported (*d*^7^-*d*^7^, Pt5-Pt6 orange column in Figure 9). These are often described as formal Pt^III^-Pt^III^ dimers with significant Pt^IV^ and Pt^II^ influences for the octahedral and square pyramidal platinum center, respectively [52]. The Pt-Pt distances again occupy a quite narrow range of 2.50–2.85 Å with a mean value of 2.69 Å.

Discrete dimers formally featuring a Pt^I^-Pt^I^ bond can be found in complexes featuring distorted square-planar/square-planar, square-planar/square-based pyramidal, and square-based pyramidal/square-based pyramidal coordination environments and ligands able to stabilize low oxidation states, such as phosphine derivatives, carbon monoxide, cyanides, hydrides and carbanions, and comprise metal-metal distances in the quite narrow range 2.53–2.76 Å, with a mean value of 2.62 Å (*d*^9^-*d*^9^ columns in Figure 9, 67 items). Only two binuclear complexes are known showing hepta-coordinated platinum Pt^III^-Pt^III^ or Pt^IV^-Pt^IV^ metal ions (Pt7-Pt7 purple columns in Figure 9) [54,55].

The complex [Pt(**L^3^**)(μ-1,3-MeCONH)PtCl(MeCN)](BF_4_)_2_·H_2_O belongs to the very unusual category of discrete Pt-dimers, featuring one square-planar and one octahedral platinum center connected by a metal-metal bond (*d*^9^-*d*^7^, Pt4-Pt6 red column in Figure 9) and formally considered as mixed-valence Pt^I^-Pt^III^ systems. It is interesting to note that the only two examples known of binuclear complexes formally sharing a Pt^I^-Pt^III^ bond, namely, [Pt_2_^I,III^(tfepma)_2_X_4_] [X = Cl, Br; tfepma = *bis*(*bis*(trifluoroethoxy)-phosphino)methylamine], contain the same trifluoroethyl-imidophosphito ligand bridging the metal centers counterbalanced by halides that complete the platinum coordination spheres [46,47]. Our compound would be the first example supported by a macrocyclic ligand that does not bridge the two metal centers. In these complexes the Pt-Pt distance is quite short [2.6187(7) and 2.6270(9) Å for X = Cl and Br, respectively], and the coordination environment is distorted octahedral for the reportedly Pt^III^ center and square-planar for the Pt^I^ one. This structural feature seems to be peculiar to formally defined *d*^9^-*d*^7^ Pt_2_^I,III^ binuclear complexes, In fact, *d*^7^-*d*^7^ binuclear Pt_2_^III,III^ complexes, also characterized by short metal-metal distances, see above, generally feature both metal centers either in distorted octahedral environments or octahedral/square-based pyramidal coordination spheres. In contrast, binuclear *d*^8^-*d*^8^ Pt_2_^II,II^ complexes are characterized by both metal centers in a distorted square-based pyramidal environment.

Following a synthetic procedure analogous to that adopted for the preparation of [Pd(**L^3^**)Cl](PF_6_), we were able to isolate a compound corresponding to the formulation [Rh(**L^3^**)Cl_2_](BF_4_)·MeCN from the reaction of **L^3^** with RhCl_3_·H_2_O in MeCN/H_2_O (1:1 *v*/*v*) (see Materials and Methods section and Figure S33 for FAB Mass Spectrum). ^13^C- and ^1^H-NMR spectra (Figures S34 and S35, respectively) presented features similar to those observed in the corresponding NMR spectra of [Rh(**L^1^**)Cl_2_](PF_6_), suggesting that the complex of Rh^III^ with **L^3^** has structural features similar to those found for [Rh(**L^1^**)Cl_2_](PF_6_). An X-ray diffraction analysis was undertaken on the crystals obtained by slow diffusion of Et_2_O vapors into a MeCN solution of the crude product.

**Figure 10:**
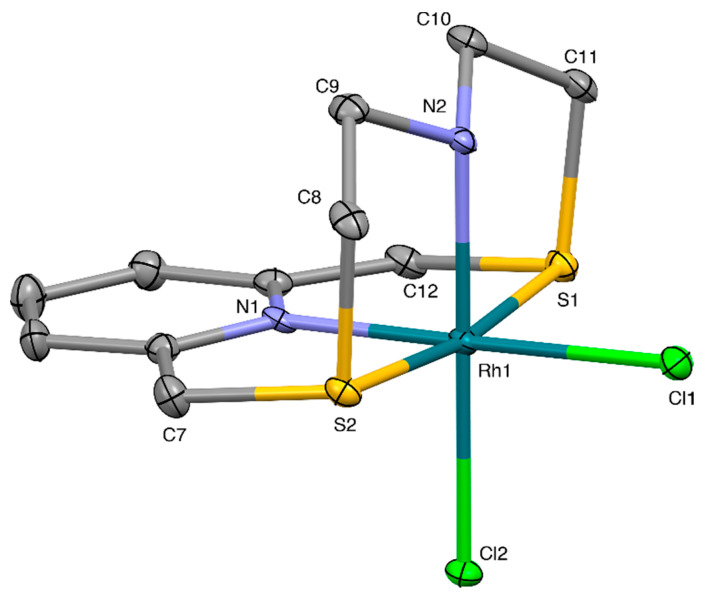
View of the [Rh(**L^3^**)Cl_2_]^+^ complex cation in [Rh(**L^3^**)Cl_2_](BF_4_)·MeCN with the numbering scheme adopted. Displacement ellipsoids are drawn at a 30% probability level. H-atoms are omitted for clarity. Selected bond distances (Å): Rh1-N1 2.018(5), Rh1-N2 2.047(5), Rh1-S1 2.3124(15), Rh1-S2 2.3010(15), Rh1-Cl1 2.3511(15), Rh1-Cl2 2.3515(14); angles (°): N1-Rh1-N2 91.72(19), N1-Rh1-S1 86.99(15), N1-Rh1-S2 87.20(15), N1-Rh1-Cl1 178.94(14), N1-Rh1-Cl2 88.24(14), S1-Rh1-N2 87.20(15), S1-Rh1-Cl1 93.31(6), S1-Rh1-Cl2 92.94(6), S1-Rh1-S2 171.78(5), S2-Rh1-N2 87.17(15), S2-Rh1-Cl1 92.40(6), S2-Rh1-Cl2 92.68(6), N2-Rh1-Cl1 87.29(14), N2-Rh1-Cl2 179.84(17), Cl1-Rh1-Cl2 92.76(5).

Indeed, the *pseudo*-octahedral coordination environment around the Rh^III^ metal center in the complex cation [Rh(**L^3^**)Cl_2_]^+^ (Figure 10) resembles that observed for [Rh(**L^1^**)Cl_2_]^+^ (Figure 5), with small variations on the structural parameters (bond distances and angles). In both complex cations [Rh(**L**)Cl_2_]^+^ (**L** = **L^1^**, **L^3^**), the ligands adopt a similar folded conformation upon coordination, leaving the other two coordination sites in a relative *cis* orientation occupied by two Cl^−^ ligands. 

**Figure 11:**
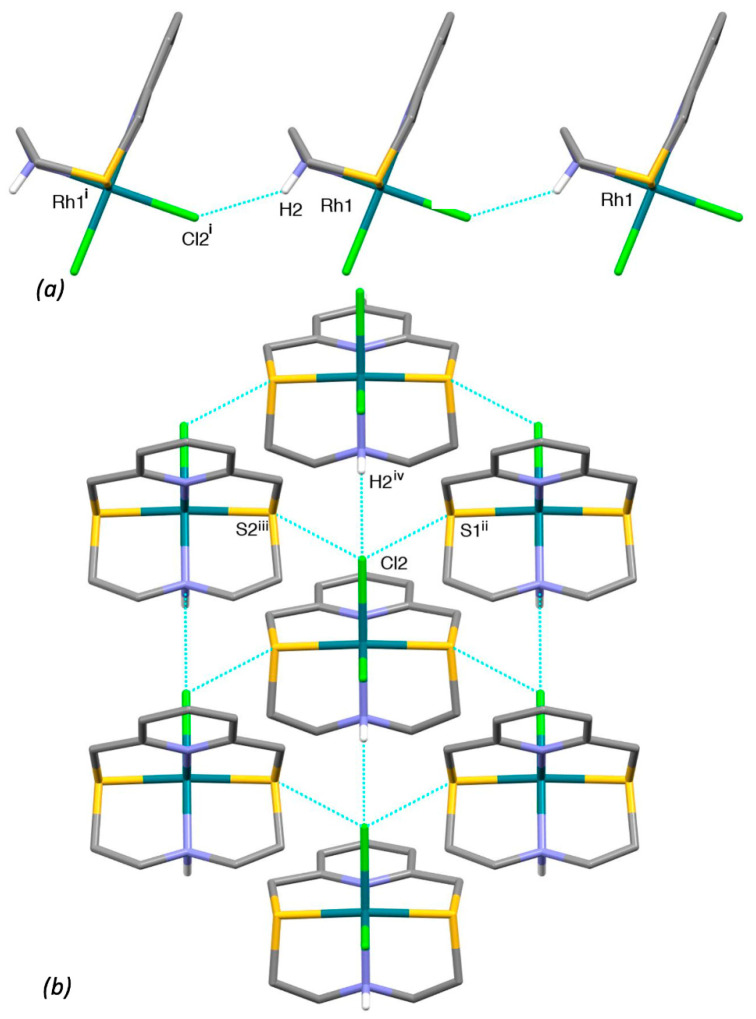
(**a**) Partial view of head-to-head interacting [Rh(**L^3^**)Cl_2_]^+^ complex cations in [Rh(**L^3^**)Cl_2_](BF_4_)·MeCN to form chains running along the [100] direction: N2-H2···Cl2^i^ 2.45 Å, N2···Cl2^i^ 3.235(5) Å, N2-H2···Cl2^i^ 142°; (**b**) partial view along the [101] direction of an undulated sheet lying in the (101) plane and formed by weak soft-soft interactions between [Rh(**L^3^**)Cl_2_]^+^ complex cations: Cl2···S2^ii^ 3.478(2), Cl2···S1^iii^ 3.483(2) Å. Symmetry codes: i = −1 + x, y, z; ii = ½ + x, ½ − y, ½ + z; iii = ½ + x, ½ − y, − ½ + z; iv = 1 + x, y, z.

In the crystal, [Rh(**L^3^**)Cl_2_]^+^ complex units are joined in chains running along the [100] direction via NH2···Cl2’ H-bonds (Figure 11a). Chains of complex cation interact with each other via soft-soft Cl···S weak interactions to form undulated sheets in the (101) plane (see Figure 11b).

Sheets of this kind stack along the [010] direction and are connected through an intricate network of C-H···F hydrogen bonds involving the BF_4^−^_ counter-anions. The MeCN molecules sit in between the layers and are anchored to the complex cation via an H-bond: N1S···H9^v^ 2.565 Å, N1S···C9^v^ 3.435(11) Å, N1S···H9^v^-C9^v^ 146° (v = −x, −y, 1 − z).

#### DFT Calculations on the Complex Cation [Pt(**L^3^**)(μ-1,3-MeCONH)PtCl(MeCN)]^2+^

Theoretical calculations carried out at the Density Functional Theory (DFT) [56] level represent an invaluable tool in understanding the electronic structure of metal complexes. They have been extensively used by chemists to investigate structure/property relationships in a large variety of compounds, in fields as varied as material science and bioinorganic chemistry [57,58,59,60], including metal···metal interactions [61,62,63,64]. Within the variety of DFT methods available, the broken symmetry approach (DFT-BS) proposed by Noodleman [65,66,67], widely used in the field of molecular magnetism for the calculation of exchange coupling constants in multinuclear complexes [68], allows unrestricted calculations for low-spin open-shell molecular systems, in which the α and β electrons are allowed to be localized on different atomic centers. This approach was applied to the complex cation [Pt(**L^3^**)(μ-1,3-MeCONH)PtCl(MeCN)]^2+^ and the previously reported related system [Pt_2_(tfepma)_2_Cl_4_] [46,47] in an attempt to elucidate the oxidation state of the platinum ions in these species. For the latter, a mixed-valence *d*^9^-*d*^7^ Pt^I^-Pt^III^ nature was proposed for the binuclear system. Based on the excellent results previously obtained on several coordination compounds containing group 10 metal ions [69,70], the mPW1PW functional [71] was adopted, in combination with the full-electron split valence basis sets (BSs) def2-SVP [72,73] for all atomic species but Pt, for which the LANL08(f) basis set [74], including pseudopotentials, was adopted to keep into account relativistic effects of the core electrons [75]. 

The metric parameters of the complex cation [Pt(**L^3^**)(μ-1,3-MeCONH)PtCl(MeCN)]^2+^ and [Pt_2_(tfepma)_2_Cl_4_] were optimized, starting from crystal structure data, in their closed-shell singlet state (2S + 1 = 1; Figure S36a) and triplet ground-state (2S + 1 = 3, two unpaired electrons; Figure S36b). The complexes were then modeled at the DFT-BS level (Figure S36c), starting from different configurations with different combinations of charges (*Q* = +1, +2, +3) and spin multiplicities (2*S* + 1 = 1, 2, 3) assigned to the two Pt ions.

These combinations correspond to all the possible configurations for both *d*^8^-*d*^8^ Pt^II^-Pt^II^ [singlet configuration, I in Figure S36c, and triplet configuration, with the unpaired electrons either on the octahedrally (Pt*_O_*) or square-planar (Pt*_SP_*) coordinated Pt ions, II and III in Figure S36c, respectively] and mixed-valence *d*^9^-*d*^7^ Pt^I^-Pt^III^ systems (singlet and triplet configurations, with either Pt*_O_* or Pt*_SP_* carrying the *Q* = +1 charge; IV–VII in Figure S36c).

This scheme includes the mixed-valence configuration previously reported for [Pt_2_(tfepma)_2_Cl_4_], where the charge *Q* = +3 was assigned to the Pt*_O_* center, and the charge *Q* = +1 to the Pt*_SP_* one (VI–VII in Figure S36c). When the optimization of the two model compounds was performed starting from the electron density guess of ground-state configurations I–VII, all calculations converged to two geometries only, corresponding to the closed-shell singlet and triplet ground-states.

According to these results, it appears that the spin densities on the two metal centers in this type of binuclear complexes cannot be separately modeled, probably because of the close proximity of the Pt ions, therefore both should be better described as binuclear *d*^8^-*d*^8^ Pt^II^-Pt^II^ complexes. An examination of the optimized geometries in the singlet and triplet ground-states shows that for both complexes the total electronic energy of the geometry in the singlet state is lower than that in the triplet state (by 111.1 and 133.7 kJ mol^−1^ for [Pt(**L^3^**)(μ-1,3-MeCONH)PtCl(MeCN)]^2+^ and [Pt_2_(tfepma)_2_Cl_4_], respectively). Accordingly, a better agreement between the optimized geometry and the structural data was found for both complexes for the singlet ground-state. In the case of [Pt(**L^3^**)(μ-1,3-MeCONH)PtCl(MeCN)]^2+^, the bond lengths and angles of the optimized geometry in the singlet state differ from the experimental ones by less than 0.05 Å and 6°, respectively, with the sole exception of the Pt2-N2 bond distance, which is overestimated by 0.111 Å (Tables S1–S3, Figure S37). On the other hand, in the optimized geometry in the triplet state, a significant elongation (about 0.3 Å) of the Pt-S bonds within the coordination sphere of the octahedral Pt ion is observed, along with a divergence from the square-planar coordination geometry for Pt1, with a Cl1-Pt1-Pt2-N2 dihedral angle of 89.51° (Figure S37). In the case of [Pt_2_(tfepma)_2_Cl_4_], an even further deviation from the experimental geometry was observed for the optimized geometry in the triplet state, featuring both Pt ions pentacoordinated in a trigonal bipyramidal geometry, while a very good agreement was found between the experimental structure and that optimized in the singlet ground-state (Tables S4–S6, Figure S38). 

**Figure 12:**
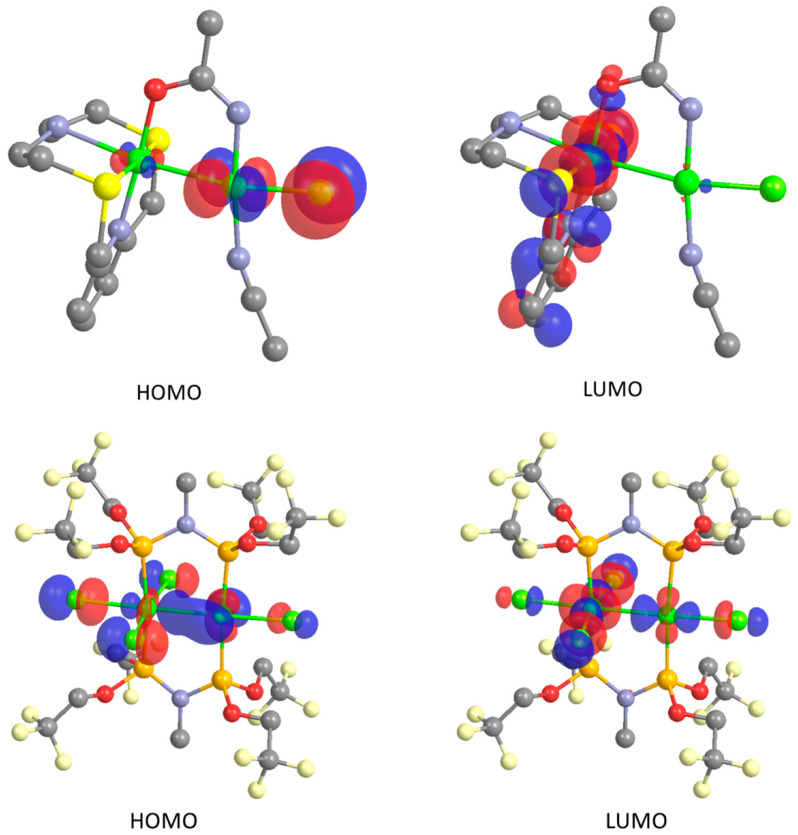
Frontier KS-MO isosurfaces calculated at the optimized geometry in its singlet ground-state for [Pt(**L^3^**)(μ-1,3-MeCONH)PtCl(MeCN)]^2+^ (top) and for [Pt_2_(tfepma)_2_Cl_4_] (bottom) in the gas phase; hydrogen atoms omitted for clarity; cutoff value = 0.05 |e|.

These data suggest that both complexes are better described as featuring closed-shell singlet Pt_2_ systems. Accordingly, the Kohn-Sham (KS) frontier molecular orbitals (MOs) are distributed between the two Pt ions in both complexes (Figure 12). Moreover, a natural population analysis (NPA) shows comparable natural charges on Pt*_O_* (*Q* = 0.047 and −0.862 |e|) and Pt*_SP_* (*Q* = 0.243 and −0.367 |e| for [Pt(**L^3^**)(μ-1,3-MeCONH)PtCl(MeCN)]^2+^ and [Pt_2_(tfepma)_2_Cl_4_], respectively). Consistently, very similar natural electron configurations were found in both complexes for the two Pt atoms, the electron populations differing by less than 0.5 |e|.

Finally, Wiberg (0.472 and 0.507) and Mayer bond indices (0.375 and 0.502 for [Pt(**L^3^**)(μ-1,3-MeCONH)PtCl(MeCN)]^2+^ and [Pt_2_(tfepma)_2_Cl_4_], respectively) for the Pt-Pt bond, suggest for both complexes a non-negligible covalent character.

## 3. Materials and Methods

All melting points are uncorrected. Elemental analyses were obtained using a Fison EA CHNS-O instrument operating at 1000 °C. FAB mass spectra were measured at the EPSRC National Mass Spectrometry Service at Swansea (UK). ^1^H and ^13^C NMR experiments were conducted at 25 °C with a Varian VXR400 spectrometer operating at 400 MHz for ^1^H and 100.62 MHz for ^13^C or with a Bruker Avance III HD spectrometer operating at 600 MHz for ^1^H and 150.9 MHz for ^13^C, using TMS as an internal standard. Data are reported as chemical shifts (multiplicity, coupling constants where applicable, number of hydrogen atoms, and assignment where possible). Abbreviations are: s (singlet), d (doublet), t (triplet), m (multiplet). Coupling constants (*J*) are quoted in Hertz (Hz) to the nearest 0.1 Hz. Absorption spectra were recorded with Varian Model Cary 5 UV-Vis-NIR spectrophotometer. Solvents and starting materials were purchased from commercial sources and used as received. Solvents used for the synthesis of the ligands were dried following conventional methods. The ligands 5-oxa-2,8-dithia[9](2,6)-pyridinophane (**L^1^**) and 2,5,8-trithia[9](2,6)-pyridinophane (**L^2^**), were synthesized according to reported procedures using as starting materials 2,6-dichloromethylpyridine and the dithiols O(HSCH_2_CH_2_)_2_ and S(HSCH_2_CH_2_)_2_ for **L^1^** and **L^2^**, respectively [22,33]. 2,8-Dithia-5-aza-2,6-pyridinophane (**L^3^**) was prepared according to the reported procedure starting from 2,6-dithiomethyl-pyridine and *N*-(*tert-*butoxycarbonyl)*bis*-(2-chloroethylamine) [13].

### 3.1. General Procedure for the Synthesis of the Pd^II^, Pt^II^, Rh^III^ Complexes of **L^1^**-**L^3^**

The appropriate metal chloride was added to a solution of **L**, ^1^**L^2^** or **L^3^** in 1:1 molar ratio. No excess of ligand or starting metal salt was considered, to avoid the formation of coordination compounds with stoichiometries other than 1:1, which is very likely when the macrocyclic ligand is not able to satisfy the stereo-electronic requirements of the metal ion. The reactions were all conducted in MeCN/H_2_O (20 mL, 1:1 *v*/*v*) solvent mixture, except in two cases where the MeOH/H_2_O (20 mL, 1:1 *v*/*v*) solvent mixture was used for solubility reasons, as specified below. The reaction mixture was refluxed under N_2_ for 5 h in all cases. When a pure solid product was not obtainable/isolable from the reaction mixture of the ligands with the chloride of the metal under investigation, a counter-anion metathesis reaction was performed to replace coordinating chlorido ligands with non-coordinating BF_4_^−^ or PF_6_^−^ anions and facilitate the crystallization or formation of solid products. This was necessary in the preparation of [Pt(**L^1^**)Cl](BF_4_), [Rh(**L^1^**)Cl_2_](PF_6_), [Rh(**L^2^**)Cl_2_](PF_6_), [Pd(**L^3^**)Cl](PF_6_), [Pt(**L^3^**)(μ-1,3-MeCONH)PtCl(MeCN)](BF_4_)_2_·H_2_O, [Rh(**L^3^**)Cl_2_](BF_4_)·MeCN for which a ten-fold molar excess NH_4_BF_4_ or NH_4_PF_6_ was added at room temperature after refluxing of the reaction mixture. This is a well-established synthetic procedure in the field of macrocyclic ligand chemistry [76,77].

[Pd(**L^1^**)Cl]_2_[Pd_2_Cl_6_]. To a solution of **L^1^** (0.051 g, 0.211 mmol) in MeOH/H_2_O (20 mL, 1:1 *v*/*v*) was added PdCl_2_ (0.037 g, 0.226 mmol). Reddish prismatic crystals (0.048 g, yield 19%) were obtained by reduction under the vacuum of the volume of the reaction mixture and subsequent crystallization in the air from the remaining aqueous solution by slow evaporation. Mp: 220 °C with decomposition. Elem. Anal. found (calc. for C_11_H_15_Cl_4_NOPd_2_S_2_): C, 22.4 (22.2); H, 2.6 (2.5) N, 2.5 (2.4); S, 10.6 (10.8). ^1^H-NMR (600 MHz, CD_3_CN): δ_H_ 2.74–2.78 (m, 2H, H_9a_/H_10a_ or H_9b_/H_10b_), 3.32–3.34 (m, 2H, H_8a_/H_11a_ or H_8b_/H_11b_), 3.54–3.59 (m, 2H, H_8b_/H_11b_ or H_8a_/H_11a_), 4.07–4.10 (m, 2H, H_9b_/H_10b_ or H_9a_/H_10a_), 4.45 (d, *J* = 18.5 Hz, 2H, H_7a_/H_12a_ or H_7b_/H_12b_), 4.88 (d, *J* = 18.5 Hz, 2H, H_7b_/H_12b_ or H_7a_/H_12a_), 7.53 (d, *J* = 7.9 Hz, 2H), 7.97 (t, *J* = 7.9 Hz, 1H). ^13^C-NMR (150.9 MHz, CD_3_CN): δ_C_ 45.0 (Ar*C*H_2_S), 45.9 (S*C*H_2_CH_2_O), 65.4 (SCH_2_*C*H_2_O), 122.8, 140.5, 164.1 (aromatic carbons). UV-Vis spectrum (H_2_O): λ (ε) 211 (2390), 261 (2190), 388 nm (190 dm^3^ mol^−1^cm^−1^). MS (FAB): *m/z* 419 ([C_11_H_15_Cl_2_NOPdS2]^+^).

[Pt(**L^1^**)Cl](BF_4_). To a solution of **L^1^** (0.035 g, 0.145 mmol) in MeOH/H_2_O (20 mL, 1:1 *v*/*v*) was added PtCl_2_ (0.038 g, 0.145 mmol). Yellow crystals (0.040 g, yield 49%) were obtained after the addition of NH_4_BF_4_ to the mixture, reduction under the vacuum of the volume of the reaction mixture and subsequent crystallization in the air from the remaining aqueous solution. Mp: 200 °C with decomposition. Elem. Anal. found (calc. for C_11_H_15_BClF_4_NOPtS_2_): C, 23.7 (23.6); H, 2.3 (2.7) N, 2.9 (2.5); S, 11.3 (11.5). ^1^H-NMR (600 MHz, CD_3_CN): δ_H_ 2.58–2.62 (m, 2H, H_9a_/H_10a_ or H_9b_/H_10b_), 3.48–3.61 (m, 4H, H_8a_/H_11a_ or H_8b_/H_11b_), 4.09–4.11 (m, 2H, H_9b_/H_10b_ or H_9a_/H_10a_), 4.62 (d, *J* = 18.0 Hz, 2H, H_7a_/H_12a_ or H_7b_/H_12b_), 4.85 (d, *J* = 18.0 Hz, 2H, H_7b_/H_12b_ or H_7a_/H_12a_), 7.61 (d, *J* = 6.0 Hz, 2H), 8.08 (t, *J* = 6.0 Hz, 1H). ^13^C-NMR (150.9 MHz, CD_3_CN): δ_C_ 46.7 (Ar*C*H_2_S), 47.4 (S*C*H_2_CH_2_O), 66.4 (SCH_2_*C*H_2_O), 123.3, 140.5, 163.4 (aromatic carbons). UV-Vis spectrum (H_2_O): λ (ε) 265 (2360), 360 nm (56 dm^3^ mol^−1^ cm^−1^). MS (FAB): *m/z* 472 ([C_11_H_15_ClNOPtS_2_]^+^).

[Rh(**L^1^**)Cl_2_](PF_6_). To a solution of **L^1^** (0.040 g, 0.166 mmol) in MeCN/H_2_O (20 mL, 1:1 *v*/*v*) was added RhCl_3_·H_2_O (0.038 g, 0.166 mmol). Yellow crystals (0.038 g, yield 41%) were obtained after the addition of NH_4_PF_6_ to the reaction mixture, evaporation under the vacuum of the solvent and subsequent crystallization of the solid obtained, by diffusion of Et_2_O vapor into an MeCN solution. Mp: 220 °C with decomposition. Elem. Anal. found (calc. for C_11_H_15_Cl_2_F_6_NOPRhS_2_): C, 24.0 (23.6); H, 2.9 (2.7); N, 2.9 (2.5); S, 11.3 (11.4)%. ^1^H-NMR (600 MHz, CD_3_CN): δ_H_ 3.36–3.40 (m, 2H, H_9a_/H_10a_ or H_9b_/H_10b_), 3.48–3.51 (m, 2H, H_8a_/H_11a_ or H_8b_/H_11b_), 3.57–3.62 (m, 2H, H_8b_/H_11b_ or H_8a_/H_11a_), 4.04–4.07 (m, 2H, H_9b_/H_10b_ or H_9a_/H_10a_), 5.01 (d, *J* = 18.6 Hz, 2H, H_7a_/H_12a_ or H_7b_/H_12b_), 5.25 (d, *J* = 18.6 Hz, 2H, H_7b_/H_12b_ or H_7a_/H_12a_), 7.77 (d, *J* = 8.0 Hz, 2H), 8.06 (t, *J* = 8.0 Hz, 1H). ^13^C-NMR (150.9 MHz, CD_3_CN): δ_C_ 40.5 (Ar*C*H_2_S), 46.0 (S*C*H_2_CH_2_O), 74.1 (SCH_2_*C*H_2_O), 124.2, 140.0, 162.2 (aromatic carbons). UV-Vis spectrum (MeCN): λ (ε) 322 (727), 371 (770), 424sh nm (544 dm^3^ mol^−1^ cm^−1^). MS (FAB): *m/z* 414 ([C_11_H_15_Cl_2_NORhS_2_]^+^).

[Pd(**L^2^**)Cl]Cl. To a solution of **L^2^** (0.058 g, 0.226 mmol) in MeCN/H_2_O (20 mL, 1:1 *v*/*v*) was added PdCl_2_ (0.040 g, 0.226 mmol). An orange microcrystalline solid (0.053 g, yield 54%) was obtained by removal under reduced pressure of solvent from the reaction mixture and subsequent crystallization of the crude product obtained by diffusion of Et_2_O vapor into a MeCN solution. Mp: 210 °C with decomposition. Elem. Anal. found (calc. for C_11_H_15_Cl_2_NPdS_3_): C, 30.3 (30.4); H, 3.3 (3.5) N, 3.3 (3.2); S, 22.6 (22.1)%. ^1^H-NMR (400 MHz, D_2_O): δ_H_ 2.13–2.20 (m, 4H, H_9a_/H_10a_), 3.56–3.78 (m, 4H, H_8a_/H_11a_), 4.70 (d, *J* = 18.8 Hz, 2H, H_7a_/H_12a_ or H_7b_/H_12b_), 5.17 (d, *J* = 18.4 Hz, 2H, H_7b_/H_12b_ or H_7a_/H_12a_), 7.69 (d, *J* = 8.0 Hz, 2H), 8.09 (m,1H). ^13^C-NMR (100.62 MHz, (CD_3_)_2_CO): δ_C_ 33.2 (SCH_2_*C*H_2_S), 46.9 (Ar*C*H_2_S), 48.0 (S*C*H_2_CH_2_S), 123.1, 141.1, 164.6 (aromatic carbons). UV-Vis spectrum (H_2_O): λ (ε) 265 (7000), 317 (200), 398 nm (80 dm^3^ mol^−1^cm^−1^). MS (FAB): *m/z* 433 ([C_11_H_15_Cl_2_NPdS_3_]^+^).

[Pt(**L^2^**)Cl]Cl. To a solution of **L^2^** (0.048 g, 0.186 mmol) in MeCN/H_2_O (20 mL, 1:1 *v*/*v*) was added PtCl_2_ (0.050 g, 0.186 mmol). A yellow solid (0.070 g, yield 78%) was obtained by evaporation under the vacuum of the reaction mixture and subsequent crystallization of the crude product obtained by diffusion of Et_2_O vapor into a MeCN solution. Mp: 215 °C with decomposition. Elem. Anal. found (calc. for C_11_H_15_Cl_2_NPtS_3_): C, 24.7 (25.2); H, 3.0 (2.9) N, 3.2 (2.7); S, 18.7 (18.4)%. ^1^H-NMR (600 MHz, CD_3_CN): δ_H_ 2.56–2.62 (m, 2H, H_9a_/H_10a_ or H_9b_/H_10b_), 2.67–2.72 (m, 2H, H_8a_/H_11a_ or H_8b_/H_11b_), 2.93–2.96 (m, 2H, H_8b_/H_11b_ or H_8a_/H_11a_), 3.28–3.32 (m, 2H, H_9b_/H_10b_ or H_9a_/H_10a_), 3.94 (d, *J* = 12.8 Hz, 2H, H_7a_/H_12a_ or H_7b_/H_12b_), 4.05 (d, *J* = 12.8 Hz, 2H, H_7b_/H_12b_ or H_7a_/H_12a_), 7.57 (d, *J* = 8.0 Hz, 2H), 7.83 (t, *J* = 8.0 Hz, 1H). ^13^C-NMR (150.9 MHz, CD_3_CN): δ_C_ 34.3 (SCH_2_*C*H_2_S), 39.2 (Ar*C*H_2_S), 39.8 (S*C*H_2_CH_2_S), 124.5, 138.6, 158.4 (aromatic carbons). UV-Vis spectrum (H_2_O): λ (ε) 265 (7500), 317 nm (120 dm^3^ mol^−1^cm^−1^). MS (FAB): *m/z* 488 ([C_11_H_15_ClNPtS_3_]^+^).

[Rh(**L^2^**)Cl_2_](PF_6_). To a solution of **L^2^** (0.040 g, 0.155 mmol) in MeCN/H_2_O (20 mL, 1:1 *v*/*v*) was added RhCl_3_·H_2_O (0.035 g, 0.155 mmol). A brown solid (0.034 g) was obtained after the addition of NH_4_PF_6_ to the mixture, evaporation of solvent from the reaction mixture under vacuum and subsequent crystallization in the air from the aqueous solution. Mp: 230 °C with decomposition. Elem. Anal. found (calc. for C_11_H_15_PCl_2_F_6_NRhS_3_): C, 22.4 (22.9); H, 2.3 (2.6); N, 3.0 (2.4); S, 16.1 (16.7)%. ^1^H-NMR (600 MHz, CD_3_CN): δ_H_ 2.59–2.65 (m, 2H), 2.73–2.79 (m, 2H), 3.31–3.36 (m, 2H), 3.46–3.51 (m, 2H), 3.57–3.63 (m, 2H), 3.86–3.94 (m, 4H), 4.03–4.06 (m, 2H), 4.81 (d, *J* = 18.2 Hz, 2H, H_7a_/H_12a_ or H_7b_/H_12b_), 4.87 (d, *J* = 18.3 Hz, 2H, H_7b_/H_12b_ or H_7a_/H_12a_), 5.14–5.18 (m, 4H), 7.69 (d, *J* = 7.9 Hz, 2H), 7.74 (d, *J* = 7.9 Hz, 2H), 8.02 (t, *J* = 7.9 Hz, 1H), 8.11 (t, *J* = 7.9 Hz, 1H) ^13^C-NMR (150.9 MHz, CD_3_CN): δ 36.2/36.6, 44.5/45.1, 45.4/46.8, 123.8/124.6, 139.9/141.2, 161.4/162.3. UV-Vis spectrum (MeCN): λ (ε) 325 (730), 365 (765), 421sh nm (540 dm^3^ mol^−1^cm^−1^). MS (FAB): *m/z* 430 ([C_11_H_15_Cl_2_NRhS_3_]^+^), 396 (C_11_H_15_ClNRhS_3_]^+^). One of the two sets of signals, in both the ^1^H- and ^13^C-NMR spectra belongs to the species [Rh(**L^2^**)Cl_2_](PF_6_), the other set belongs to a species having only one coordinated chlorido ligand (see discussion above). However, it is not possible to uniquely identify which set of signals corresponds to which complex.

[Pd(**L^3^**)Cl](PF_6_). A mixture of **L^3^** (0.020 g, 0.083 mmol) and PdCl_2_ (0.015 g, 0.083 mmol) in MeCN/H_2_O (20 mL, 1:1 *v*/*v*) was refluxed for 2 h. A brown solid (0.020 g, yield 46%) was obtained after the addition of excess NH_4_PF_6_ to the reaction mixture, reduction of the volume of the reaction mixture under vacuum, and subsequent crystallization in the air from the resulting aqueous solution by slow evaporation. Mp: 220 °C with decomposition. Elem. Anal. found (calc. for C_11_H_16_ClF_6_N_2_PPdS_2_): C, 25.4 (25.1); H, 2.8 (3.1); N, 5.7 (5.3); S, 12.6 (12.2)%. ^1^H-NMR (400 MHz, D_2_O): δ_H_ 3.03–3.06 (m, 4H, H_9a_/H_10a_), 3.54–3.60 (m, 4H, H_8a_/H_11a_), 4.61 (d, *J* = 18.6 Hz, 2H, H_7a_/H_12a_ or H_7b_/H_12b_), 5.07 (d, *J* = 18.0 Hz, 2H, H_7b_/H_12b_ or H_7a_/H_12a_), 7.65 (d, *J* = 8.1 Hz, 2H), 8.04 (m,1H). ^13^C-NMR (100.62 MHz, CD_3_CN): δ_C_ 43.3 (S*C*H_2_CH_2_N), 45.8 (Ar*C*H_2_S), 48.2 (SCH_2_*C*H_2_N), 122.1, 139.5, 163.6 (aromatic carbons). UV-Vis spectrum (MeCN): λ (ε) 274 (12480), 374 nm (3880 dm^3^ mol^−1^cm^−1^). MS (FAB): *m/z* 381 ([C_11_H_16_ClN_2_PdS_2_]^+^).

[Pt(**L^3^**)(μ-1,3-MeCONH)PtCl(MeCN)](BF_4_)_2_·H_2_O. To a solution of **L^3^** (0.020 g, 0.083 mmol) in MeCN/H_2_O (20 mL, 1:1 *v*/*v*) was added PtCl_2_ (0.022 g, 0.083 mmol), and the reaction mixture was refluxed for 2 h. Very few orange crystals were obtained after the addition of NH_4_BF_4_ to the mixture, reduction of the volume of the reaction mixture under vacuum, and subsequent crystallization in the air from the resulting aqueous solution by slow evaporation. Mp: 230 °C with decomposition. Elem. Anal. found (calc. for C_15_H_25_B_2_ClF_8_N_4_O_2_Pt_2_S_2_): C, 18.4 (18.8); H, 2.8 (2.6) N, 5.7 (5.9); S, 6.5 (6.7)%.

[Rh(**L^3^**)Cl_2_](BF_4_)·MeCN. To a solution of **L^3^** (0.020 g, 0.083 mmol) in MeCN/H_2_O (20 mL, 1:1 *v*/*v*) was added RhCl_3_·H_2_O (0.019 g, 0.083 mmol). Yellow crystals (0.020 g, yield 44%) were obtained after the addition of NH_4_BF_4_ to the mixture, reduction of the reaction mixture under vacuum and subsequent crystallization of the solid obtained by diffusion of Et_2_O vapors into a MeCN solution. Mp: 210 °C with decomposition. Elem. Anal. found (calc. for C_13_H_19_BCl_2_F_4_N_3_S_2_Rh): C, 28.7 (28.8); H, 3.2 (3.5); N, 7.6 (7.7); S, 12.2 (11.8)%. ^1^H-NMR (400 MHz, CD_3_CN): δ 2.39–2.48 (m, 2H, H_9a_/H_10a_ or H_9b_/H_10b_), 3.01–3.09 (m, 2H, H_8a_/H_11a_ or H_8b_/H_11b_), 3.33–3.42 (m, 2H, H_8b_/H_11b_ or H_8a_/H_11a_), 3.55–3.62 (m, 2H, H_9b_/H_10b_ or H_9a_/H_10a_), 4.93 (d, *J* = 18.4 Hz, 2H, H_7a_/H_12a_ or H_7b_/H_12b_), 5.21 (d, *J* = 18.4 Hz, 2H, H_7b_/H_12b_ or H_7a_/H_12a_), 7.71 (d, *J* = 8.4 Hz, 2H), 8.04 (t, *J* = 8.0 Hz, 1H). ^13^C-NMR (100.62 MHz, CD_3_CN): δ 42.6 (S*C*H_2_CH_2_N), 46.6 8 (Ar*C*H_2_S), 52.6 (SCH_2_*C*H_2_N), 124.4, 140.2, 162.7 (aromatic carbons). UV-Vis spectrum (MeCN): λ (ε) 274 (3110), 380 nm (933 dm^3^ mol^−1^cm^−1^). MS (FAB): *m/z* 413 ([C_11_H_16_Cl_2_N_2_RhS_2_]^+^).

### 3.2. Theoretical Calculations

Theoretical calculations were performed on the complex cation [Pt(**L^3^**)(μ-1,3-MeCONH)PtCl(MeCN)]^2+^ and on [Pt_2_(tfepma)_2_Cl_4_] (tfepma = ((CF_3_CH_2_O)_2_P)_2_NCH_3_) [46,47] at the density functional theory (DFT) [56] level with the Gaussian 16 (Rev. B.01) suite of programs [78], on a IBM x3755 server with four 12-core processors and 64 Gb of RAM (OS: SUSE Linux Enterprise Server 11 SP3). The mPW1PW functional [71] was adopted, in combination with the full-electron split valence basis sets (BSs) def2-SVP [72,73] for light atoms and the LANL08(f) BS [74], including *f* polarization functions for the outer electron shell and Relativistic Effective Core Potentials (RECPs) [75], for the Pt atomic species. All basis sets and RECPs were obtained from Basis Set Exchange and Basis Set EMSL Library [79].

The geometries of all compounds were optimized starting from crystal structure data in their triplet ground-state (2*S* + 1 = 3, two unpaired electrons), closed-shell singlet state (2*S* + 1 = 1) after verification of the wavefunction stability (*stable* = *opt*), or by means of a broken-symmetry (DFT-BS) approach. The procedure recently developed for bis(1,2-dithiolene) metal complexes was followed [70]. In particular, the BS electron density guess was obtained through a fragmented approach (*guess* = *fragment* = *n*, the fragments being the two Pt ions and the various ligands) starting from the geometry optimized at the largest spin multiplicity, by attributing different combinations of charges (*Q* = +1, +2, +3) and corresponding spin multiplicities (2*S* + 1 = 1, 2, 3) to the Pt ions, eventually optimizing (*opt*) the geometry of the complexes for the different combinations and verifying (and in case re-optimizing) the stability of the wavefunctions [70]. Fine numerical integration grids (*Integral = ultrafine* keyword) were used, and the nature of the minima of each optimized structure was verified by harmonic frequency calculations (*freq = raman* keyword). A natural population analysis was carried out at the optimized geometries using the natural bonding orbital (NBO) partitioning scheme [80]. The programs GaussView 6.0.16 [81], Molden 6.6 [82], and Chemissian 4.53 [83] were used to investigate the optimized structures and the shapes of Kohn–Sham molecular orbitals.

### 3.3. X-ray Crystallography

A summary of the crystal data and refinement details for the compounds discussed in this paper is given in Tables S7 and S8 (SM). Diffraction data for [Pd(**L^1^**)Cl]_2_[Pd_2_Cl_6_] and [Pt(**L^3^**)(μ-1,3-MeCONH)PtCl(MeCN)](BF_4_)_2_·H_2_O were collected at 293(2) K as *ω* scans on an APEX II CCD Diffractometer. Diffraction data for [Rh(**L^1^**)Cl_2_](PF_6_) were collected at 294(2) K as *ω* scans on an ENRAF NONIUS CAD4 Diffractometer. For [Pt(**L^1^**)Cl](BF_4_) and [Rh(**L^3^**)Cl_2_](BF_4_)·MeCN diffraction data were collected at 150(2) K as *ω* scans on, respectively, a Bruker SMART1000 CCD Area Detector Diffractometer and a Bruker SMART-APEX CCD Area Detector Diffractometer equipped with an Oxford Cryosystem open-flow cryostat. Data were collected using graphite-monochromated MoKα radiation (λ = 0.71073 Å). Absorption corrections were treated by semiempirical corrections based on multiple scans, as specified in the CIF files.

All the structures were solved by direct methods using *SIR*92 [84] ([Pd(**L^1^**)Cl]_2_[Pd_2_Cl_6_], [Rh(**L^1^**)Cl_2_](PF_6_), [Pt(**L^3^**)(μ-1,3-MeCONH)PtCl(MeCN)](BF_4_)_2_·H_2_O, [Rh(**L^3^**)Cl_2_](BF_4_)·MeCN) and *SHELXS-*97 [85] ([Pt(**L^1^**)Cl](BF_4_)) and completed by iterative cycles of full-matrix least-squares refinement and Δ*F* syntheses using the software package *SHELXL* [85]. For [Rh(**L^3^**)Cl_2_](BF_4_)·MeCN diffraction was poor in certain directions; twinning was modeled as non-merohedral by 180° rotation about the [100] axis with a twin fraction of 0.0463(1). In all cases, non-H atoms were refined with anisotropic displacement parameters, while H atoms were introduced at calculated positions and refined using a riding model. In [Pt(**L^3^**)(μ-1,3-MeCONH)PtCl(MeCN)](BF_4_)_2_·H_2_O and [Rh(**L^3^**)Cl_2_](BF_4_)·MeCN the N-H hydrogen atom on the amidate ligand and the solvent methyl H-atoms, respectively, were found in a difference Fourier map and thereafter refined using a riding model.

## 4. Conclusions

In this paper, the coordination chemistry of the mixed-donor tetradentate macrocycles **L^1^**-**L^3^** featuring a pyridine moiety towards platinum group metal ions Pd^II^, Pt^II^, and Rh^III^ has been investigated. In all isolated 1:1 metal-to-ligand complexes, the ligands adopt a folded conformation and impose a [3 + 1] coordination mode at the Pd^II^ and Pt^II^ metal centers within a distorted square-based coordination sphere. In the case of Rh^III^ complexes, the tetradentate ligands occupy four of the six positions of a distorted octahedral geometry with the other two coordination sites in a relative *cis* orientation occupied by two Cl^−^ ligands. A rare example of a discrete Pt_2_ dimer was isolated by serendipity from the reaction of **L^3^** and PtCl_2_ in refluxing MeCN/H_2_O (1:1 *v*/*v*), and structurally characterized. This complex, based on data from the literature, could have been formally defined as a *d*^9^-*d*^7^ Pt_2_^I,III^ mixed-valence binuclear complex featuring a Pt-Pt bond linking a square-planar and an octahedral platinum centers. DFT calculations, following the broken symmetry approach (DFT-BS), identify a singlet ground-state nature (*d*^8^-*d*^8^ Pt^II^-Pt^II^) both for the isolated compound, as well as for the only other example of a discrete binuclear Pt_2_ complex of the same type reported in the literature, despite the different coordination environments of the two metal centers typical for a *d*^8^ Pt^II^ center (square-planar) and a *d*^7^ Pt^III^ center (octahedral). Notwithstanding the theoretical limits inherent to a non-multireference DFT-BS approach, the case of [Pt(**L^3^**)(μ-1,3-MeCONH)PtCl(MeCN)]^+^ suggests that a more in-depth re-evaluation may be needed for the electronic configurations assigning mixed oxidation states to Pt ions in dinuclear complexes where two directly interacting Pt ions show different coordination geometries and numbers.

The obtained results, especially in the case of **L^3^**, can be of help in understanding the sensing properties toward metal ions of fluorescent chemosensors featuring this macrocycle as receptor units [13,14,15,16,17,18,19,20,21]. However, as far as the question posed in the title is concerned, based on the results obtained, we can conclude that well-established fields of coordination chemistry, such as that of macrocyclic ligands and Pt^II^, can still hold some unexpected outcomes. The serendipitous and unexpected isolation of complex [Pt(**L^3^**)(η-1,3-MeCONH)PtCl(MeCN)](BF_4_)_2_·H_2_O stands as a proof of principle for the unexplored synthetic possibilities still available in the coordination chemistry of well-known classes of macrocyclic ligands and Platinum Group metals. We have shown that the dimeric and unique complex cation [Pt(**L^3^**)(μ-1,3-MeCONH)PtCl(MeCN)]^+^ can exist, despite the fact we were not able to reproduce it or explain its formation. This is still interesting and could open new perspectives in the coordination compounds of Pt^II^. Furthermore, we strongly believe that it is essential to perform fundamental research even when all the available information suggests that only predictable, trivial results will be obtained.

## Data Availability

CCDC 2054238–2054242 contains the supplementary crystallographic data for this paper. These data can be obtained free of charge from the Cambridge Crystallographic Data Centre via www.ccdc.cam.ac.uk/data_request/cif (accessed on 8 January 2021).

## Supplementary Materials

The following are available online: FAB Mass Spectra, ^1^H- and ^13^C-NMR spectra of the isolated complexes. Details of the crystal packing of [Pd(**L^1^**)Cl]_2_[Pd_2_Cl_6_], [Pt(**L^1^**)Cl](BF_4_), [Rh(**L^1^**)Cl_2_](PF_6_), and [Pt(**L^3^**)(μ-1,3-MeCONH)PtCl(MeCN)](BF_4_)_2_·H_2_O. Discussion on the solution structures of the of **L^1^** and **L^2^** complexes with Rh^III^. Molecular drawings for the complex cation [Pt(**L^3^**)(μ-1,3 MeCONH)PtCl(MeCN)]^2+^ and the complex [Pt_2_(tfepma)_2_Cl_4_] at the optimized geometry in their singlet and triplet states in the gas phase. Optimized geometry in orthogonal Cartesian coordinate format calculated for [Pt(**L^3^**)(μ-1,3-MeCONH)PtCl(MeCN)]^2+^ and [Pt_2_(tfepma)_2_Cl_4_] in their singlet and triplet states. Selected optimized bond lengths (Å) and angles (°) calculated in the gas phase for [Pt(**L^3^**)(μ-1,3-MeCONH)PtCl(MeCN)]^2+^ and [Pt_2_(tfepma)_2_Cl_4_] at the optimized geometry in their singlet and triplet states, and corresponding crystal structure data. Details of X-ray data collection and refinement for [Pd(**L^1^**)Cl]_2_[Pd_2_Cl_6_], [Pt(**L^1^**)Cl](BF_4_), [Rh(**L^1^**)Cl_2_](PF_6_), [Pt(**L^3^**)(μ-1,3-MeCONH)PtCl(MeCN)](BF_4_)_2_·H_2_O and [Rh(**L^3^**)Cl_2_](BF_4_)·MeCN.

## Sample Availability

Samples of all compounds except [Pt(**L^3^**)(μ-1,3-MeCONH)PtCl(MeCN)](BF_4_)_2_·H_2_O are available from the authors.

## References

[1] Gloe K. (2005). Macrocyclic Chemistry, Current Trends and Future Perspectives.

[2] Fitzpatrick D.W., Ulrich H.J. (2010). Macrocyclic Chemistry: New Research Developments (Chemistry Research and Applications).

[3] Zolotov Y.A. (1997). Macrocyclic Compounds in Analytical Chemistry.

[4] Davis F., Higson S. (2011). Macrocycles: Construction, Chemistry and Nanotechnology Applications.

[5] Marsault E., Peterson M.L. (2017). Practical Medicinal Chemistry with Macrocycles: Design, Synthesis, and Case Studies.

[6] Levin E.I. (2014). Macrocycles in Drug Discovery.

[7] Gaeta C., Wang D.-X. (2020). New Macrocycles and Their Supramolecular Perspectives.

[8] Prodi L., Bolletta F., Montaldi M., Zaccheroni N. (2000). Luminescent chemosensors for transition metal ions. Coord. Chem. Rev..

[9] Balzani V., Credi A., Venturi M. (2003). Molecular Devices and Machines, A Journey into the Nanoworld.

[10] Formica M., Fusi V., Giorgi L., Micheloni M. (2012). New fluorescent chemosensors for metal ions in solution. Coord. Chem. Rev..

[11] Lindoy L.F. (1989). The Chemistry of Macrocyclic Ligands Complexes.

[12] Cronin L. (2005). Macrocyclic coordination chemistry. Annu. Rep. Prog. Chem. Sect. A Inorg. Chem..

[13] Blake A.J., Bencini A., Caltagirone C., De Filippo G., Dolci L.S., Garau A., Isaia F., Lippolis V., Mariani P., Prodi L. (2004). A new pyridine-based 12-membered macrocycle functionalised with different fluorescent subunits: Coordination chemistry towards Cu^II^, Zn^II^, Cd^II^, Hg^II^, and Pb^II^. Dalton Trans..

[14] Aragoni M.C., Arca M., Bencini A., Blake A.J., Caltagirone C., De Filippo G., Devillanova F.A., Garau A., Gelbrich T., Hursthouse M.B. (2007). Tuning the selectivity/specificity of fluorescent metal ion sensors based on N_2_S_2_ pyridine-containing macrocyclic ligands by changing the fluorogenic sub-unit: Spectrofluorimetric and metal ion binding studies. Inorg. Chem..

[15] Shamsipur M., Sadeghi M., Alizadeh K., Bencini A., Valtancoli B., Garau A., Lippolis V. (2010). Novel fluorimetric bulk optode membrane based on 5,8-bis(5’-chloro-8’-hydroxy-7’-quinolinyl)methyl)-2,11-dithia-5,8-diaza-2,6-pyridinophane for selective detection of lead(II) ions. Talanta.

[16] Shamsipur M., Zahedi M.M., De Filippo G., Lippolis V. (2011). Development of a novel flow injection liquid-liquid microextraction method for on-line separation, preconcentration and fluorimeteric determination of zinc(II) using 5-(8-hydroxy-2-quinolinylmethyl)-2,8-dithia-5-aza-2,6-pyridinophane as a sensitive and selective fluorescent chemosensor. Talanta.

[17] Shamsipur M., Sadeghi M., Garau A., Lippolis V. (2013). An efficient and selective flourescent chemical sensor based on 5-(8-hydroxy-2-quinolinylmethyl)-2,8-dithia-5-aza-2,6-pyridinophane as a new fluoroionophore for determination of iron(III) ions. A novel probe for iron speciation. Anal. Chim. Acta.

[18] Aragoni M.C., Arca M., Bencini A., Caltagirone C., Garau A., Isaia F., Light M.E., Lippolis V., Lodeiro C., Mameli M. (2013). Zn^2+^/Cd^2+^ optical discrimination by fluorescent chemosensors based on 8-hydroxyquinoline derivatives and sulfur-containing macrocyclic units. Dalton Trans..

[19] Bazzicalupi C., Caltagirone C., Cao Z., Chen Q., Di Natale C., Garau A., Lippolis V., Lvova L., Liu H., Lundström I. (2013). Multimodal use of new coumarin-based fluorescent chemosensors: Towards highly selective optical sensors for Hg^2+^ probing. Chem. A Eur. J..

[20] Arca M., Caltagirone C., De Filippo G., Formica M., Fusi V., Giorgi L., Lippolis V., Prodi L., Rampazzo E., Scorciapino M.A. (2014). A fluorescent ratiometric nanosized system for the determination of Pd^II^ in water. Chem. Commun..

[21] Lvova L., Caroleo F., Garau A., Lippolis V., Giorgi L., Fusi V., Zaccheroni N., Lombardo M., Prodi L., Di Natale C. (2018). A fluorescent sensor array based on heteroatomic macrocyclic fluorophores for the detection of polluting species in natural water samples. Front. Chem. Sect. Anal. Chem..

[22] Blake A.J., Demartin F., Devillanova F.A., Garau A., Isaia F., Lippolis V., Schröder M., Verani G. (1996). A new class of mixed aza-thioether crown containing a 1,10-phenanthroline sub-unit. J. Chem. Soc. Dalton Trans..

[23] Aragoni M.C., Arca M., Demartin F., Devillanova F.A., Isaia F., Garau A., Lippolis V., Jalali F., Papke U., Shamsipur M. (2002). Fluorometric Chemosensors. Interaction of toxic heavy metal ions Pb^II^, Cd^II^, and Hg^II^ with novel mixed-donor phenanthroline-containing macrocycles: Spectrofluorometric, conductometric, and crystallographic studies. Inorg. Chem..

[24] Casula A., Nairi V., Fernández-Moreira V., Laguna A., Lippolis V., Garau A., Gimeno M.C. (2015). Re(I) derivatives functionalised with thioether crowns containing the 1,10-phenanthroline subunit as a new class of chemosensors. Dalton Trans..

[25] Aragoni M.C., Arca M., Bencini A., Biagini S., Blake A.J., Caltagirone C., Demartin F., De Filippo G., Devillanova F.A., Garau A. (2008). Interaction of mixed-donor macrocycles containing the 1,10-phenanthroline subunit with selected transition and post-transition metal ions: Metal ion recognition in competitive liquid-liquid solvent extraction of Cu^II^, Zn^II^, Pb^II^, Cd^II^, Ag^I^, and Hg^II^. Inorg. Chem..

[26] Shamsipur M., Javanbakht M., Mousavi M.F., Ganjali M.R., Lippolis V., Garau A., Tei L. (2001). Copper(II)-selective membrane electrodes based on some recently synthesized mixed aza-thioether crowns containing a 1,10-phenanthroline sub-unit. Talanta.

[27] Shamsipur M., Javanbakht M., Lippolis V., Garau A., De Filippo G., Ganjali M.R., Yari A. (2002). Novel Ag+ ion-selective electrodes based on two new mixed azathioether crowns containing a 1,10-phenanthroline sub-unit. Anal. Chim. Acta.

[28] Shamsipur M., Javanbakht M., Ganjali M.R., Mousavi M.F., Lippolis V., Garau A. (2002). Mixed aza-thioether crowns containing a 1,10-phenanthroline sub-unit as neutral ionophores for silver ion. Electroanalysis.

[29] Shamsipur M., Kazemi S.Y., Azimi G., Madaeni S.S., Lippolis V., Garau A., Isaia F. (2003). Selective transport of silver ion through a supported liquid membrane using some mixed aza-thioether crowns containing a 1,10-phenanthroline sub-unit as specific ion carriers. J. Membr. Sc..

[30] Shamsipur M., Hashemi O.R., Lippolis V. (2006). A supported liquid membrane system for simultaneous separation of silver(I) and mercury(II) from dilute feed solutions. J. Membr. Sc..

[31] Shamsipur M., Hashemi B., Dehdashtian S., Mohammadi M., Gholivand M.B., Garau A., Lippolis V. (2014). Silver ion imprinted polymer nanobeads based on a aza-thioether crown containing a 1,10-phenanthroline subunit for solid phase extraction and for voltammetric and potentiometric silver sensors. Anal. Chim. Acta.

[32] Contu F., Demartin F., Devillanova F.A., Garau A., Isaia F., Lippolis V., Salis A., Verani G. (1997). Conformationally locked mixed aza-thioether macrocycles: Synthesis and structures of complexes of Pd^II^, Pt^II^ and Rh^III^ of 2,5,8-trithia-[9](2,9)-1,10-phenanthrolinophane. J. Chem. Soc. Dalton Trans..

[33] Arca M., Blake A.J., Casabò J., Demartin F., Devillanova F.A., Garau A., Isaia F., Lippolis V., Kivekas R., Muns V. (2001). Conformationally locked pentadentate macrocycles containing the 1,10-phenanthroline unit. Synthesis and crystal structure of 5-oxa-2,8-dithia[9](2,9)-1,10-phenanthrolinophane (L) and its coordination properties to Ni^II^, Pd^II^, Pt^II^, Rh^III^ and Ru^II^. J. Chem. Soc. Dalton Trans..

[34] Casabo J., Escriche L., Alegret S., Jaime C., Perez-Jimenez C., Ruis J., Molins E., Miravitlles C., Teixidor F., Mestres L. (1991). Pyridine-based macrocycles containing N, O, and S and their use as ion-selective electrodes. Crystal structures of 15-aza-6-oxa-3,9-dithiabicyclo[9.3.1]pentadeca-1(15),11,13-triene and (15-aza-6-oxa-3,9-dithiabicyclo[9.3.1]pentadeca-1(15),11,13-triene)dichlorocopper(II). Inorg. Chem..

[35] Rasheed O.K., Bawn C., Davies D., Raftery J., Victorica-Yrzebal I., Pritchard R., Zhou H., Quayle P. (2017). The Synthesis of group 10 and 11 metal complexes of 3,6,9-trithia-1-(2,6)-pyridinacyclodecaphane and their use in A^3^-coupling reactions. Eur. J. Org. Chem..

[36] Reddy P.J., Ravichandran V., Chacko K.K. (1989). Structure of the 2,5,8-trithia[9](2,6)pyridinophane-silver nitrate complex (1:1). Acta Cryst..

[37] Sobhia M.E., Panneerselvam K., Chacko K.K. (1992). Crystal structure of the 2:1 complex of mercury(II) chloride with trithiapyridino-12-crown-4 having unusual mercury coordination. Inorg. Chem. Acta.

[38] Blake A.J., Caçote M.H.M., Devillanova F.A., Garau A., Isaia F., Lippolis V., Pereira C.M., Silva F., Tei L. (2002). Coordination Chemistry of 2,5,8-Trithia[9],(2,9)-1,10-phenanthrolinophane (L) toward Rhodium(III) at the Polarised Water/1,2-Dichloroethane Interface—A Possible New Approach to the Problem of Separating Rh^III^ from Chloride Media. Eur. J. Inorg. Chem..

[39] Weber G., Jones P.G., Sheldrick G.M. (1983). 2,5,8-Trithia[9](2,6)-pyridinophane, C_11_H_15_NS_3_. Acta Cryst. C.

[40] Huheey J.E., Keiter E.A., Keiter R.L. (1993). Inorganic Chemistry, Principles of Structures and Reactivity.

[41] Lous R., Pelissard D., Weiss R. (1974). Complexes métalliques avec des ligands macrocycliques pentadentates. Structure cristalline et moléculaire du complexe [Pd(C_10_H_22_H_2_OS_2_)] (NO_3_)_2_. Acta Crystallogr..

[42] Lucas C.R., Liang W., Miller D.O., Bridson J.N. (1997). Metal complexes of 1-oxa-4,7-dithiacyclononane. Inorg. Chem..

[43] Concolino T.E., Eglin J.L., Staples R.J. (1999). Structural and spectroscopic characterization of the dirhenium acetamidate products resulting from the hydrolysis of acetonitrile. Polyhedron.

[44] Shishilov O.N., Akhmadullina N.S., Rezinkova Y.N., Podobedov R.E., Churakov A.V., Efimenko I.A. (2013). Reactivity of polynuclear palladium carboxylate complexes towards acetonitrile: Synthesis and X-ray study of Pd_2_(C_6_H_4_-o-C(=NH)CH_3_)_2_(CH_3_CO_2_)_2_ and Pd_5_(CH_3_C(=N)OC(=N)CH_3_)(NO)(NO_2_)x(RCO_2_)_7−x_. Dalton Trans..

[45] Adrian R.A., Zhu S., Powell D.R., Broker G.A., Tiekink E.R.T., Walmsley J.A. (2007). Dinuclear palladium(II) complexes with bridging amidate ligands. Dalton Trans..

[46] Cook T.R., Surendranath Y., Nocera D.G. (2009). Chlorine Photoelimination from a Diplatinum Core: Circunventing the Back Reaction. J. Am. Chem. Soc..

[47] Powers D.C., Hwang S.J., Anderson B.L., Yang H., Zheng S.-L., Chen Y.-S., Cook T.R., Gabbaï F.P., Nocera D.G. (2016). Stereoelectronic effects in Cl2 elimination from binuclear Pt(II) complexes. Inorg. Chem..

[48] Number of Structurally Independent Fragments Found for Discrete Structures of Binuclear Complexes Containing a Pt-Pt Bond: Pt4-Pt4: 53; Pt4-Pt5: 23; Pt4-Pt6: 2; Pt5-Pt5: 226; Pt5-Pt6: 43, Pt6-Pt6: 155; Pt7-Pt7: 3 (see Figure 9; ConQuest v.2020.2, CSD release, CCDC 2020). https://www.ccdc.cam.ac.uk/.

[49] Appleton T.G., Barnham K.J., Byriel K.A., Hall J.R., Kennard C.H.L., Mathieson M.T., Penman K.G. (1995). Reactions of nitroplatinum complexes. 2. Reactions of K_2_[PtNO_4_]_2_ and related complexes with aqueous acids (CH_3_CO_2_H, HClO_4_, CF_3_SO_3_H, HNO_3_, and H_2_SO_4_): Pathways to platinum(III) complexes with acetate bridges. Crystal Structure of K_2_[{Pt(NO_2_)_2_(μ-CH_3_CO_2_)}_2_]H_2_O. Inorg. Chem..

[50] Uson R., Fornies J., Tomas M., Casas J.M., Cotton F.A., Falvello L.R., Feng X. (1993). Synthesis and structural characterization of the Pt_2_(II,III) complex (NBu_4_)[(C_6_F_5_)_2_Pt(μ-C_6_F_5_Cl)Pt(C_6_F_5_)_2_] and the Pt_2_(III,III) Complex (NBu_4_)[(C_6_F_5_)_2_Pt(μ-C_6_F_5_Cl)(μ-C_6_F_5_)Pt(C_6_F_5_)_2_]. Ligand Reactivity of a Bridging C_6_F_5_ Group. J. Am. Chem. Soc..

[51] Bennett M.A., Bhargava S.K., Boas J.F., Boere R.T., Bond A.M., Edwards A.J., Guo S.-X., Hammerl A., Pilbrow J.R., Priver S.H. (2005). Electrochemically informed synthesis and characterization of salts of the [Pt_2_(μ-κAs,κ*C*-C_6_H_3_-5-Me-2-AsPh_2_)_4_]^+^ lantern complex containing a Pt−Pt Bond of Order ½. Inorg. Chem..

[52] Canty A.J., Gardiner M.G., Jones R.C., Rodemann T., Sharma M. (2009). Binuclear intermediates in oxidation reactions: [(Me_3_SiC≡C)Me_2_(bipy)Pt−PtMe_2_(bipy)]^+^ in the oxidation of Pt^II^Me_2_(bipy) (bipy = 2,2′-bipyridine) by IPh(C≡CSiMe_3_)(OTf) (OTf = Triflate). J. Am. Chem. Soc..

[53] Luedtke A.T., Goldberg K.I. (2007). Reductive elimination of ethane from five-coordinate Platinum(IV) alkyl complexes. Inorg. Chem..

[54] Pham D.M., Rios D., Olmstead M.M., Balch A.L. (2005). Assisted self-association of dicyanoaurate, [Au(CN)_2_]^−^, and dicyanoargentate, [Ag(CN)_2_]^−^, through hydrogen bonding to metal ammonia complexes. Inorg. Chim. Acta.

[55] Vicente J., Arcas A., Fernandez-Hernandez J.M., Sironi A., Masciocchi N. (2005). An unprecedented process involving normal and redox transmetallation reactions between Hg and Pt affording the unexpected K[Pt_2_{CH_2_C(O)Me}_6_(μ-Cl)_3_] complex: The key role of X-ray powder diffraction in unravelling its nature and structure. Chem. Commun..

[56] Koch W., Holthausen M.C. (2001). A Chemist’s Guide to Density Functional Theory.

[57] Pintus A., Aragoni M.C., Bellec N., Devillanova F.A., Lorcy D., Isaia F., Lippolis V., Randall R.A.M., Roisnel T., Slawin A.M.Z. (2012). Structure-property relationships in Pt^II^ diimine-dithiolate nonlinear optical chromophores based on arylethylene-1,2-dithiolate and 2-thioxothiazoline-4,5-dithiolate. Eur. J. Inorg. Chem..

[58] Maiore L., Aragoni M.C., Deiana C., Cinellu M.A., Isaia F., Lippolis V., Pintus A., Serratrice M., Arca M. (2014). Structure-activity relationships in cytotoxic Au^I^/Au^III^ complexes derived from 2-(2′-pyridyl)benzimidazole. Inorg. Chem..

[59] Cinellu M.A., Arca M., Ortu F., Stoccoro S., Zucca A., Pintus A., Maiore L. (2019). Structural, theoretical and spectroscopic characterisation of a series of novel gold (I)-norbornene complexes supported by phenanthrolines: Effects of the supporting ligand. Eur. J. Inorg. Chem..

[60] Pintus A., Aragoni M.C., Cinellu M.A., Maiore L., Isaia F., Lippolis V., Orrù G., Tuveri E., Zucca A., Arca M. (2017). [Au(pyb-H)(mnt)]: A novel gold(III) 1,2-dithiolene cyclometalated complex with antimicrobial activity (pyb-H = C-deprotonated 2-benzylpyridine; mnt = 1,2-dicyanoethene-1,2-dithiolate). J. Inorg. Biochem..

[61] Romanova J., Ranga Prabhath M.R., Jarowski P.D. (2016). Relationship between metallophilic interactions and luminescent properties in Pt(II) complexes: TD-DFT guide for the molecular design of light-responsive materials. J. Phys. Chem. C.

[62] Huang S., Yang B., Zhong J., Zhang H. (2015). A theoretical investigation on the metal–metal interaction in a series of pyrazolate bridged platinum(II) complexes. Synth. Met..

[63] Novikov A.S. (2018). Strong metallophilic interactions in nickel coordination compounds. Inorg. Chim. Acta.

[64] Blake A.J., Donamaría R., Lippolis V., López-de-Luzuriaga J.M., Monge M., Olmos M.E., Seal A., Weinstein J.A. (2019). Unequivocal experimental evidence of the relationship between emission energies and aurophilic interactions. Inorg. Chem..

[65] Noodleman L. (1981). Valence bond description of antiferromagnetic coupling in transition metal dimers. J. Chem. Phys..

[66] Noodleman L., Davidson E.R. (1986). Ligand spin polarization and antiferromagnetic coupling in transition metal dimers. Chem. Phys..

[67] Noodleman L., Case D.A. (1992). Density-functional theory of spin polarization and spin coupling in iron-sulfur clusters. Adv. Inorg. Chem.

[68] Onofrio N., Mouesca J.M. (2011). Analysis of the singlet-triplet splitting computed by the density functional theory-broken-symmetry method: Is it an exchange coupling constant?. Inorg. Chem..

[69] Pintus A., Ambrosio L., Aragoni M.C., Binda M., Coles S.J., Hursthouse M.B., Isaia F., Lippolis V., Meloni G., Natali D. (2020). Photoconducting devices with response in the Visible–Near-Infrared Region based on neutral Ni complexes of aryl-1,2-dithiolene ligands. Inorg. Chem..

[70] Aragoni M.C., Caltagirone C., Lippolis V., Podda E., Slawin A.M.Z., Woollins J.D., Pintus A., Arca M. (2020). Diradical character of neutral heteroleptic bis(1,2-dithiolene) metal complexes: Case study of [Pd(Me_2_timdt)(mnt)] (Me_2_timdt = 1,3-dimethyl-2,4,5-trithioxoimidazolidine; mnt^2–^ = 1,2-dicyano-1,2-ethylenedithiolate). Inorg. Chem..

[71] Adamo C., Barone V. (1998). Exchange functionals with improved long-range behavior and adiabatic connection methods without adjustable parameters: The mPW and mPW1PW models. J. Chem. Phys..

[72] Schäfer A., Horn H., Ahlrichs R. (1992). Fully optimized contracted Gaussian basis sets for atoms Li to Kr. J. Chem. Phys..

[73] Weigend F., Ahlrichs R. (2005). Balanced basis sets of split valence, triple zeta valence and quadruple zeta valence quality for H to Rn: Design and assessment of accuracy. Phys. Chem. Chem. Phys..

[74] Roy L.E., Hay P.G., Martin R.L. (2008). Revised Basis Sets for the LANL Effective Core Potentials. J. Chem. Theory Comput..

[75] Ortiz J.V., Hay P.J., Martin R.L. (1992). Role of d and f orbitals in the geometries of low-valent actinide compounds. Ab initio studies of U(CH_3_)_3_, Np(CH_3_)_3_, and Pu(CH_3_)_3_. J. Am. Chem. Soc..

[76] Danks J.P., Champness N.R., Schröder M. (1998). Chemistry of mixed nitrogen- and sulfur-donor tridentate macrocycles. Coord. Chem. Rev..

[77] Blake A.J., Schröder M. (1990). Chemistry of thioether macrocyclic complexes. Adv. Inorg. Chem..

[78] Frisch M.J., Trucks G.W., Schlegel H.B., Scuseria G.E., Robb M.A., Cheeseman J.R., Scalmani G., Barone V., Petersson G.A., Nakatsuji H. (2016). Gaussian 16, Rev. B.01.

[79] Schuchardt K.L., Didier B.T., Elsethagen T., Sun L., Gurumoorthi V., Chase J., Li J., Windus T.L. (2007). Basis set exchange: A community database for computational sciences. J. Chem. Inf. Model..

[80] Reed A.E., Weinstock R.B., Weinhold F. (1985). Natural population analysis. J. Chem. Phys..

[81] Dennington R.D., Keith T.A., Millam J.M. (2016). GaussView 6.0. 16.

[82] Schaftenaar G., Noordik J.H. (2000). Molden: A pre- and post-processing program for molecular and electronic structures. J. Comput. Aided. Mol. Des..

[83] Skripnikov L.V. (2017). Chemissian Version 4.53.

[84] Altomare G., Cascarano G., Giacovazzo C., Guagliardi A., Burla M., Polidori G. (1994). SIR92—A program for automatic solution of crystal structures by direct methods. J. Appl. Crystallogr..

[85] Sheldrick G.M. (2008). A short history of SHELX. Acta Crystallogr. Sect. A.

