# Supplementary material for: Can Serendipity Still Hold Any Surprises in the Coordination Chemistry of Mixed-Donor Macrocyclic ligands? The Case Study of Pyridine-Containing 12-Membered Macrocycles and Platinum Group Metal ions PdII, PtII, and RhIII
Source: Molecules. 2021 Feb 27;26(5):1286. doi: 10.3390/molecules26051286 (PMC7956204; doi:10.3390/molecules26051286)
Supplement: Supplementary file 1 [file molecules-26-01286-s001.pdf]

Can Serendipity Still Hold Any Surprises in the Coordination Chemistry of Mixed Donor Macrocyclic ligands? The Case Study of Pyridine-Containing 12-Membered Macrocycles and Platinum Groupd Metal Ions Pd^{II}, Pt^{II}, and Rh^{III}

Alessandra Garau, Giacomo Picci, Massimiliano Arca, Alexander J. Blake, Claudia Caltagirone, Greta De Filippo, Francesco Demartin, Francesco Isaia, Vito Lippolis,* Anna Pintus, Andrea M. Scorciapino M. Carla Aragoni

Supplementary Information

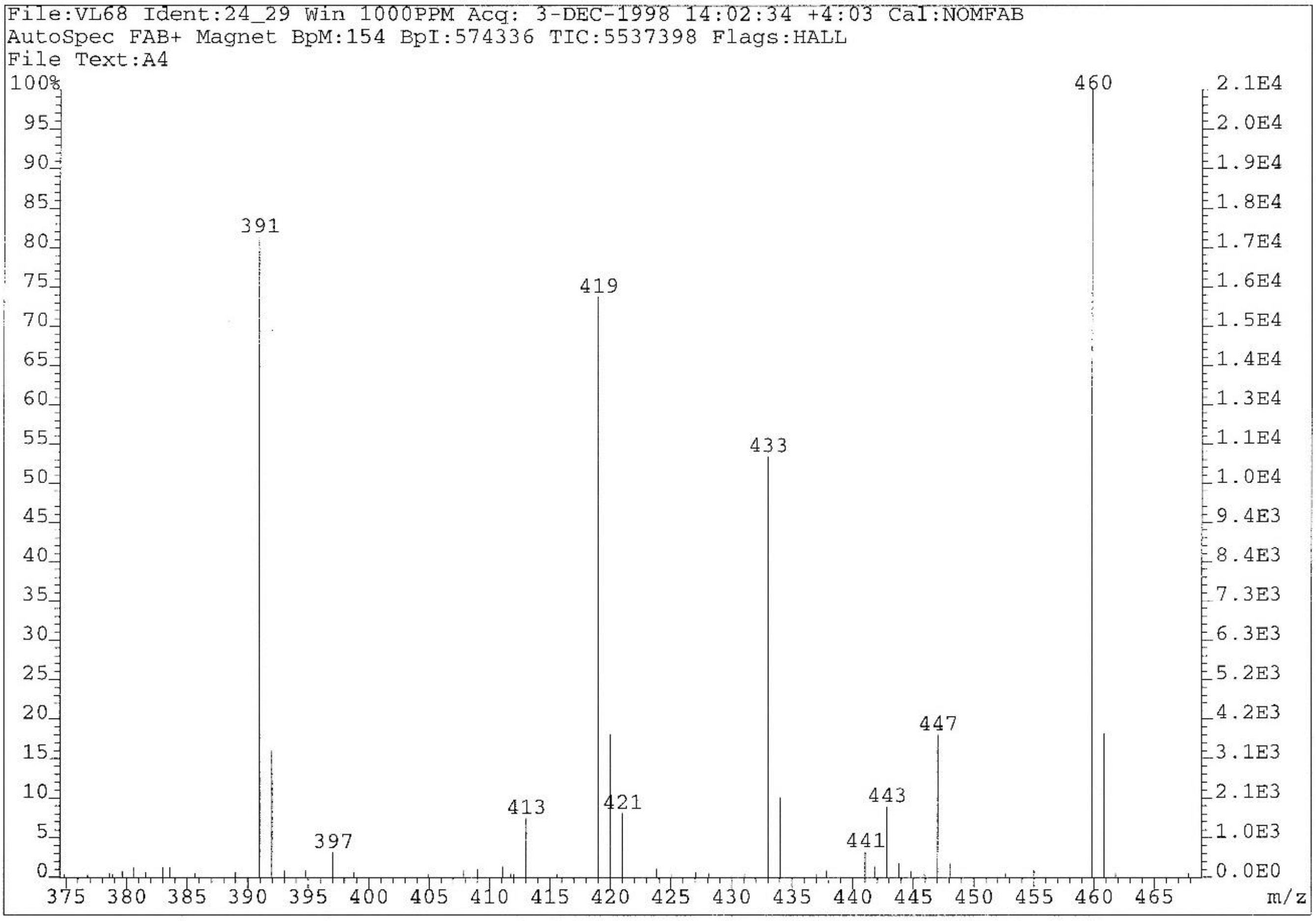

Figure S1. FAB Mass Spectrum of $[Pd(\mathbf{L}^1)Cl]_2[Pd_2Cl_6]$.

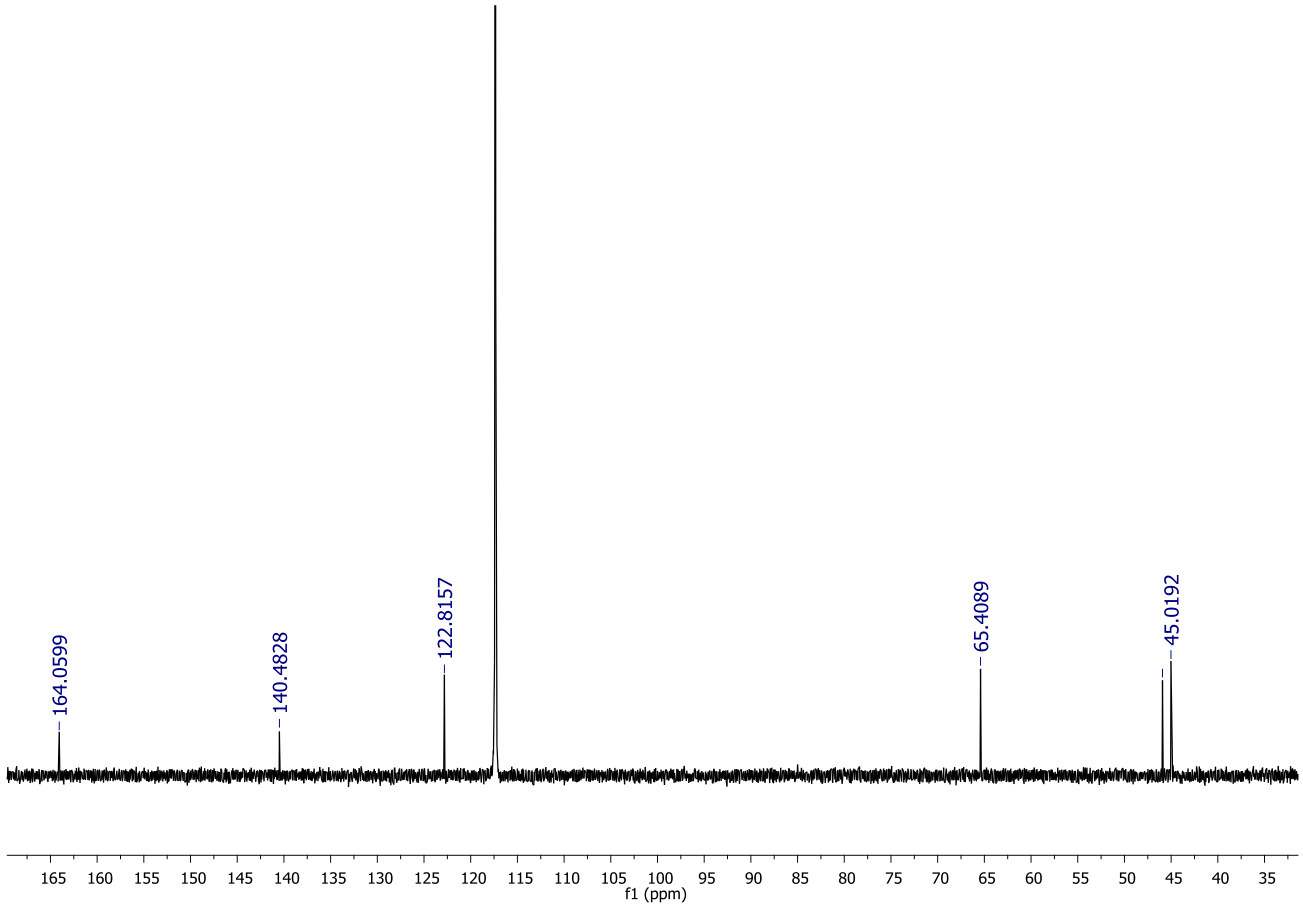

Figure S2. ^{13}C-NMR of $[Pd(\mathbf{L}^1)Cl]_2[Pd_2Cl_6]$ recorded in CD_3CN (150.9 MHz).

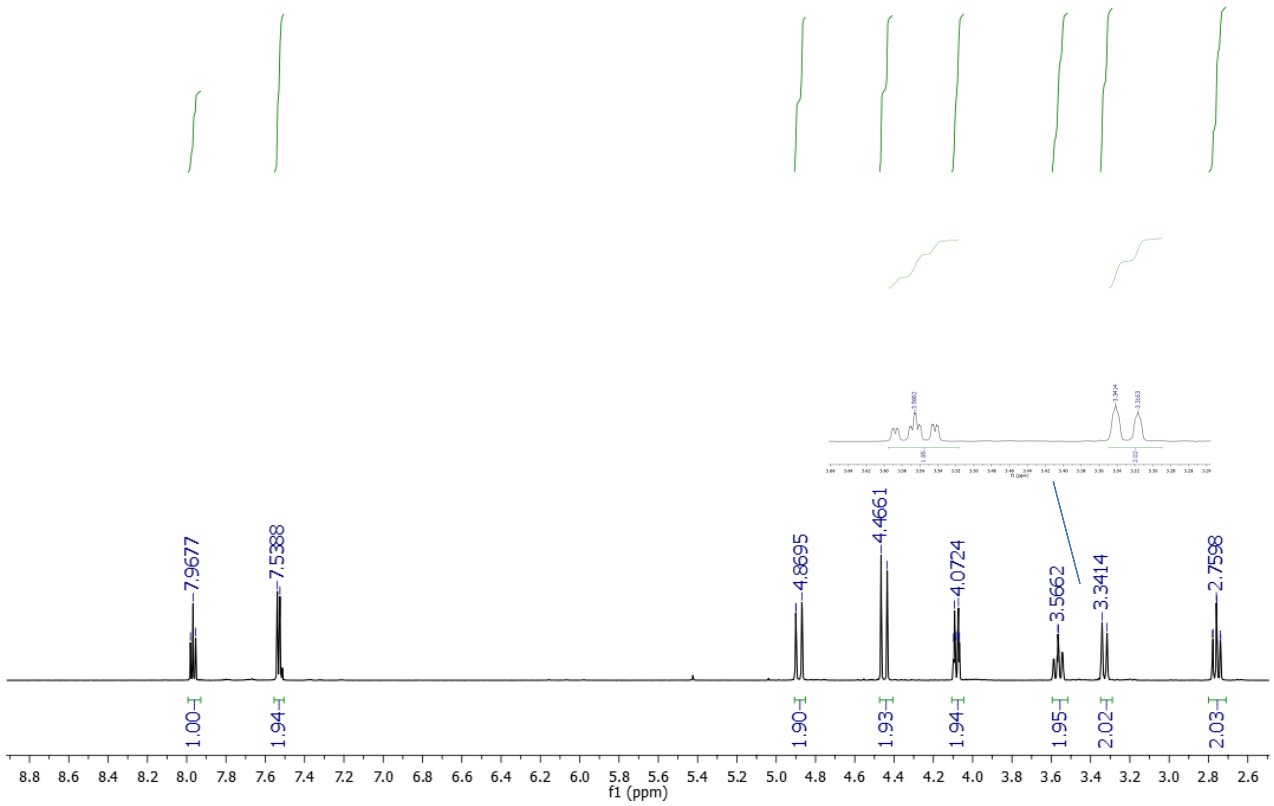

Figure S3. ^{1}H-NMR of [Pd(**L**^{1})Cl]$_2$[Pd$_2$Cl$_6$] recorded in CD$_3$CN (600 MHz). In the inset an enlargement of the region 3.20-3.60 ppm.

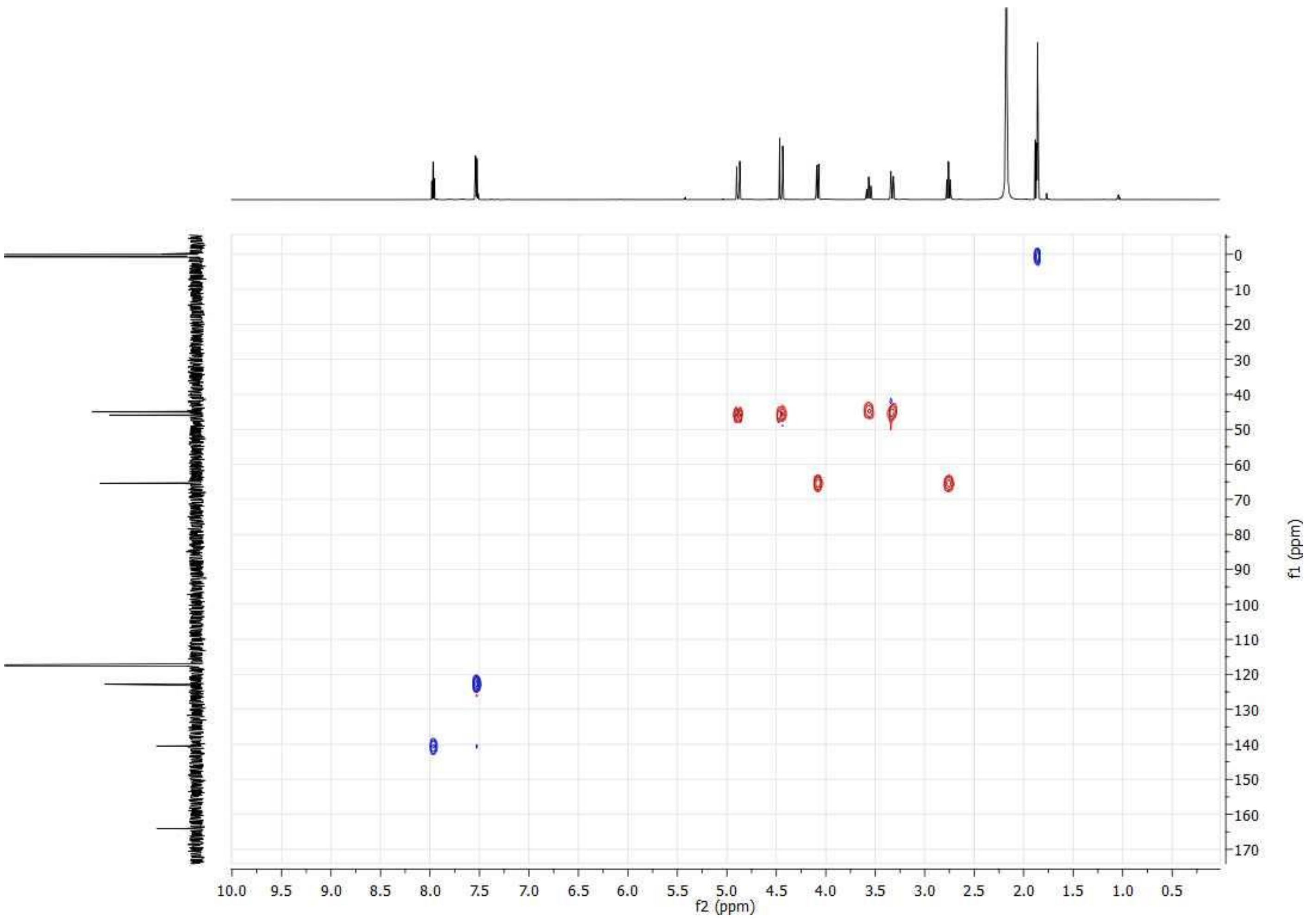

Figure S4. ^{1}H-^{13}C HSQC spectrum of [Pd(**L**^{1})Cl]$_2$[Pd$_2$Cl$_6$] recorded in CD$_3$CN on a Bruker Avance III HD 600 MHz spectrometer.

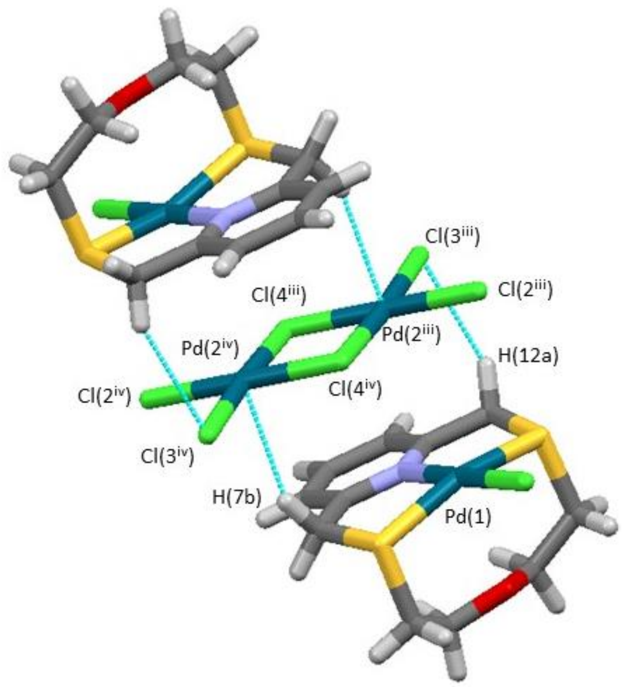

Figure S5. Details of the crystal packing of [Pd(**L**[1])Cl]$_2$[Pd$_2$Cl$_6$]. Cl3^{iii}···H12a 2.71, Cl3^{iii}···C12 3.606(6) Å, Cl3^{iii}-H12a···C12 154°; Pd2^{iv}···H7b 2.67, Pd2^{iv}···C7 3.535(5) Å, Pd2^{iv}-H7b···C7 149 °. Symmetry codes: iii = -x, 1-y, 1-z ; iv = x, -1+y, z.

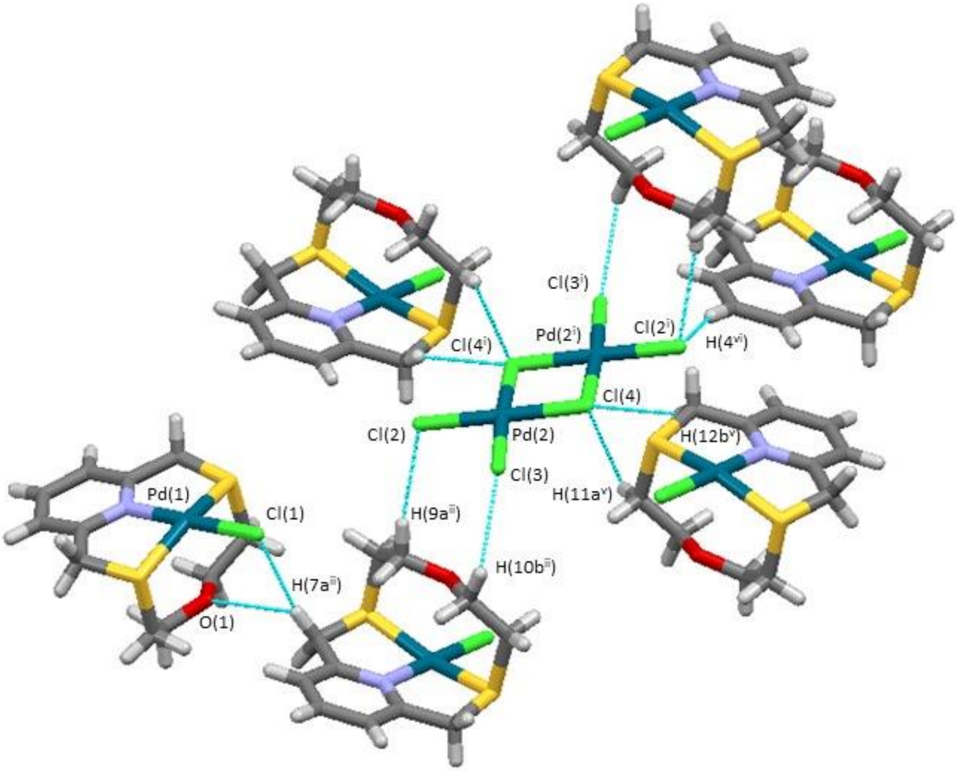

Figure S6. Details of the crystal packing of [Pd(**L**[1])Cl]$_2$[Pd$_2$Cl$_6$]. Cl1···H7a^{ii} 2.73, Cl1···C7^{ii} 3.676(5) Å, Cl1···H7a^{ii}-C7^{ii} 154º; O1···H7a^{ii} 2.80, O1···C7^{ii} 3.349(5) Å, O1···H7a^{ii}-C7^{ii} 117 °; Cl2···H9a^{ii} 2.87, Cl2···C9^{ii} 3.651(5) Å, Cl2···H9a^{ii}- C9^{ii} 138°; Cl3···H10b^{ii} 2.89, Cl3···C10^{ii} 3.701(5) Å, Cl3···H10b^{ii}-C10^{ii} 142°; Cl4···H12b^{v} 2.76, Cl4···C12^{v} 3.500(4) Å, Cl4···H12b^{v}-C12^{v} 133°; Cl4···H11a^{v} 2.83, Cl4···C11^{v} 3.640(4) Å, Cl4···H11a^{v}-C11^{v} 142°; Cl2^{i}···H4^{vi} 2.80, Cl2^{i}···C4^{vi} 3.685(5) Å, Cl2^{i}···H4^{vi}-C4^{vi} 159°. Symmetry codes: i = x, 1+y, z ; ii = ½ -x, ½ + y, ½ -z ; v = x-½, 3/2-y, z-½; vi = -½+x, ½-y, -½+z.

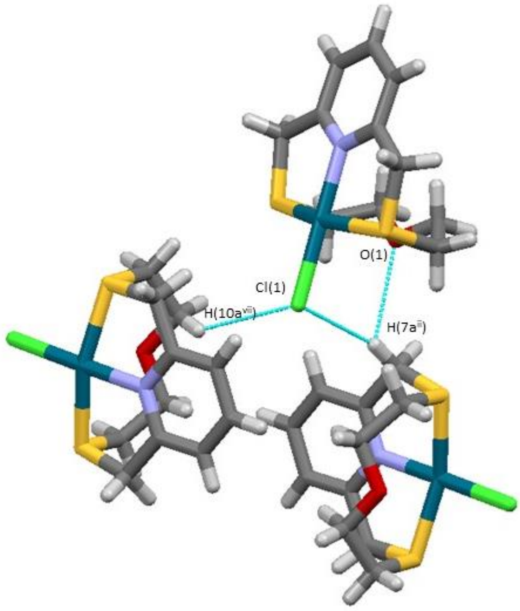

Figure S7. Details of the crystal packing of [Pd(**L**^{1})Cl]$_2$[Pd$_2$Cl$_6$]. Cl1···H10^{vii} 2.85, Cl1···C10^{vii} 3.548(4) Å, Cl1···H10^{vii}-C10^{vii} 130°. Symmetry code: vii = ½ + x, ½ - y, ½ + z.

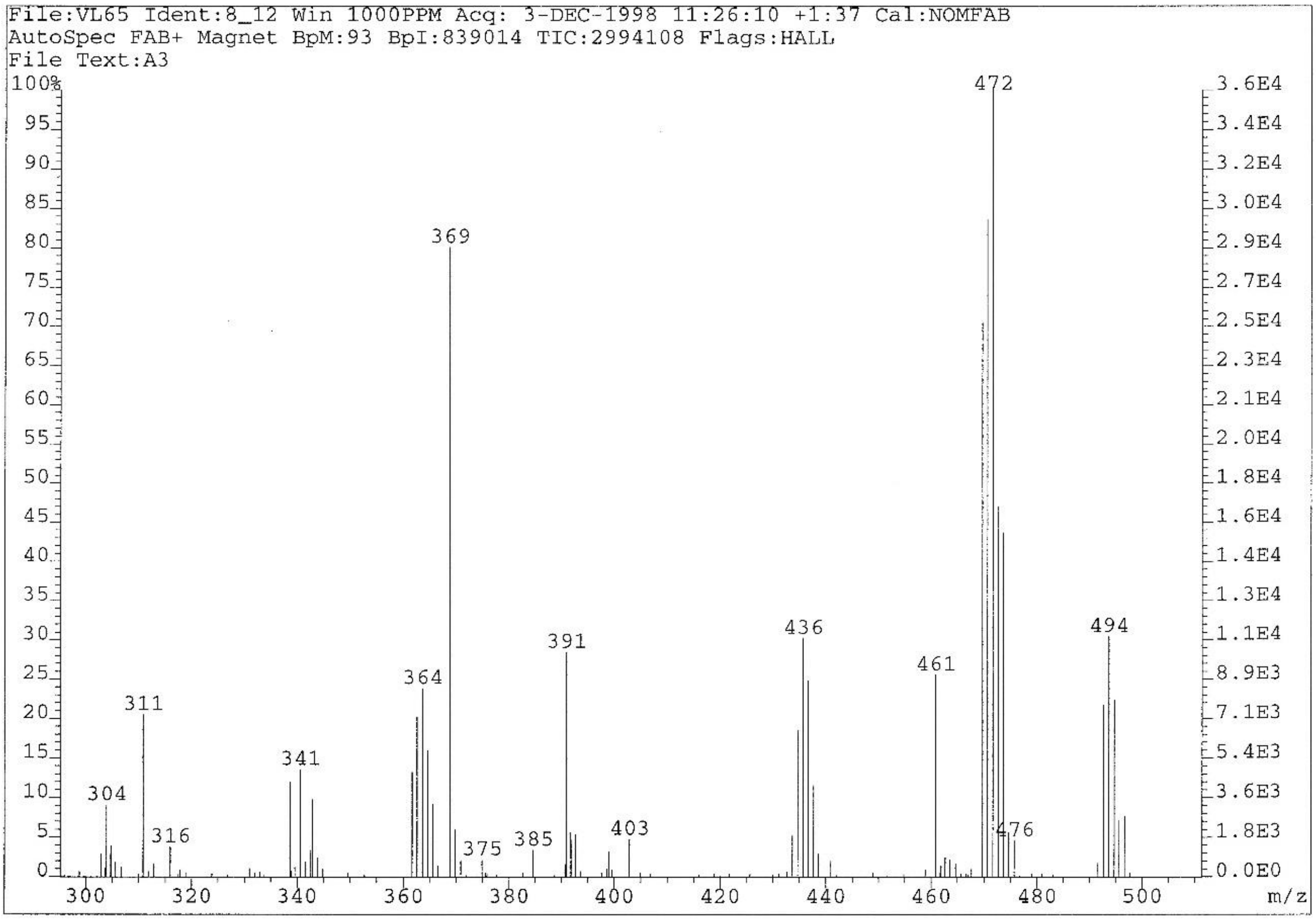

Figure S8. FAB Mass Spectrum of [Pt($\mathbf{L^1}$)Cl](BF_4).

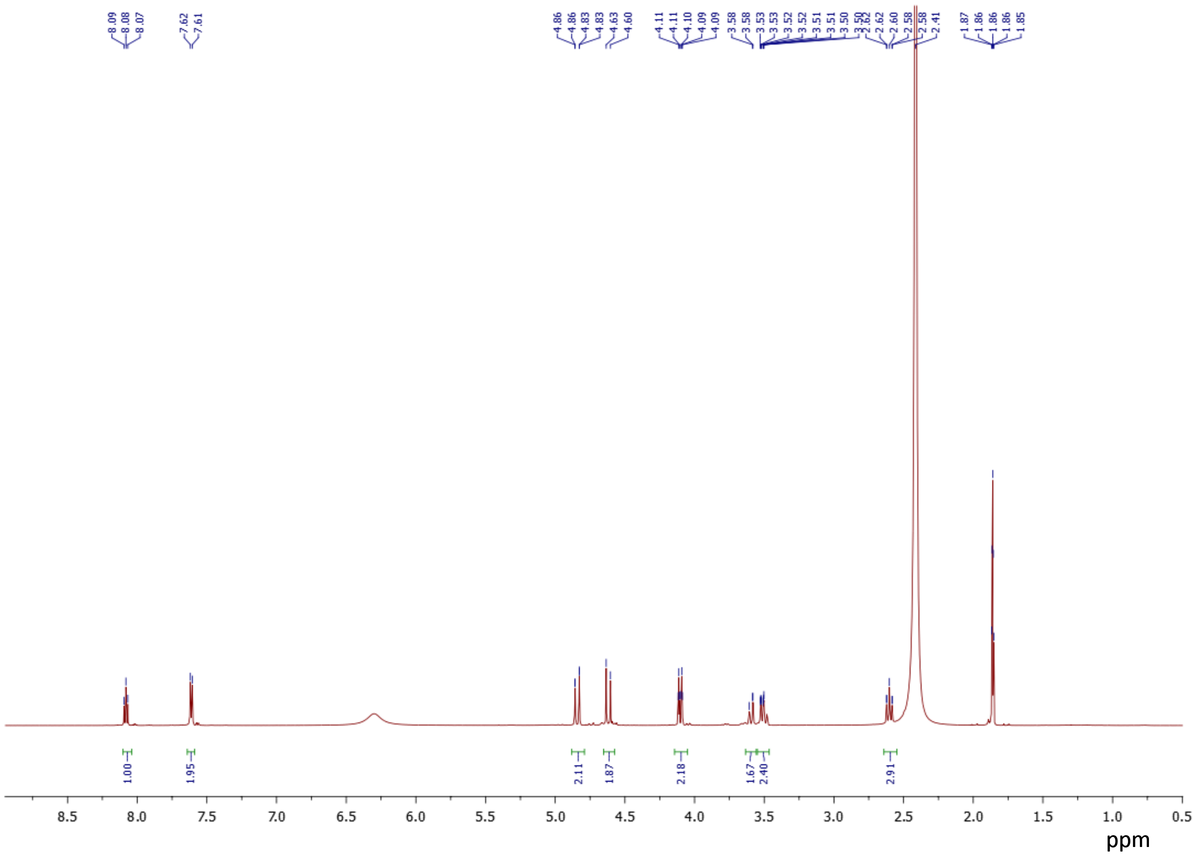

Figure S9. ^{1}H-NMR of [Pt(**L**[1])Cl](BF_4) recorded in CD_3CN (600 MHz). The broad peak at 6.3 ppm can be assigned to a tiny impurity of ammonium ions.

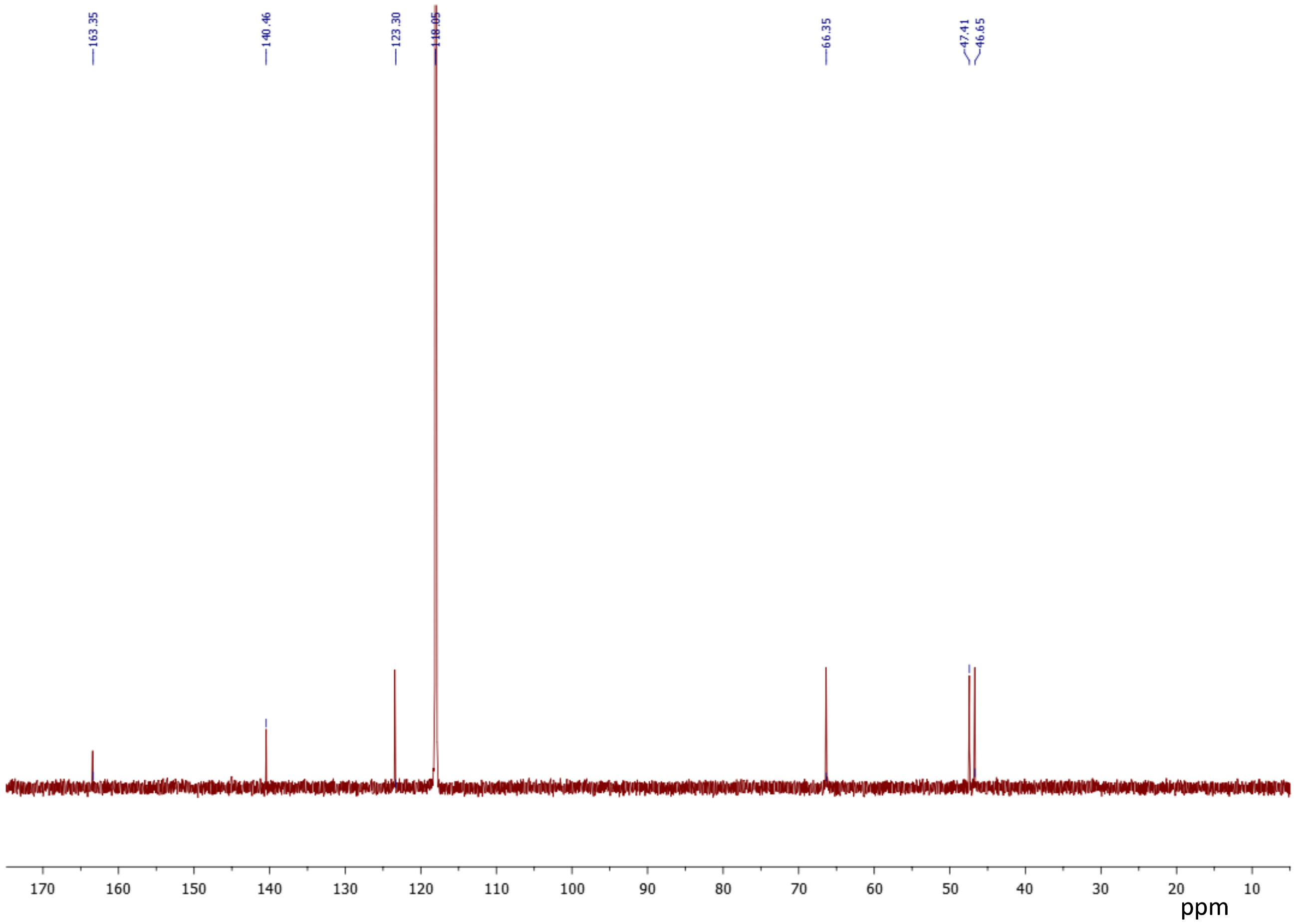

Figure S10. ^{13}C-NMR of [Pt(**L**^{1})Cl](BF_4) recorded in CD_3CN (150.9 MHz).

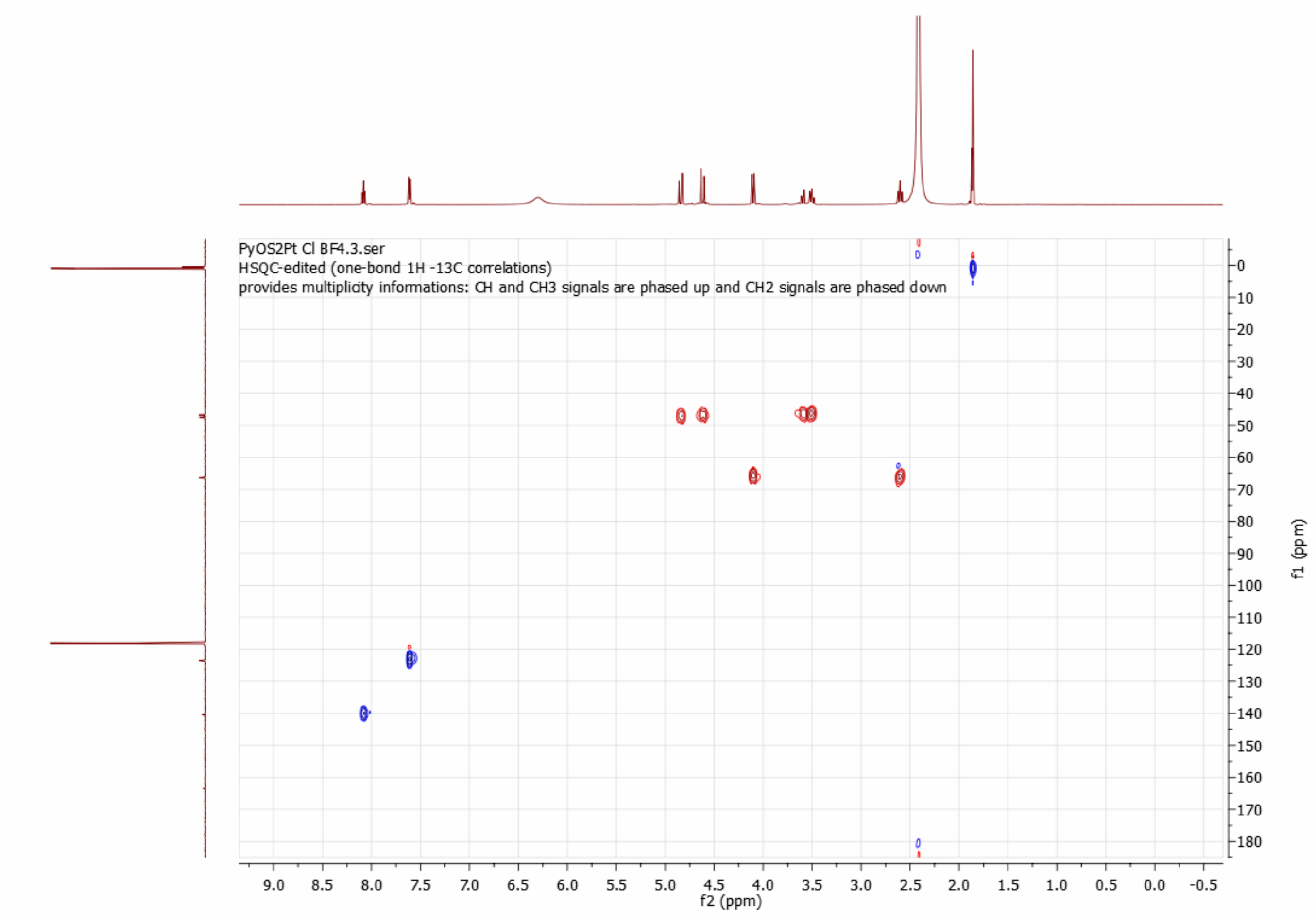

Figure S11. ^{1}H-^{13}C HSQC spectrum of [Pt(**L**[1])Cl](BF$_4$) recorded in CD$_3$CN on a Bruker Avance III HD 600 MHz spectrometer.

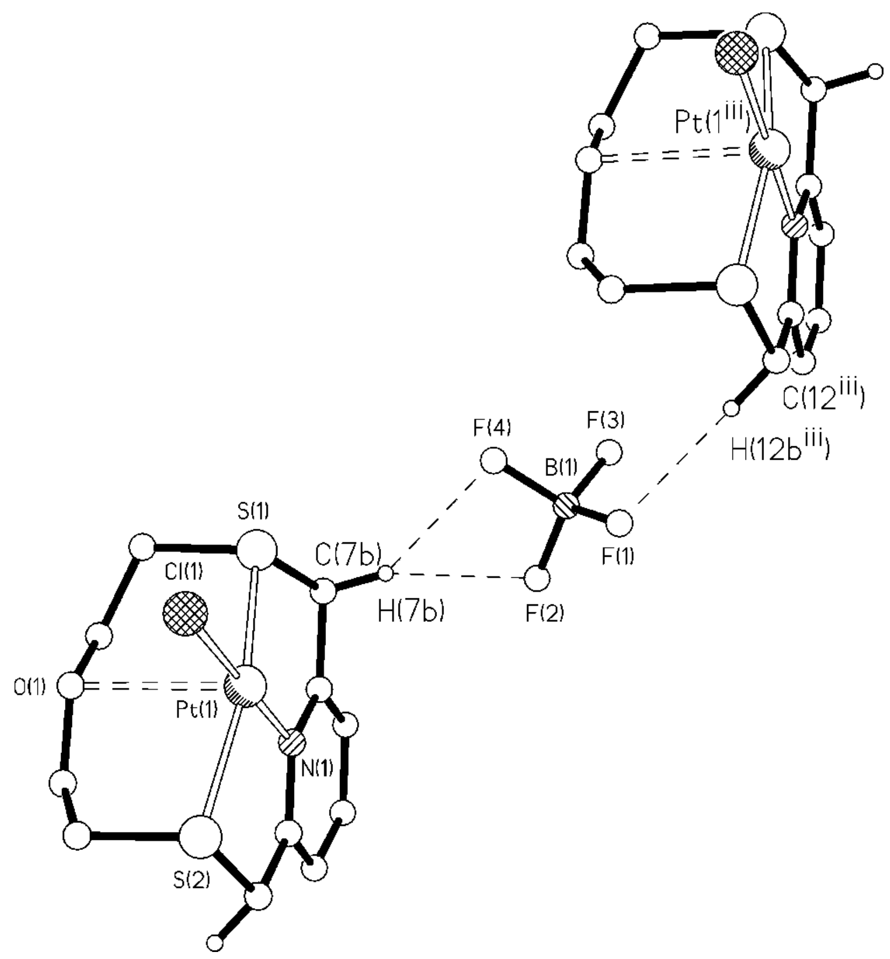

Figure S12. Partial view of the packing along the [010] direction for the compound [Pt(**L**^{1})Cl](BF_4). Only H atoms involved in the relevant H-bonds are shown for clarity. F1···H12b^{iii} 2.39, F1···C12^{iii} 3.358(6) Å, F1···H12^{iii}-C12^{iii} 165°, F2···H7b 2.48, F2···C7 3.37(1) Å, F2···H7b-C7 150°, F4···H7b 2.38, F4···C7 3.274(8) Å, F4···H7b-C7 160°. Symmetry code: iii = 1 + x, y, -1 + z.

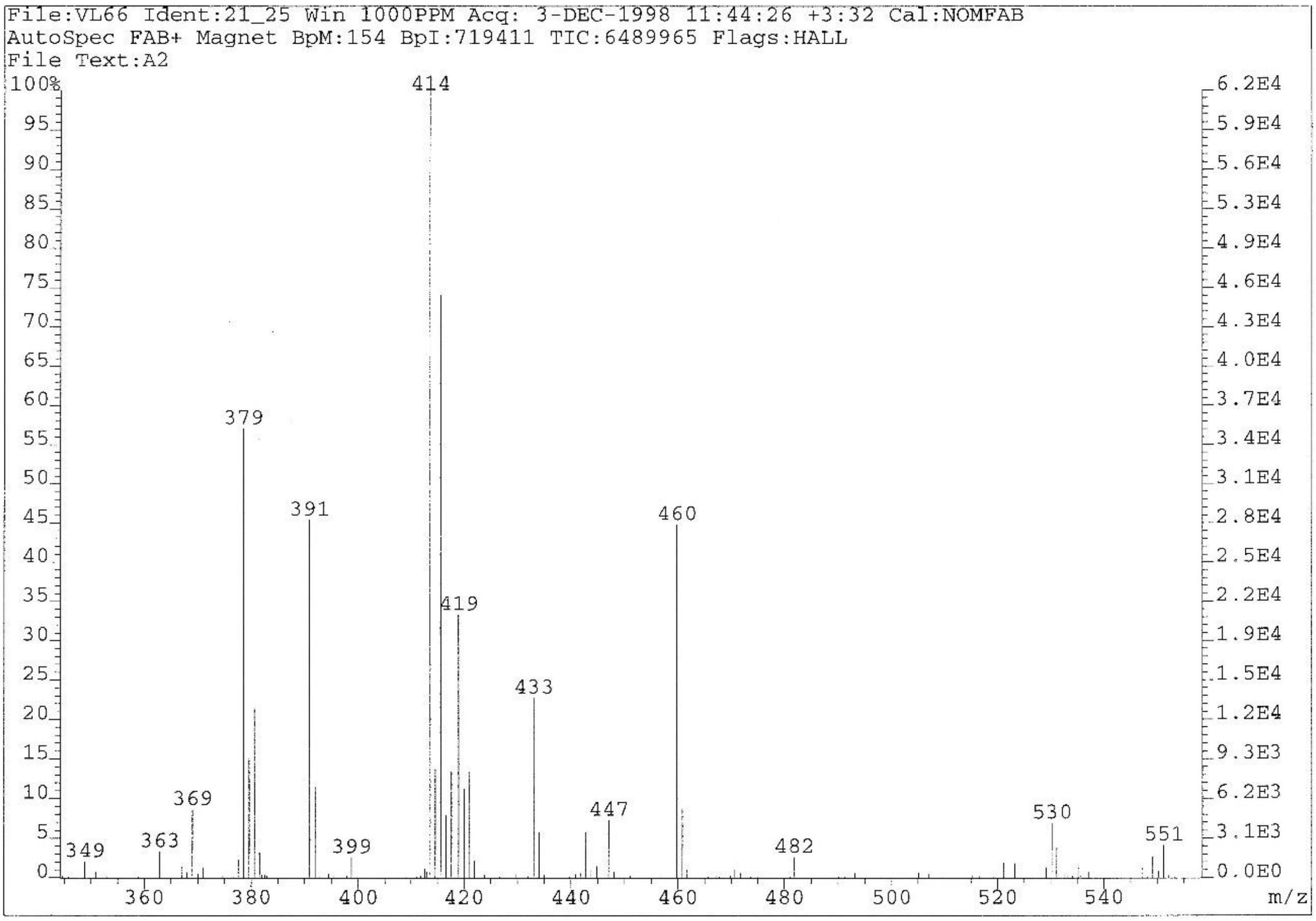

Figure S13. FAB Mass Spectrum of [Rh($\mathbf{L}^1$)Cl_2](PF_6).

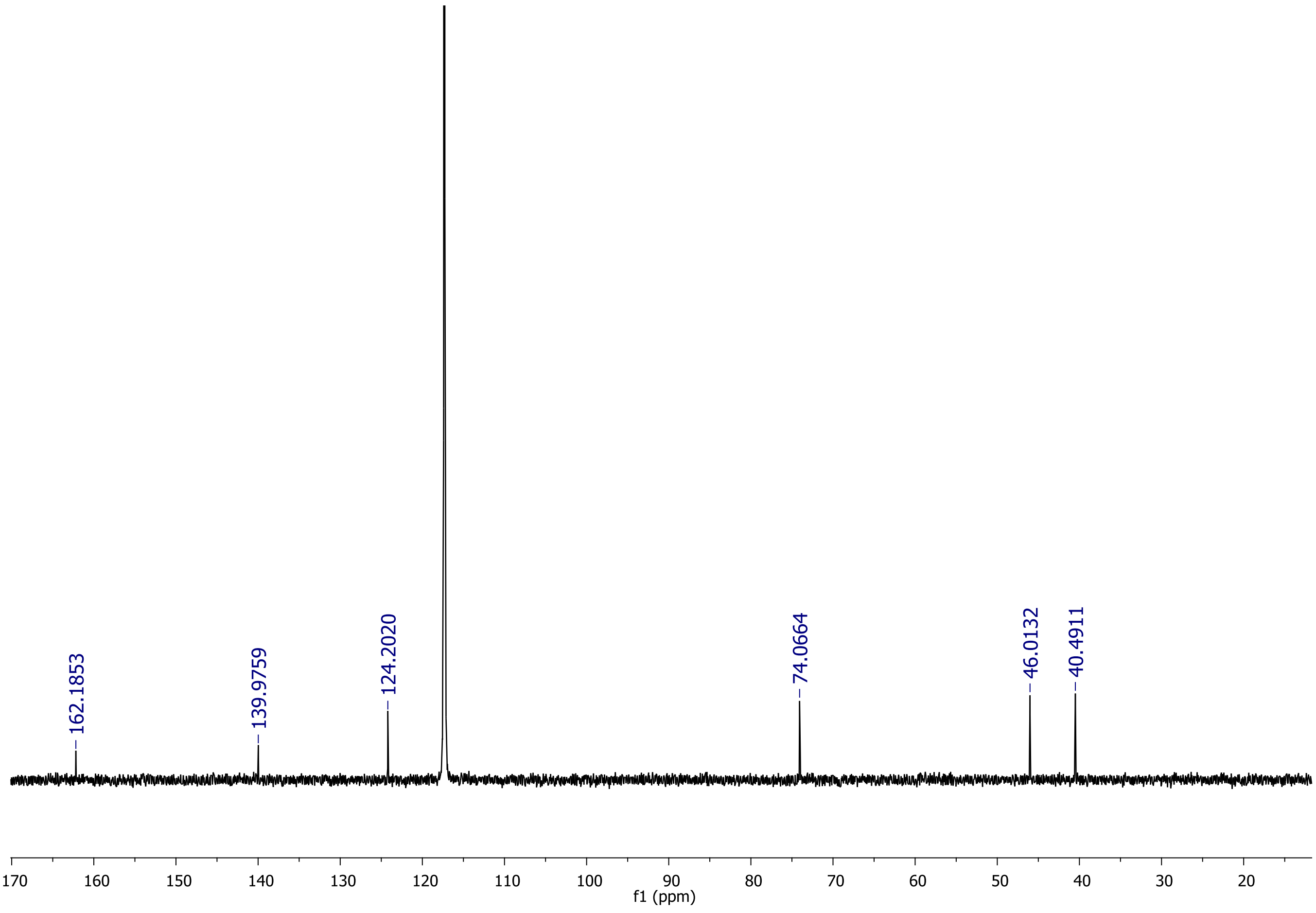

Figure S14. ^{13}C-NMR of $[Rh(\mathbf{L}^1)Cl_2](PF_6)$ recorded in CD_3CN (150.9 MHz).

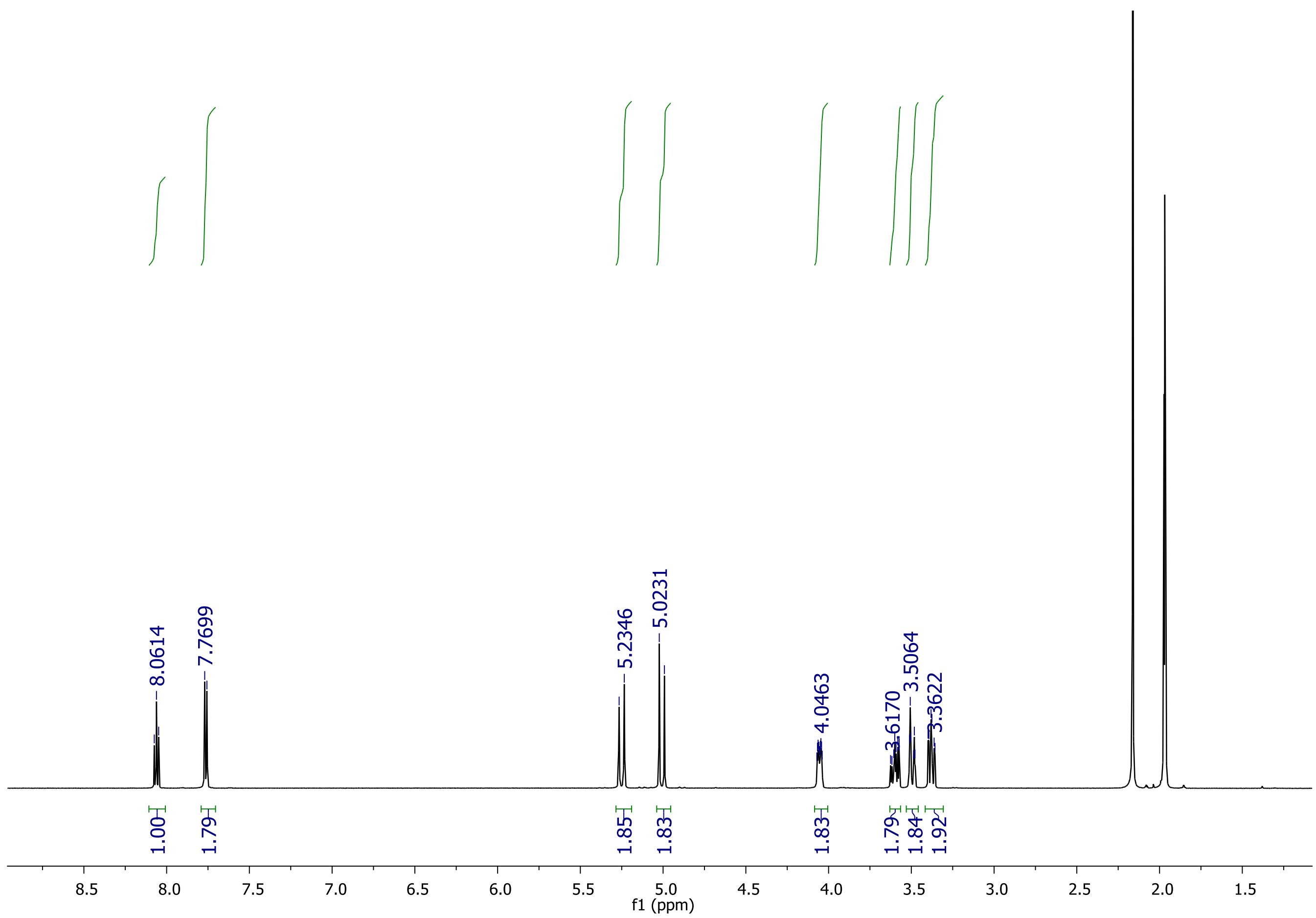

Figure S15. ^{1}H-NMR of [Rh(**L**^{1})Cl_2](PF_6) recorded in CD_3CN (600 MHz).

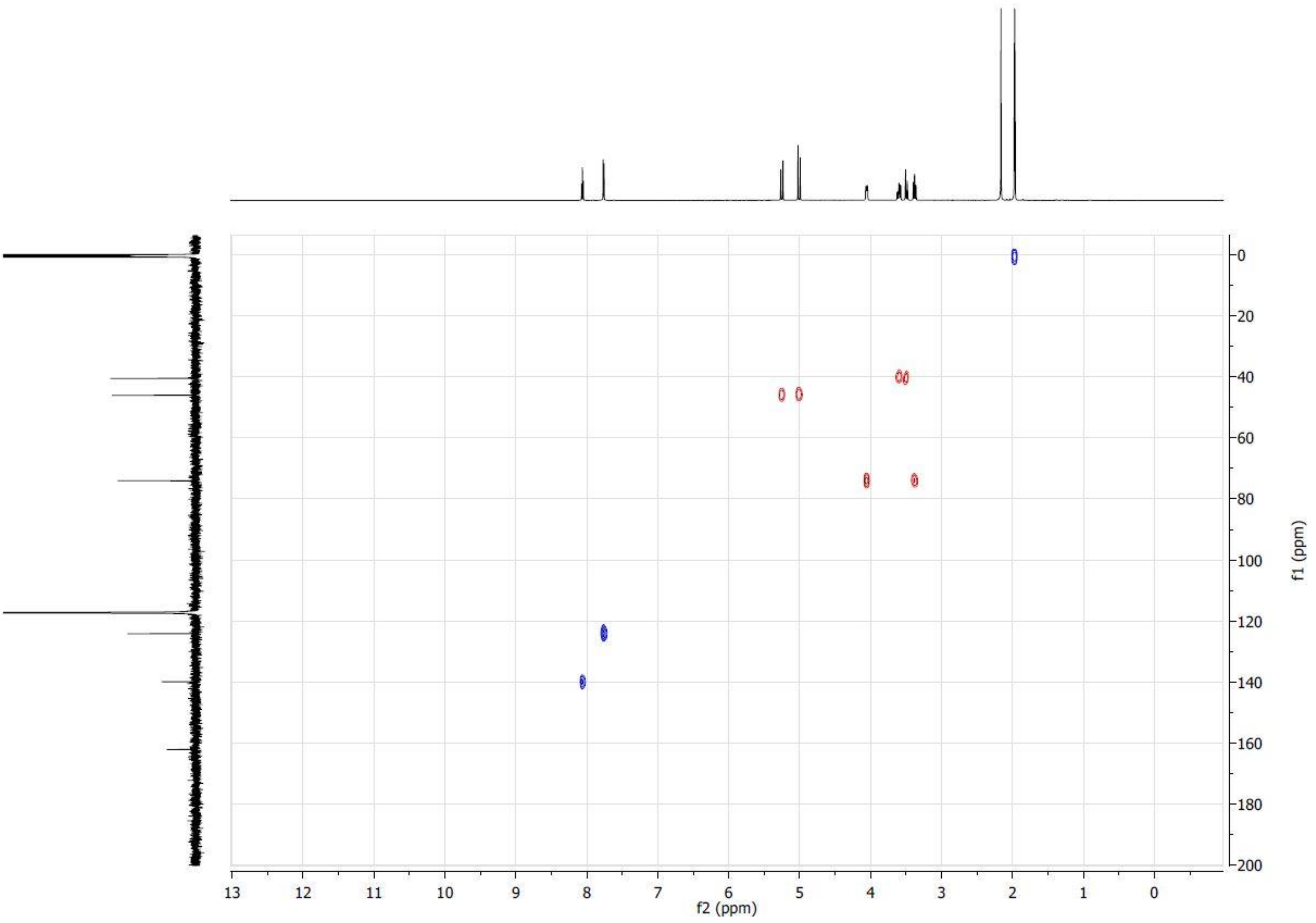

Figure S16. ^{1}H-^{13}C HSQC spectrum of $[Rh(\mathbf{L^1})Cl_2](PF_6)$ recorded in CD_3CN on a Bruker Avance III HD 600 MHz spectrometer.

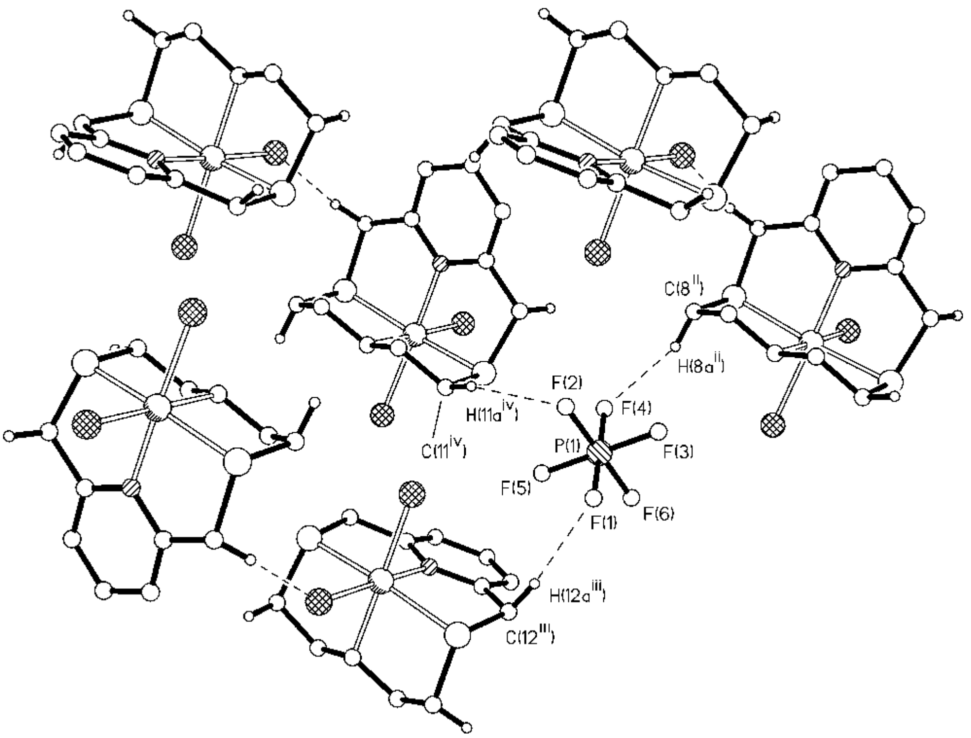

Figure S17. Partial view of the packing along the [0-11] direction for the compound [Rh(**L**^{1})Cl_2](PF_6). Only H atoms involved in the relevant H-bonds are shown for clarity. F1···H12a^{iii} 2.49 Å, F1···C12^{iii} 3.450(8) Å, F1···H12a^{iii}-C12^{iii} 169°, F2···H11a^{iv} 2.53 Å, F2···C11^{iv} 3.427(9) Å, F2···H11a^{iv}-C11^{iv} 154°, F4···H8a^{ii} 2.33 Å, F4···C8^{ii} 3.209(9) Å, F4···H8a^{ii}-C8^{ii} 150°. Symmetry codes: ii = -x, 1-y, 1-z; iii = 1-x, ½ + y, 3/2 –z; iv = 1-x, 1-y, 1-z.

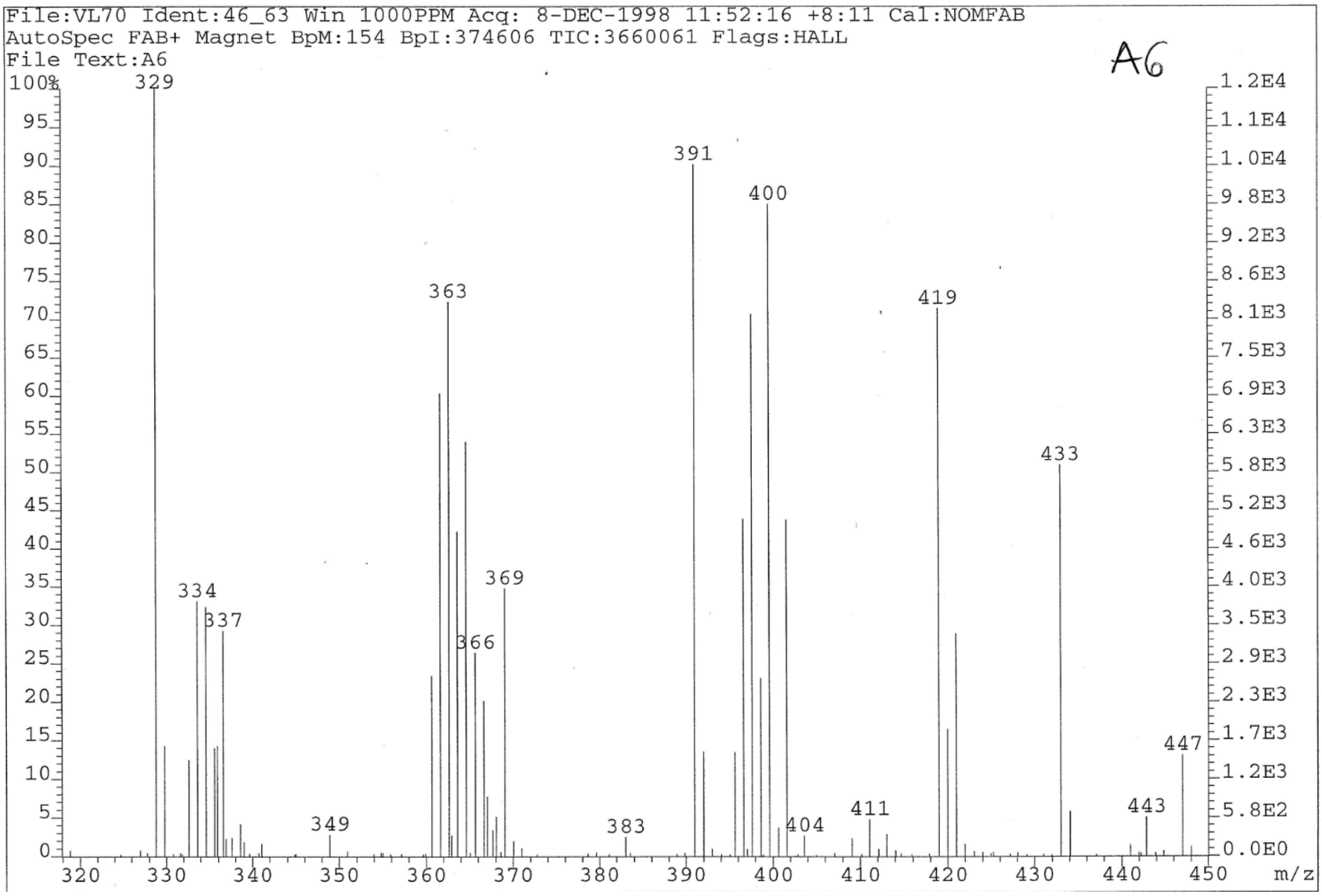

Figure S18. FAB Mass Spectrum of [Pd(**L**^{2})Cl]Cl.

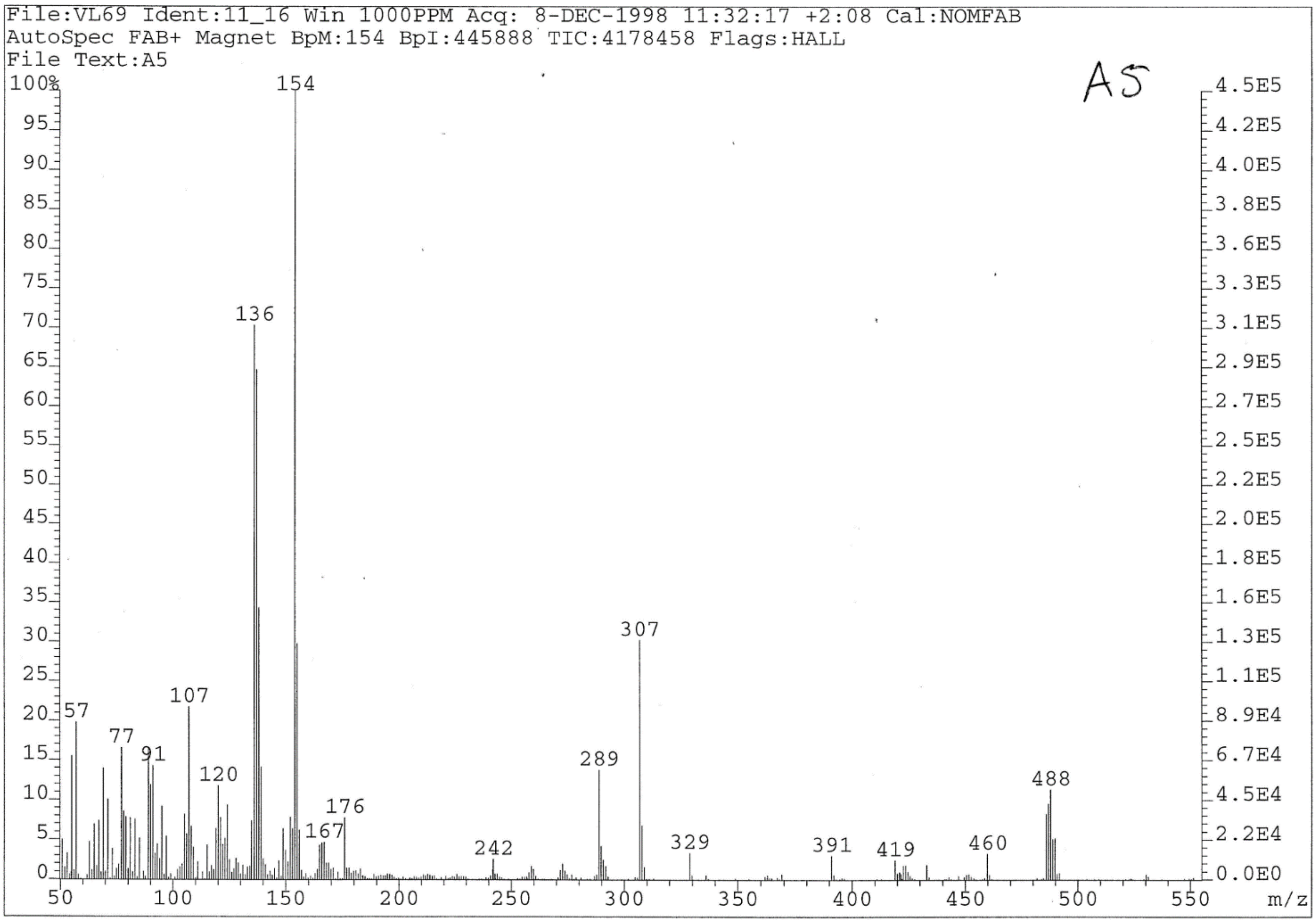

Figure S19. FAB Mass Spectrum of [Pt(**L**²)Cl]Cl.

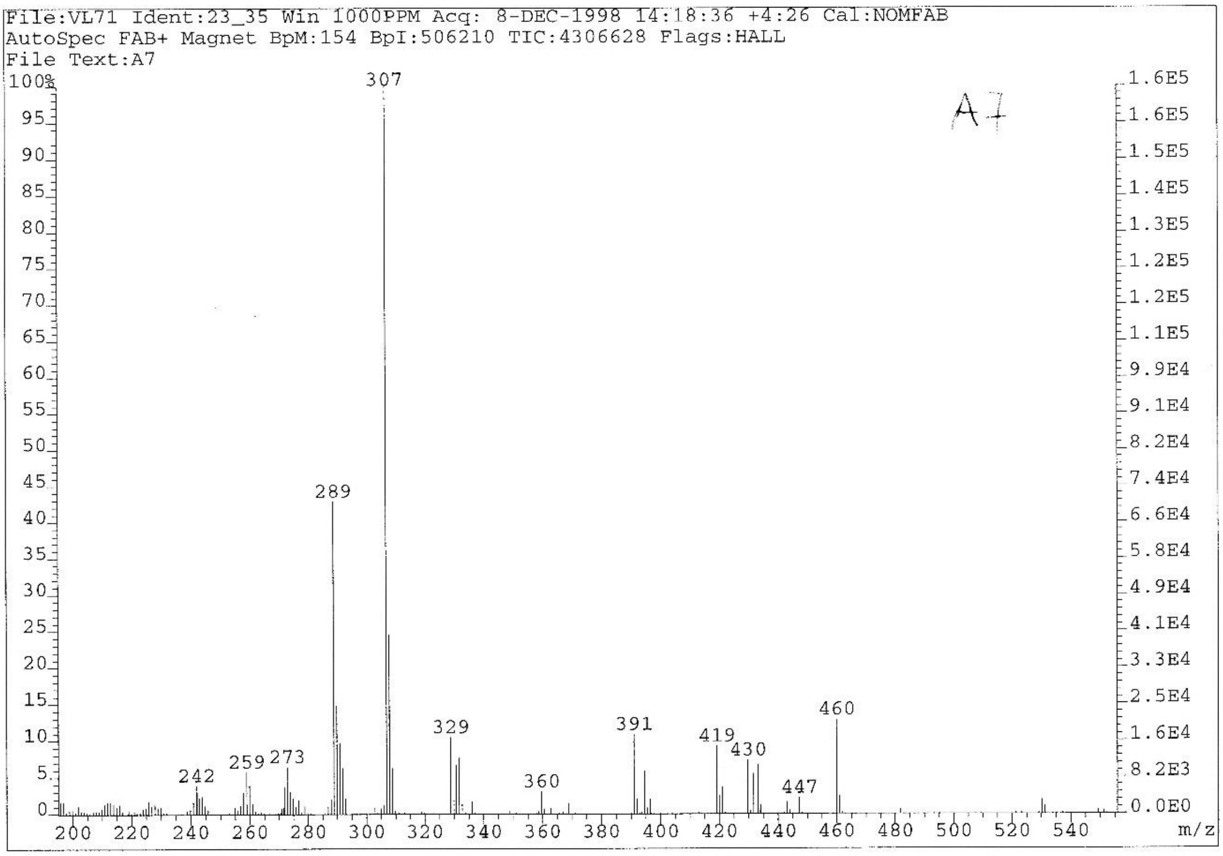

Figure S20. FAB Mass Spectrum of [Rh(L^2)Cl_2](PF_6).

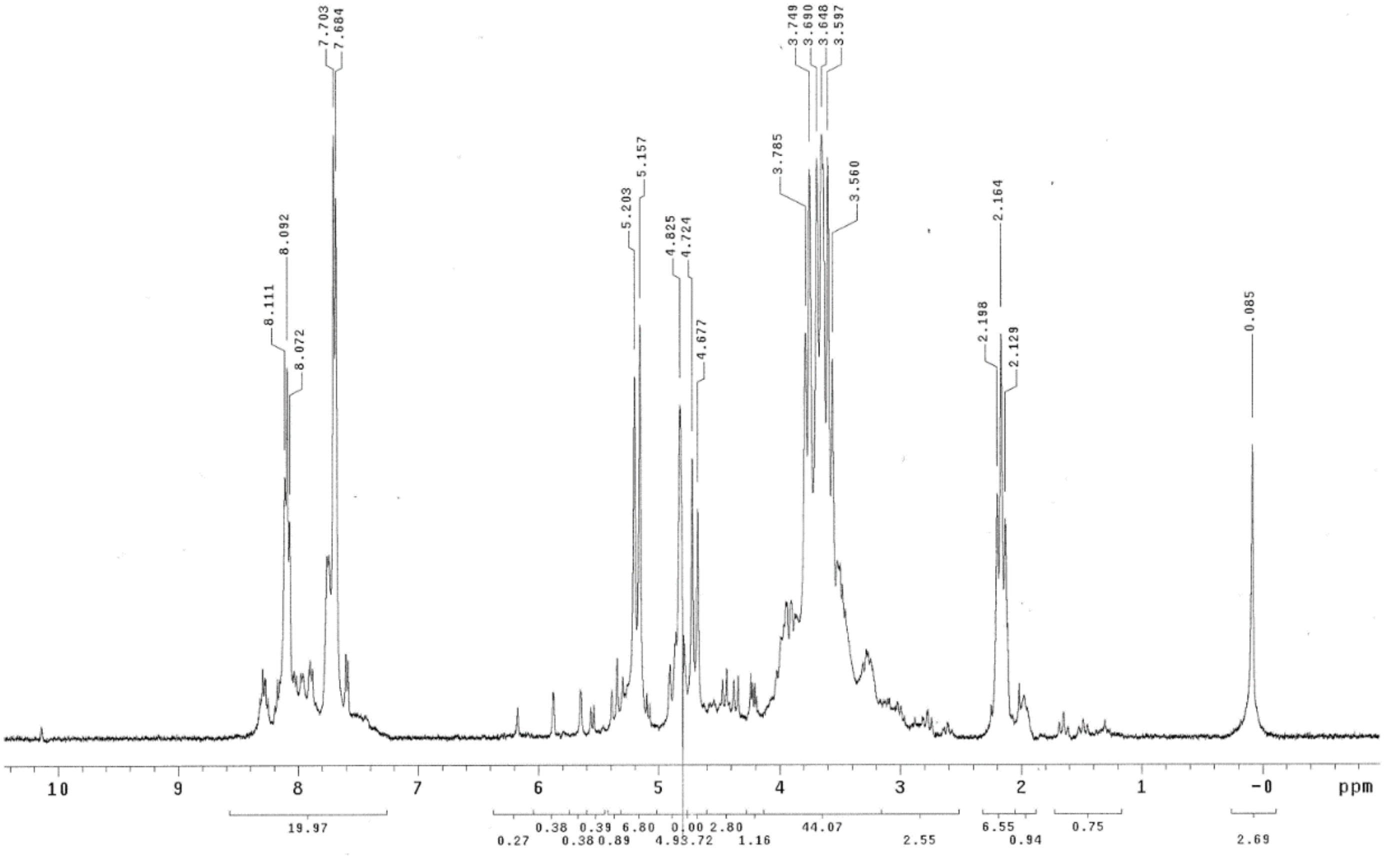

Figure S21. ^{1}H-NMR spectrum of [Pd($\mathbf{L}^2$)Cl]Cl recorded in D_2O (400 MHz).

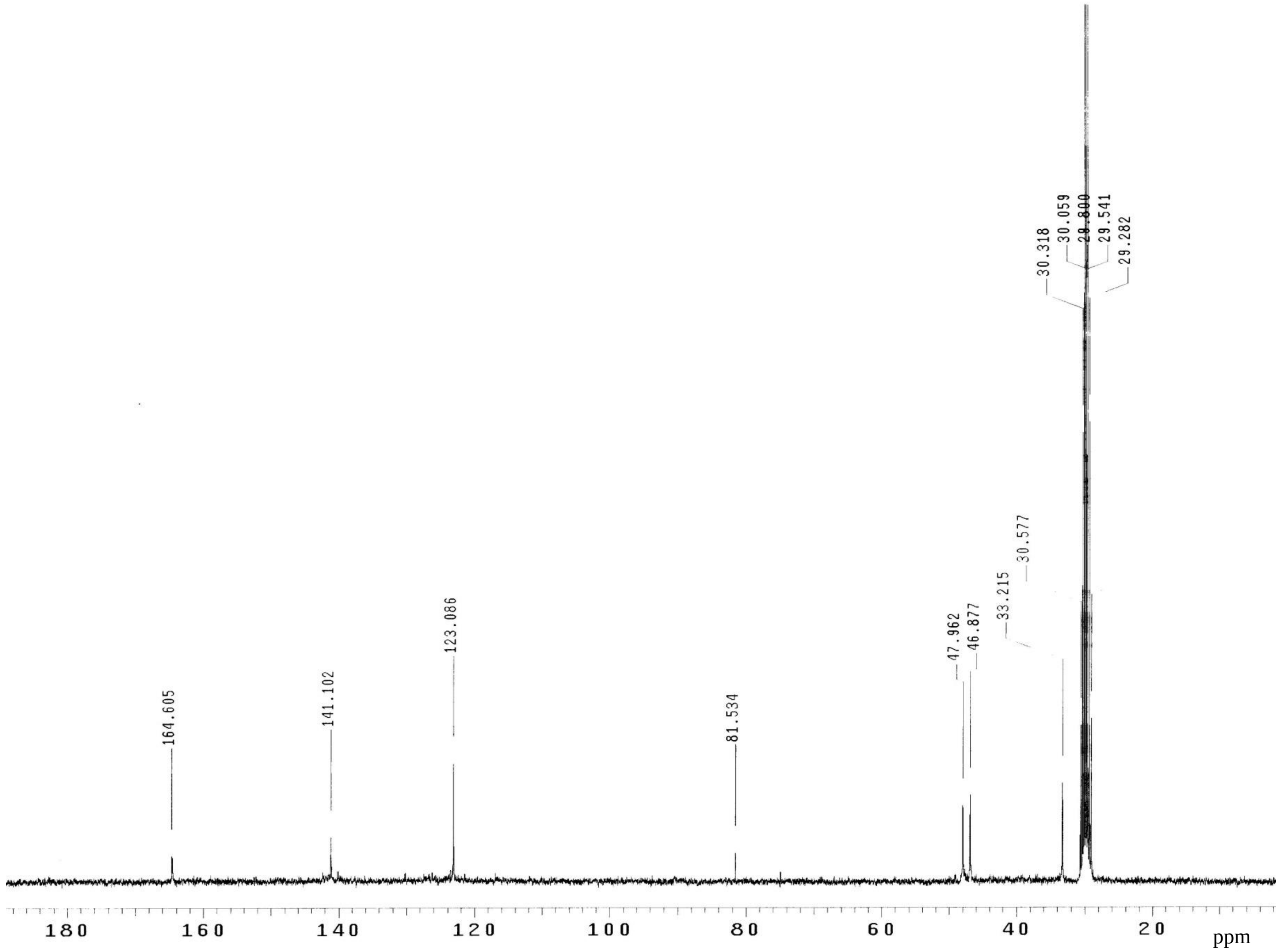

Figure S22. ^{13}C-NMR spectrum of [Pd(**L**^{2})Cl]Cl recorded in d_6-acetone (100.62 MHz).

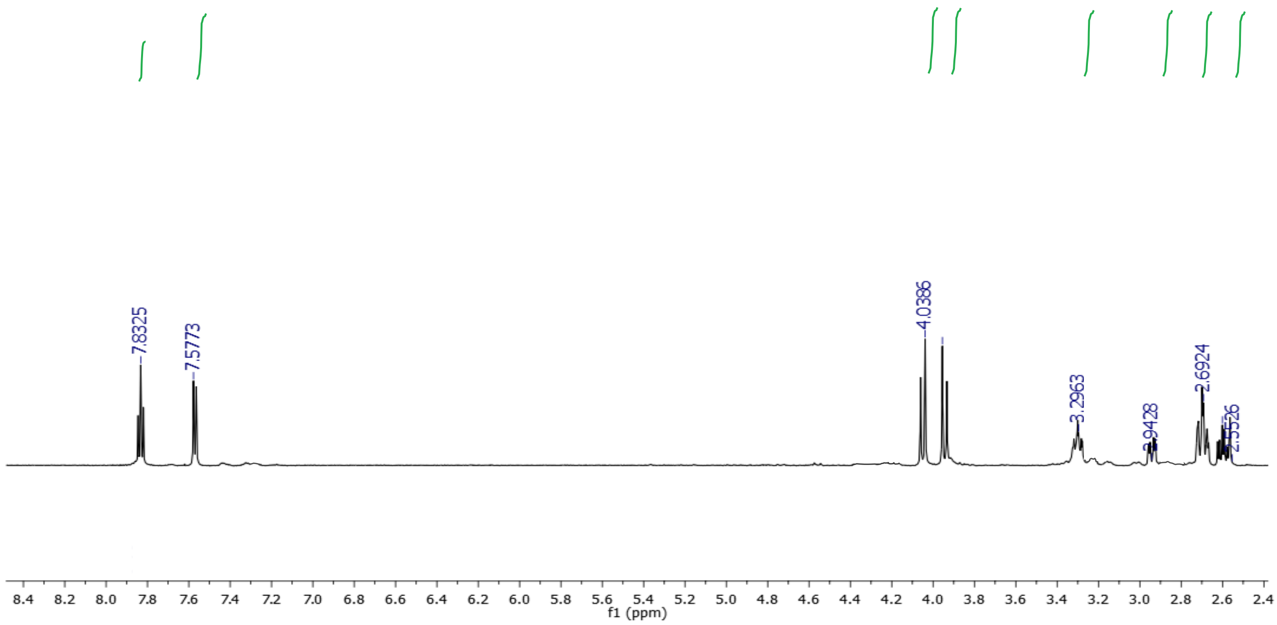

Figure S23. ^{1}H-NMR spectrum of [Pt(**L**^{2})Cl]Cl recorded in CD_3CN (600 MHz).

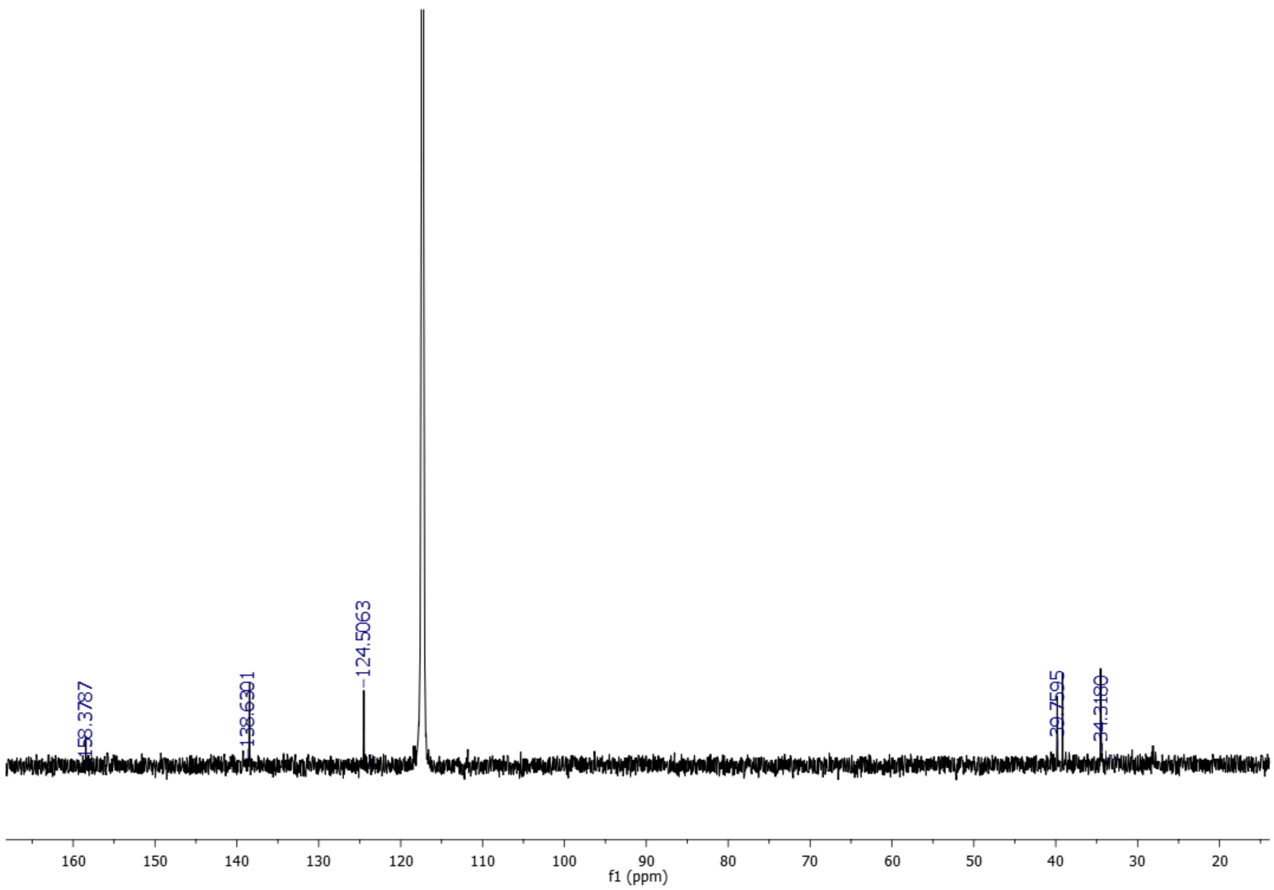

Figure S24. ^{13}C-NMR spectrum of [Pt(**L**^{2})Cl]Cl recorded in CD_3CN (150.9 MHz).

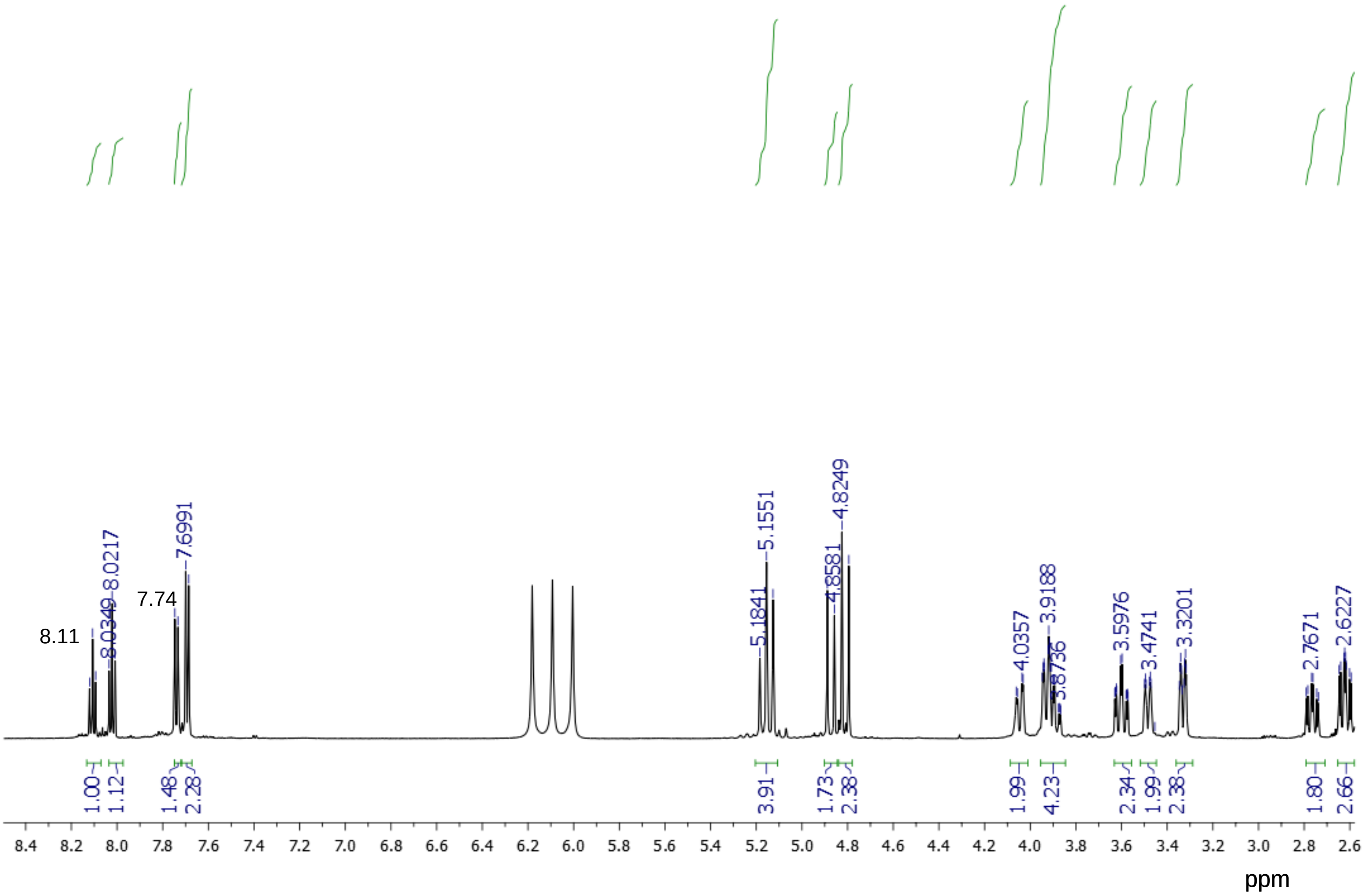

Figure S25. ^{1}H-NMR of $[Rh(\mathbf{L}^2)Cl_2](PF_6)$ recorded in CD_3CN (600 MHz).

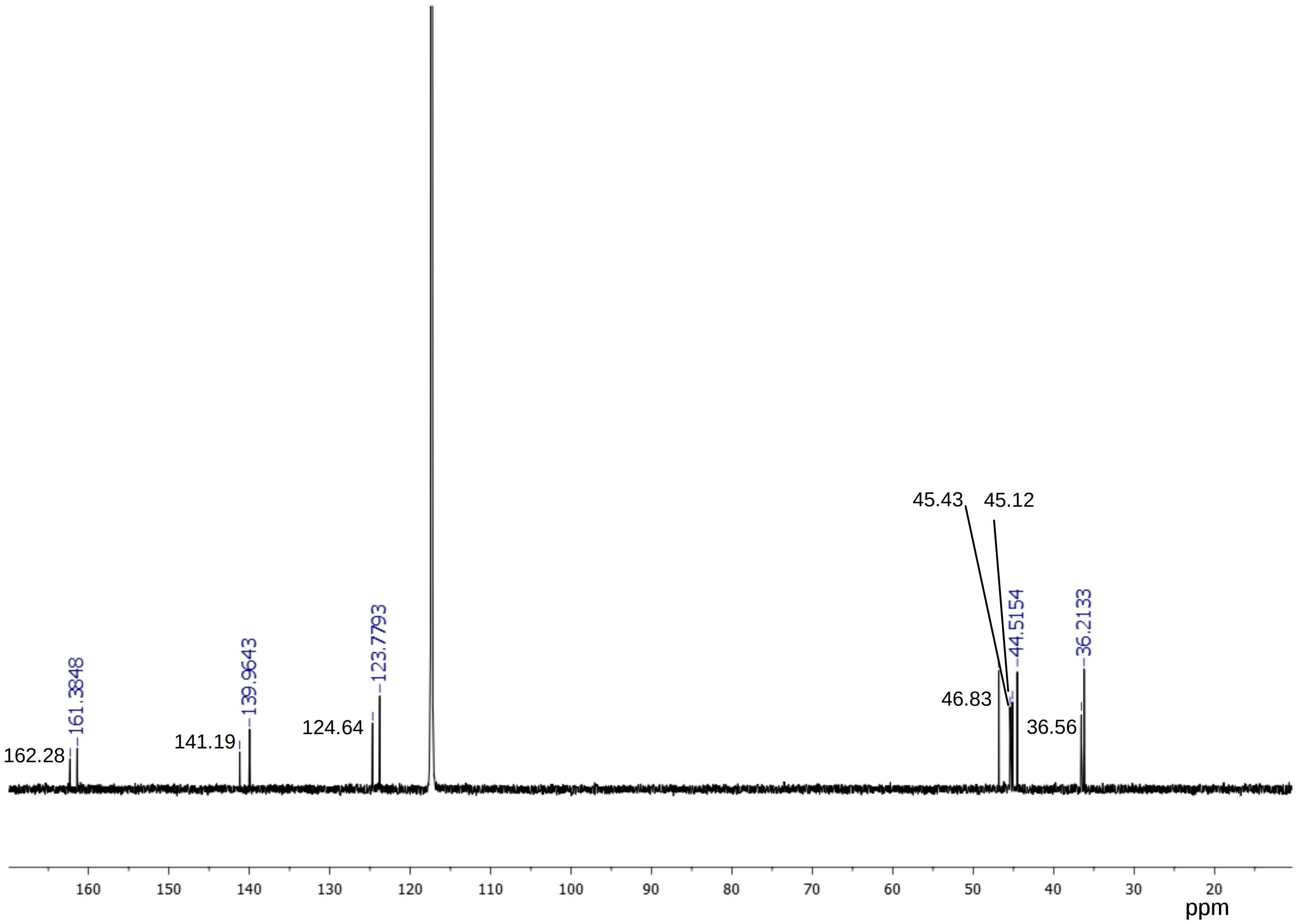

Figure S26. ^{13}C-NMR of [Rh(**L**^{2})Cl_2](PF_6) recorded in CD_3CN (150.9 MHz).

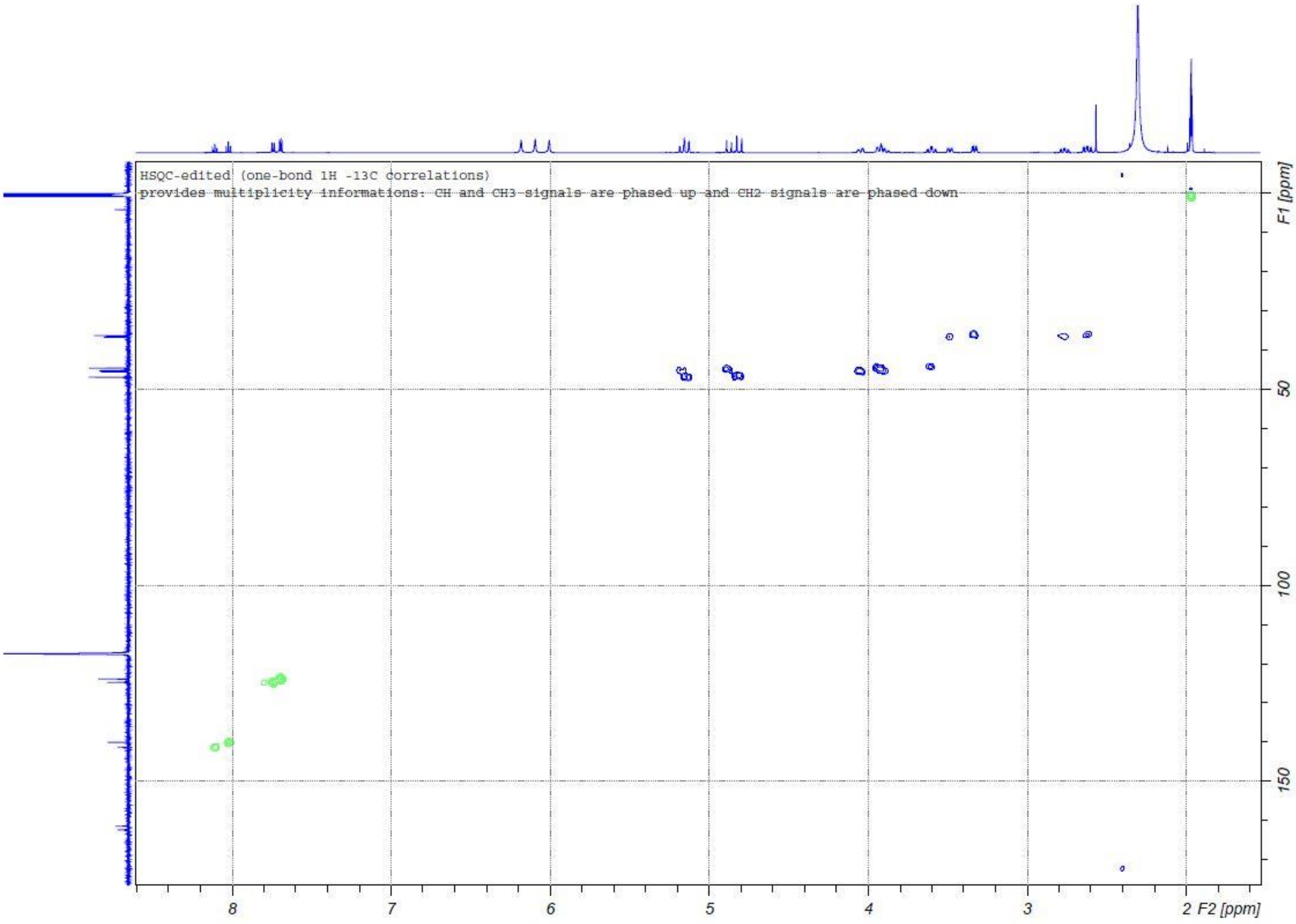

Figure S27. ^1H-^{13}C HSQC spectrum of [Rh(**L**^{2})Cl_2](PF_6) recorded in CD_3CN on a Bruker Avance III HD 600 MHz spectrometer.

Solution structures of the of L¹ and L² complexes with Rh^{III}.

First, we analyzed the NOESY spectrum (200 ms mixing time) acquired on the **L¹** complex. A clear dipolar interaction was found between both the methylene protons on C7/C12 and the aromatic protons on C5/C3. However, the intensity of the two cross-peaks was remarkably different. The intensity ratio between the cross-peak at [7.74-4.98] ppm and the one at [7.74-5.22] ppm was as large as 5.7. This means that the two geminal protons and the aromatic proton are significantly different. Cross-peak intensity is directly proportional to the inverse sixth-power of the interproton distance, so that from intensity ratio we can estimate a distance ratio of 1.34.

Among the two geminal protons, only the one resonating at 4.98 ppm showed a significant correlation with protons belonging to other methylene groups in the macrocycle, namely at 3.47 and 3.35 ppm. The latter are bound to two different carbon atoms, so that we can conclude that the six protons resonating at 4.98, 3.47 and 3.35 ppm are located on the same side of the macrocycle plane and are located at closer distance with each other as compared to the other six methylene protons. The intensity ratio between the cross-peaks at [4.98-3.47] ppm and [4.98-3.35] ppm is 2.4, corresponding to a distance ratio of 1.16. All of these observations are incompatible with a *trans* configuration. Figure S28 shows the only molecular model compatible with our results, which is in agreement with the crystal structure shown in Figure 5.

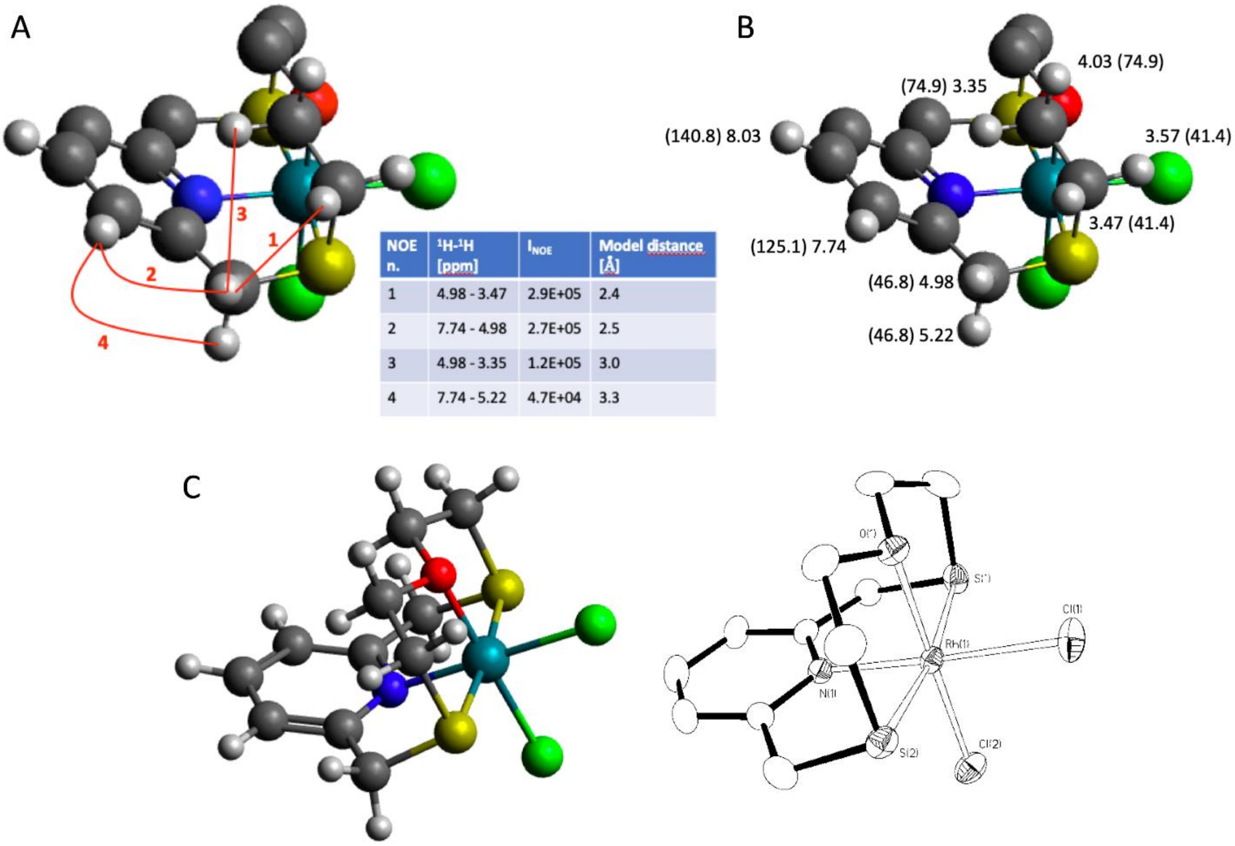

NOE n.	¹H-¹H [ppm]	I_{NOE}	Model distance [Å]
1	4.98 - 3.47	2.9E+05	2.4
2	7.74 - 4.98	2.7E+05	2.5
3	4.98 - 3.35	1.2E+05	3.0
4	7.74 - 5.22	4.7E+04	3.3

Figure S28. (A) Model of the dichlorido complex cation $[Rh(\mathbf{L}^2)Cl_2]^+$ is shown. Only the hydrogen atoms pointing at the observer are reported for clarity. The identified NOEs are reported as red lines and are numbered in decreasing intensity order. The table beside the figure shows the strong inverse correlation between the NOE intensity and the corresponding interproton distance in the model. (B) Complete resonance assignments are shown. ¹H chemical shift [ppm] is reported beside the protons together with the corresponding ¹³C chemical shift in parenthesis. (C) Another perspective of the solution structure is proposed, showing a perfect agreement with the crystal structure of the complex cation $[Rh(\mathbf{L}^2)Cl_2]^+$ (see figure 5).

When ¹H-NMR spectrum of the **L²** complexes is compared to that of the **L¹** complex, it is clear that all the complexes share the same fine structure for all the resonances. In addition, the NOEs pattern resulted to be very similar for the two **L²** complexes present in solution and both of them were similar to the complex formed

by $\mathbf{L}^1$. Slight differences were found in the intensity ratio and, as a consequence, in the ratio between interproton distances. In particular, distance ratio between the two protons on the C7/C12 and the one on C3/C5 was 1.34, 1.25 and 1.21 for $\mathbf{L}^1$, the major $\mathbf{L}^2$ and the minor $\mathbf{L}^2$ complex, respectively. Conversely, distance ratio between one proton on the C7/C12 and one proton on each of the C8/C11 and C9/C10 was 1.28, 1.22 and 1.16 for the minor $\mathbf{L}^2$, the major $\mathbf{L}^2$ and the $\mathbf{L}^1$ complex, respectively. Overall, the present results indicate that the *trans* configuration can be ruled out. The two complexes formed by $\mathbf{L}^2$ are very similar to the one formed by $\mathbf{L}^1$, with the latter being the more "compact", followed by the more abundant $\mathbf{L}^2$ complex and finally by the less abundant one, which are more "open" but still in a "book" conformation.

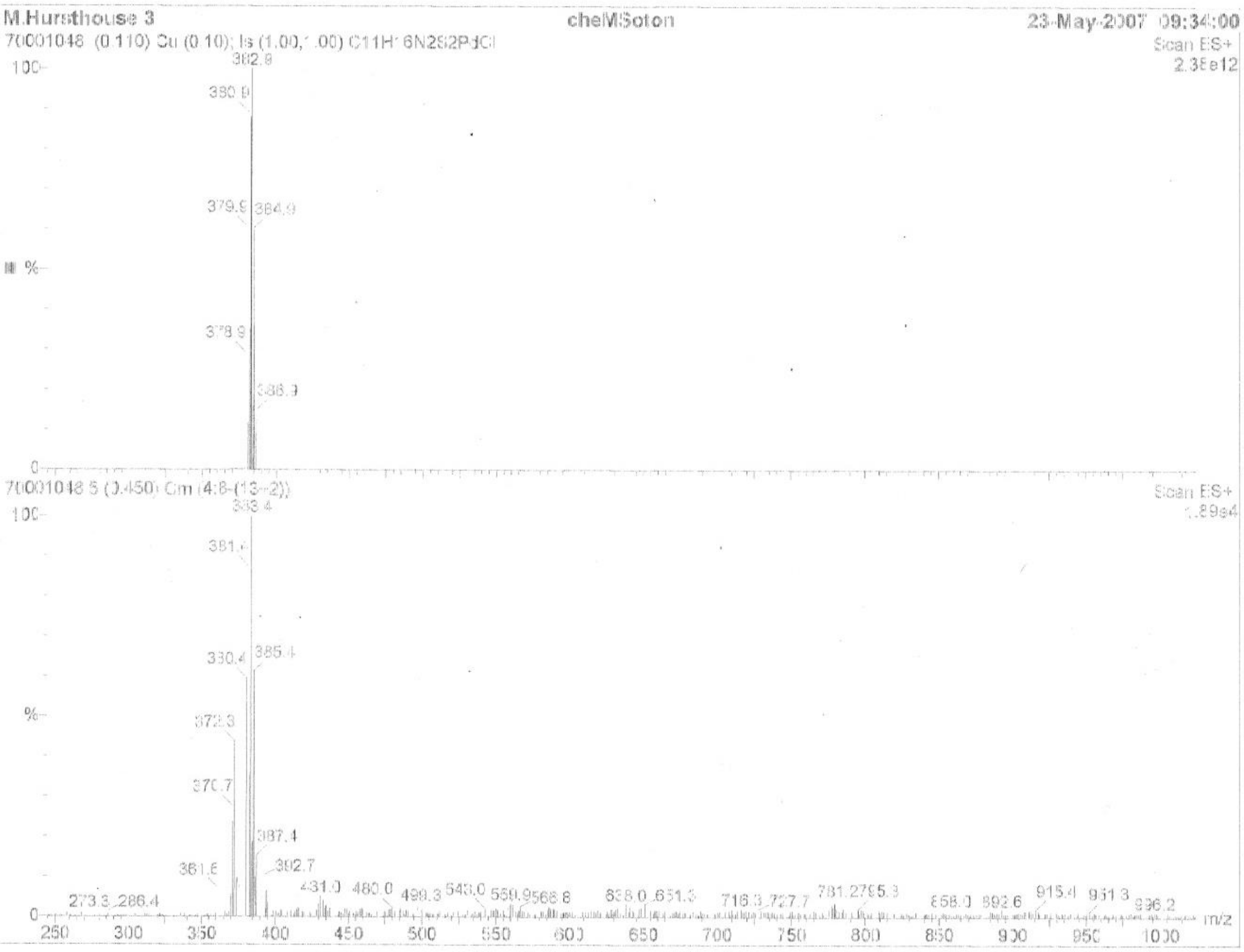

Figure S29. FAB Mass Spectrum of [Pd($\mathbf{L}^3$)Cl](PF_6).

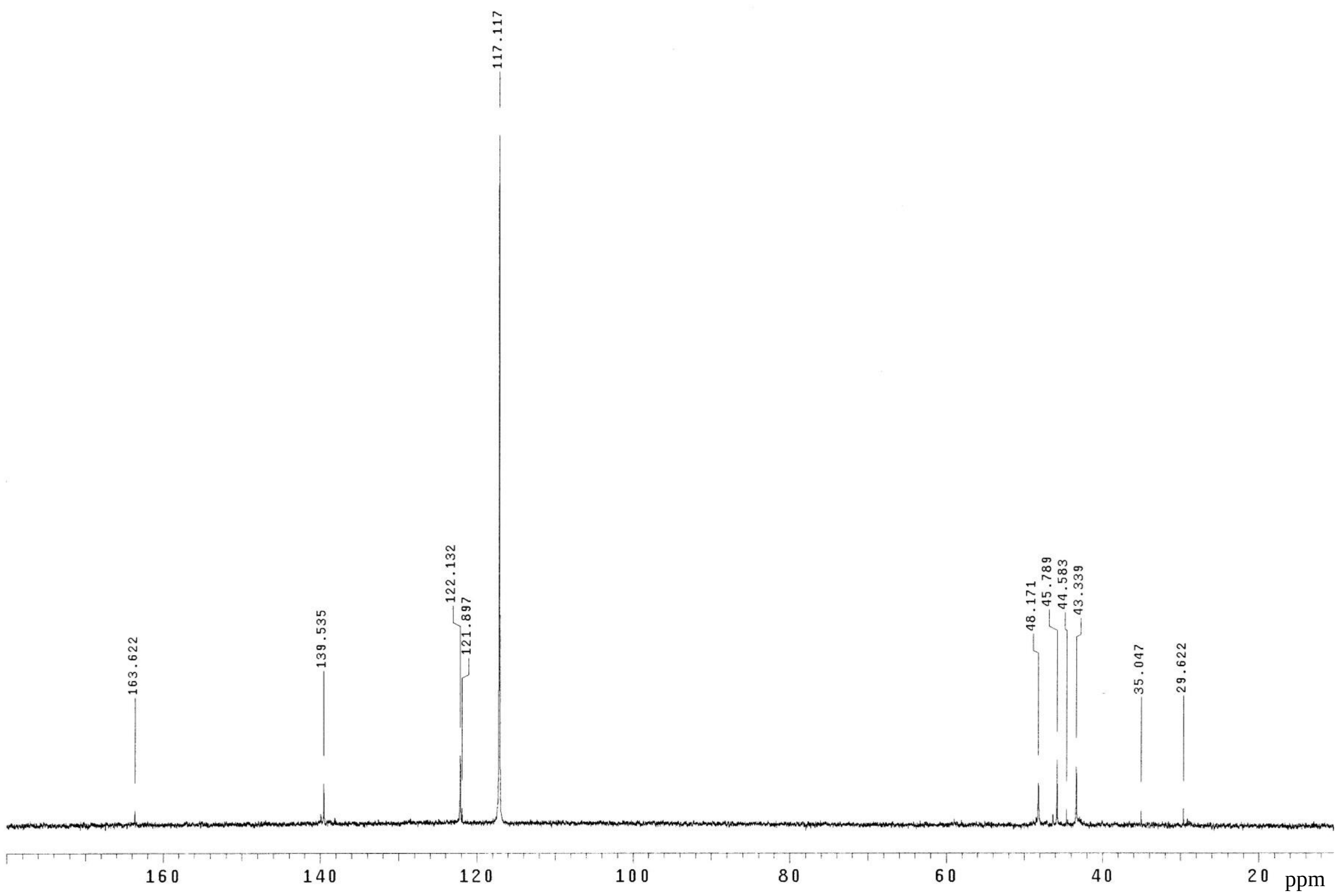

Figure S30. ^{13}C-NMR spectrum of $[Pd(\mathbf{L}^3)Cl](PF_6)$ recorded in CD_3CN (100.62 MHz).

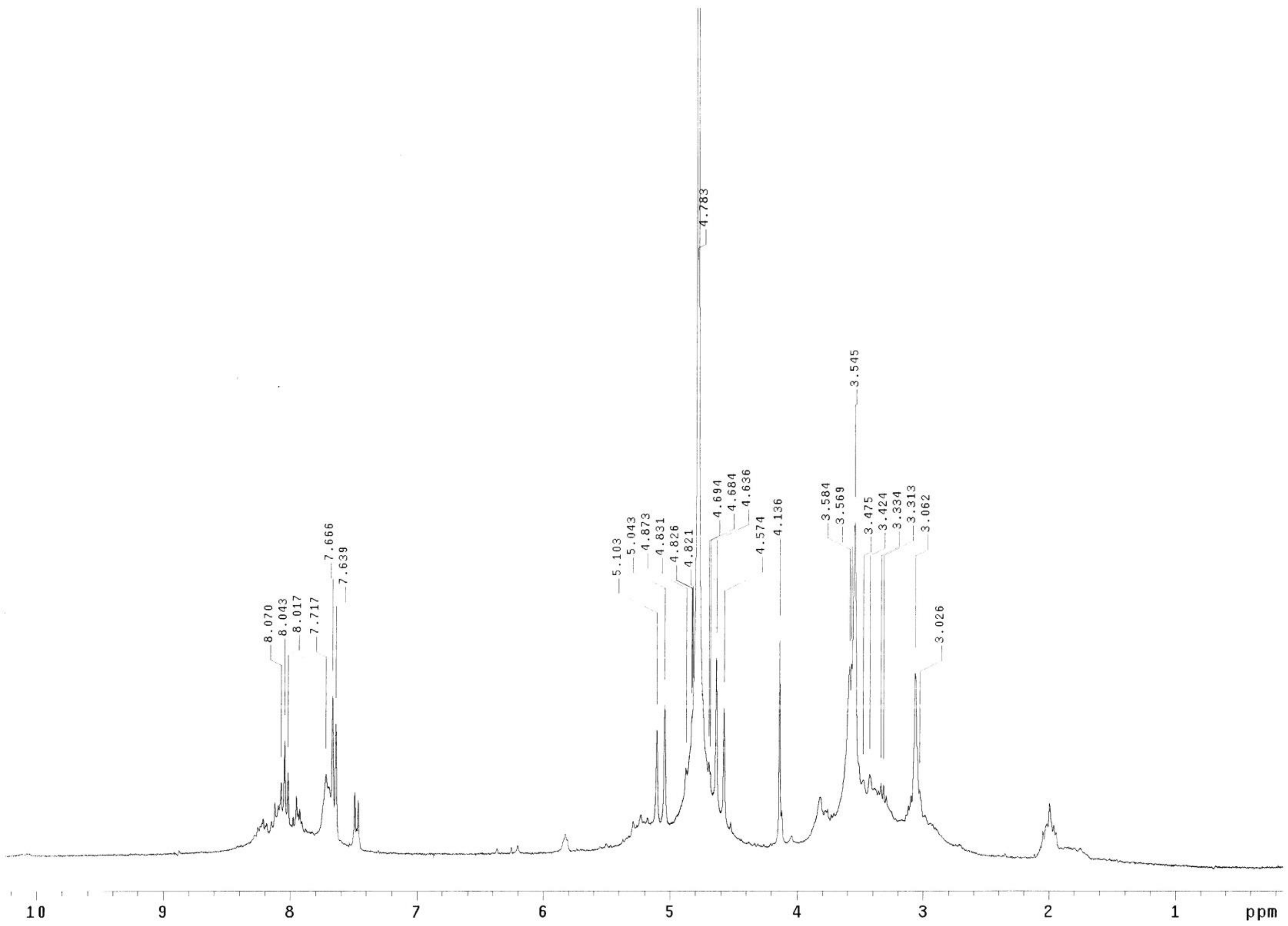

Figure S31. ^{1}H-NMR spectrum of [Pd(**L**^{3})Cl](PF_6) recorded in D_2O (400 MHz).

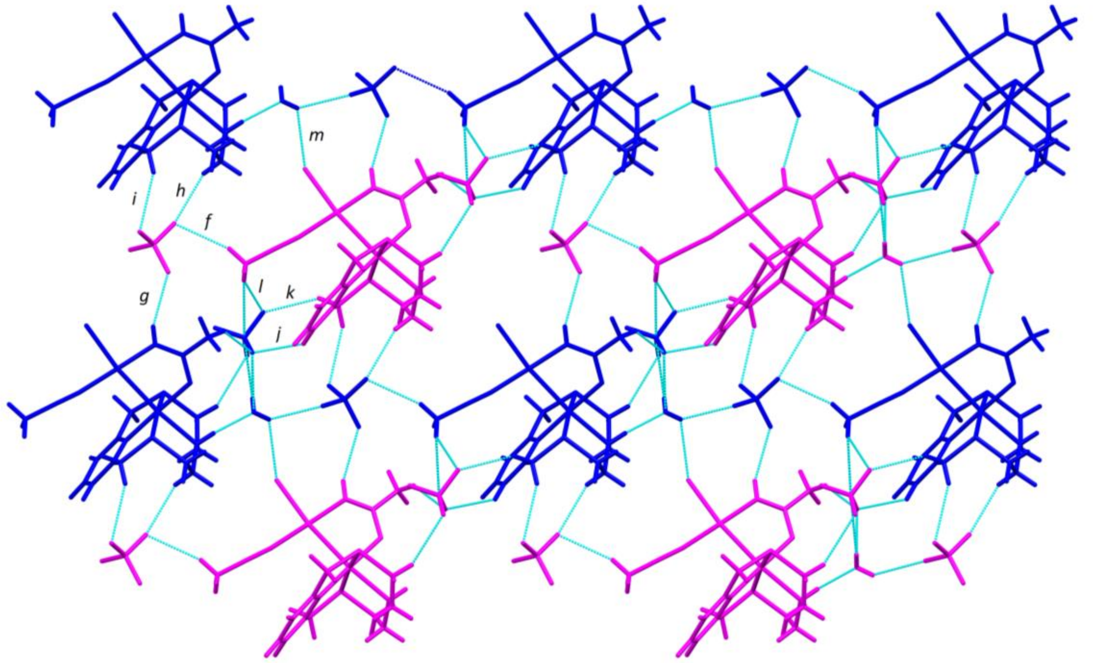

Figure S32. Packing view of symmetry-related interacting chains of complex cations, BF_4^- anions and H_2O molecules in [Pt(**L**^{3})(μ-1,3-MeCONH)PtCl(MeCN)]$(BF_4)_2 \cdot H_2O$ along the [010] direction. H-atoms not involved in H-interactions were omitted for clarity. Interactions shown (D–H···A: D–H Å, H···A Å, D···A Å, D–H···A°): *f*, C16–H16B···F4^{i} 0.96(5), 2.59(5), 3.52(7), 161(5); *g*, N4–HN4···F6^{ii} 0.79(5), 2.44(5), 3.156(5), 151(5); *h*, C9–H9B···F4^{iii} 0.97, 2.48, 3.362(5), 151; *i*, C7–H7A···F3^{iv} 0.97, 2.51, 3.130(5), 122; *j*, C5–H5···F8^{iii} 0.93, 2.38, 3.122(6), 137; *k*, C7–H7B···F9^{iii} 0.97, 2.47, 3.284(6), 141; *l*, C16–H16C···F9^{iii} 0.96, 2.40, 3.279(7), 153; *m*, OW–HW1···Cl1^{v} 0.86(7) Å, 2.77(8) Å, 3.356(6) Å, 127(6)°. Symmetry codes: i = x, y, –1+z; ii = –1/2+x, 1/2–y, –1/2+z; iii = 1/2+x, 1/2–y, –1/2+z; iv = –3/2–x, –1/2+y, 5/2–z; v = 1/2+x, 1/2–y, 1/2+z.

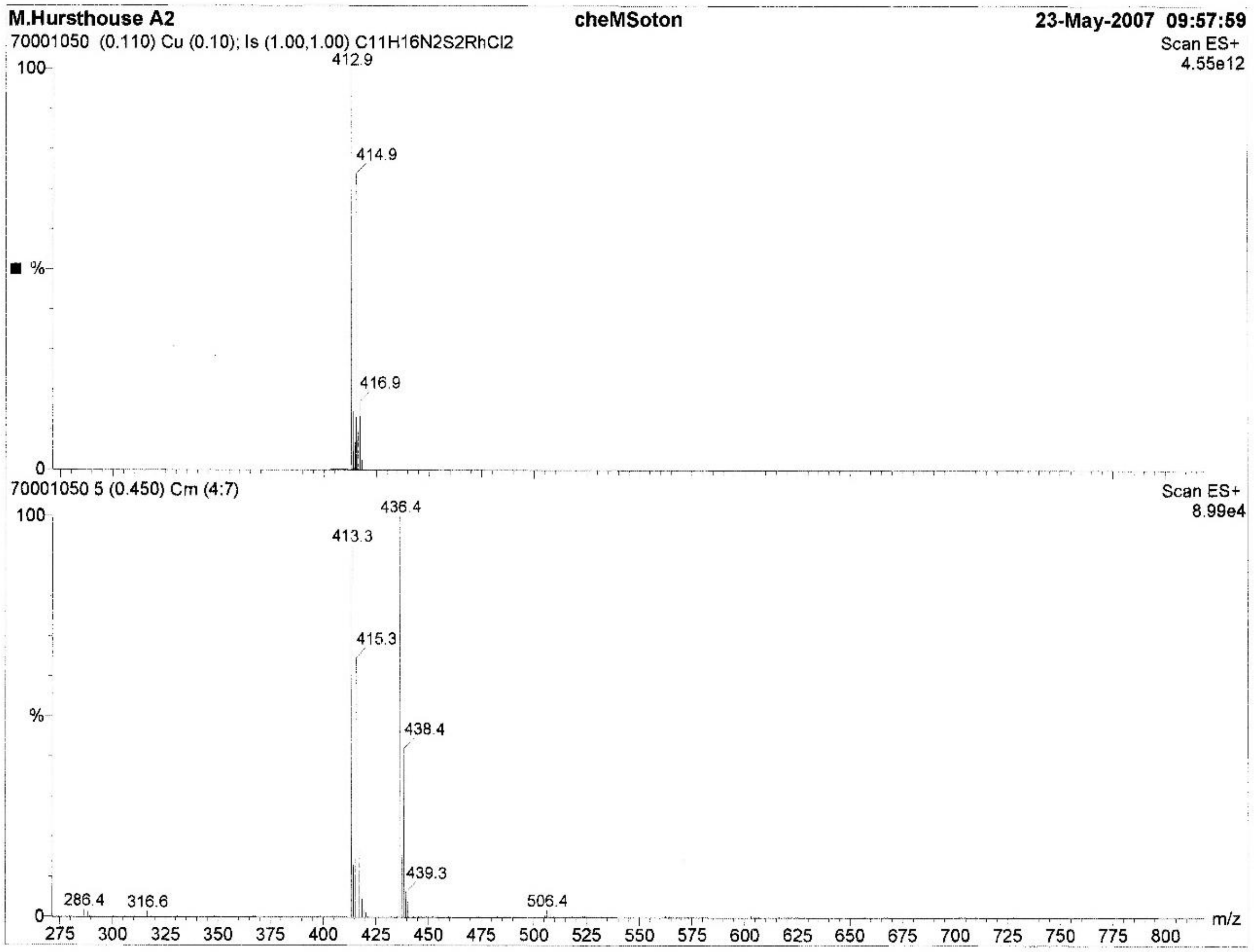

Figure S33. FAB Mass Spectrum of [Rh(**L**^{3})Cl_2](BF_4)·MeCN.

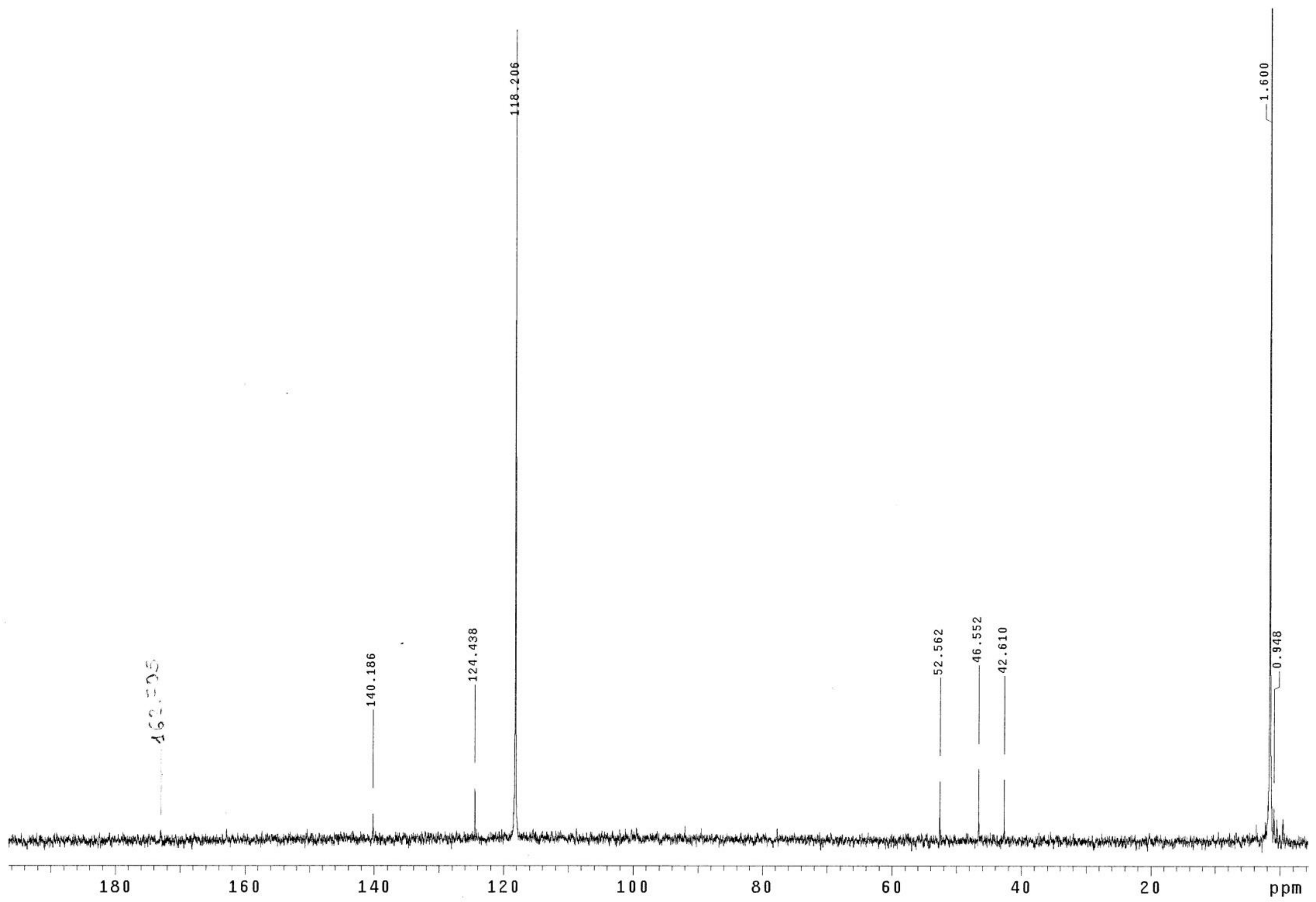

Figure S34. ^{13}C-NMR spectrum of [Rh(**L**^{3})Cl_2](BF_4)·MeCN recorded in CD_3CN (100.62 MHz).

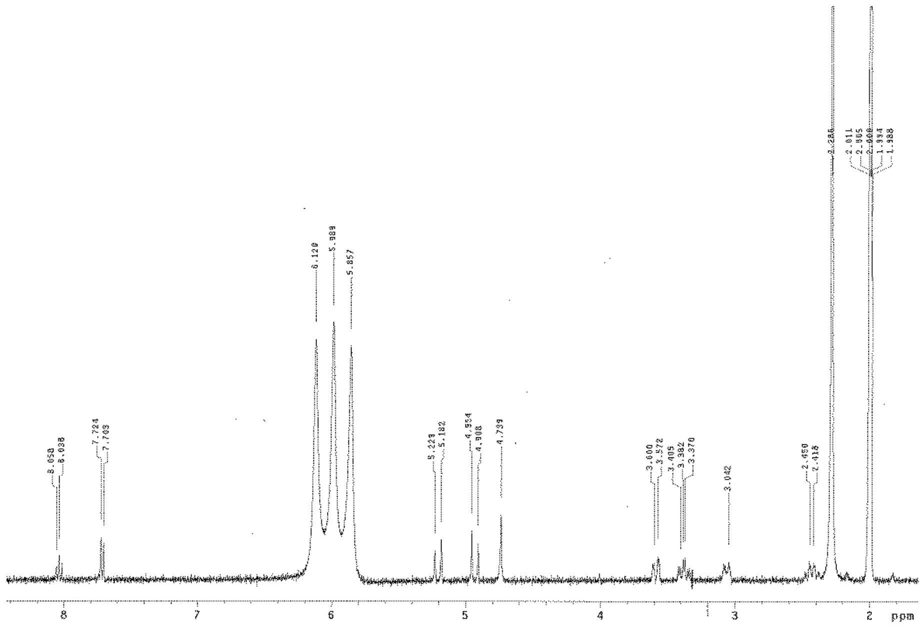

Figure S35. ^{1}H-NMR spectrum of [Rh($\mathbf{L}^3$)Cl_2](BF_4)·MeCN recorded in CD_3CN (400 MHz).

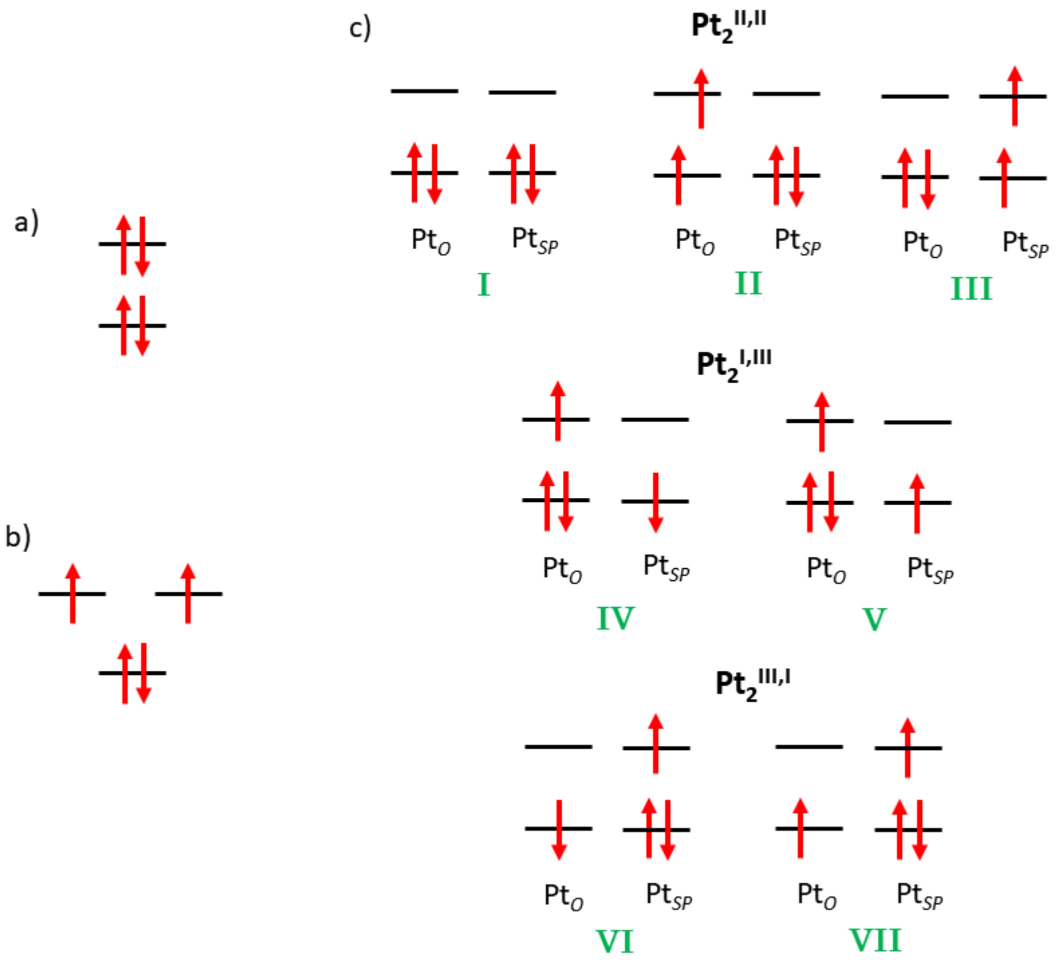

Figure S36. Schematic representation of the frontier molecular orbitals' electronic configuration for the closed-shell singlet state (a), triplet ground-state (b), and the different broken symmetry configurations (c) investigated at DFT level for $[Pt(\mathbf{L}^3)(\mu\text{-}1,3\text{-}MeCONH)PtCl(MeCN)]^{2+}$ and $[Pt_2(tfepma)_2Cl_4]$, featuring different charges (*Q* = +1, +2, +3) and spin multiplicities (2S + 1 = 1, 2, 3) on the octahedral (Pt_O) and square-planar (Pt_{SP}) Pt ions.

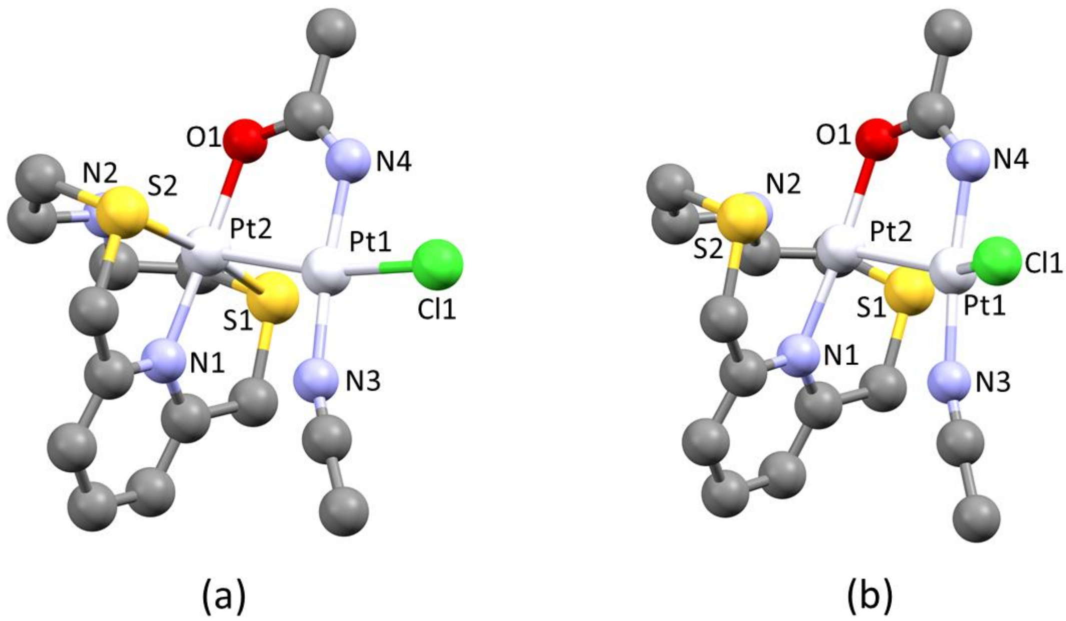

Figure S37. Molecular drawing and atom labelling scheme for the complex cation [Pt($\mathbf{L}^3$)(μ-1,3-MeCONH)PtCl(MeCN)]$^{2+}$ at the optimized geometry in its singlet (a) and triplet (b) state in the gas phase; hydrogen atoms were omitted for clarity.

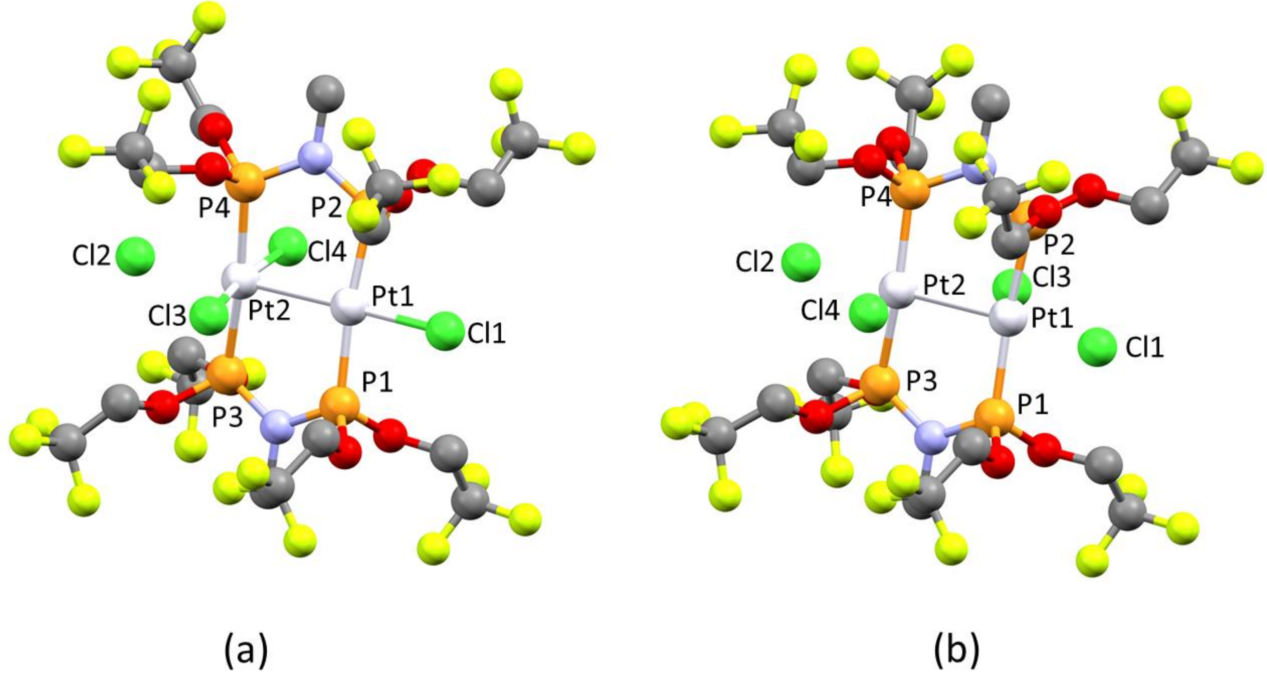

Figure S38. Molecular drawing and atom labelling scheme for [Pt_2(tfepma)$_2Cl_4$] at the optimized geometry in its singlet (a) and triplet (b) state in the gas phase; hydrogen atoms were omitted for clarity.

Table S1. Optimized geometry in orthogonal Cartesian coordinate format calculated for [Pt(**L**^{3})(μ-1,3-MeCONH)PtCl(MeCN)]$^{2+}$ in its singlet ground-state (total charge = +2, spin multiplicity = 1) at DFT level in the gas phase.

Atom number	Z	X	Y	Z
1	78	0.15426	-0.13609	-0.10323
2	78	1.91103	0.599047	1.706087
3	17	-1.6203	-0.90029	-1.34738
4	16	3.026343	-1.42381	1.662732
5	16	1.113347	2.764406	1.81966
6	8	0.612943	-0.04575	3.090312
7	7	-0.87376	-0.66508	1.498386
8	7	3.262359	1.266112	0.376894
9	7	3.258235	1.145354	3.54997
10	7	1.005042	0.316047	-1.8147
11	6	3.24966	2.569344	0.008907
12	6	4.266066	3.089337	-0.78595
13	1	4.249778	4.143107	-1.06624
14	6	5.290298	2.252173	-1.21817
15	1	6.100661	2.645408	-1.83436
16	6	5.263561	0.905633	-0.8677
17	1	6.040556	0.222773	-1.21301
18	6	4.227558	0.428528	-0.07125
19	6	4.095979	-1.03276	0.241356
20	1	5.069172	-1.52441	0.377306
21	1	3.592897	-1.53679	-0.60158
22	6	4.10242	-1.13256	3.135961
23	1	3.514849	-1.58594	3.949775
24	1	5.000328	-1.75495	3.010787
25	6	4.456112	0.316909	3.442355
26	1	5.054304	0.321372	4.370675
27	1	5.100155	0.737254	2.655556
28	6	3.41459	2.597203	3.527794
29	1	4.131659	2.857715	2.735024
30	1	3.836109	2.988411	4.470618
31	6	2.079932	3.295459	3.301827
32	1	2.19482	4.387411	3.240833
33	1	1.37571	3.097515	4.125216
34	6	2.06905	3.404592	0.407541
35	1	1.335037	3.405916	-0.41643
36	1	2.339252	4.451913	0.601016
37	6	-0.53498	-0.55681	2.740779
38	6	-1.40903	-0.99786	3.866156
39	1	-1.61975	-0.14415	4.524802
40	1	-0.88398	-1.75454	4.465145
41	1	-2.3548	-1.41662	3.506067
42	6	1.204683	0.449343	-2.94473
43	6	1.390587	0.587477	-4.37227
44	1	1.527789	1.642212	-4.64649
45	1	0.490293	0.205135	-4.87604
50	1	2.258334	0.004525	-4.70948
51	1	2.689975	0.85588	4.348386
52	1	-1.78689	-1.07481	1.305421

Table S2. Optimized geometry in orthogonal Cartesian coordinate format calculated for [Pt(**L**^{3})(μ-1,3-MeCONH)PtCl(MeCN)]$^{2+}$ in its triplet ground-state (total charge = +2, spin multiplicity = 3) at DFT level in the gas phase.

Atom number	Z	X	Y	Z
1	78	0.072895	-0.23203	-0.06548
2	78	1.822549	0.526777	1.649945
3	17	-1.89609	0.300763	-1.14683
4	16	3.262613	-1.62843	1.570325
5	16	1.022896	2.937261	1.9111
6	8	0.579843	-0.18834	3.08535
7	7	-0.98472	-0.67732	1.542937
8	7	3.186659	1.28297	0.334166
9	7	3.159877	1.043451	3.334451
10	7	1.027512	0.078505	-1.77309
11	6	3.147957	2.601802	0.029037
12	6	4.164269	3.187938	-0.72167
13	1	4.118972	4.253124	-0.95113
14	6	5.228234	2.409913	-1.16348
15	1	6.038121	2.855668	-1.74346
16	6	5.248246	1.057146	-0.84545
17	1	6.070333	0.419807	-1.17338
18	6	4.210108	0.508824	-0.09457
19	6	4.219768	-0.97684	0.175533
20	1	5.253372	-1.34363	0.245109
21	1	3.772285	-1.49277	-0.69082
22	6	4.241334	-1.19286	3.046723
23	1	3.692463	-1.68809	3.863051
24	1	5.219979	-1.68972	2.980004
25	6	4.425523	0.289259	3.30909
26	1	4.95962	0.410261	4.266803
27	1	5.065801	0.740601	2.53731
28	6	3.378293	2.498413	3.434745
29	1	4.078992	2.783753	2.636944
30	1	3.874479	2.744116	4.389028
31	6	2.100026	3.309792	3.335789
32	1	2.331049	4.384766	3.334843
33	1	1.437195	3.132945	4.197398
34	6	1.971237	3.445246	0.451042
35	1	1.211418	3.421358	-0.34914
36	1	2.271093	4.496457	0.56061
37	6	-0.60042	-0.60031	2.785727
38	6	-1.49078	-0.9839	3.918898
39	1	-1.64139	-0.11544	4.574901
40	1	-1.00156	-1.76582	4.515631
41	1	-2.46463	-1.34957	3.575935
42	6	1.296008	0.168942	-2.89215
43	6	1.587819	0.266962	-4.3041
44	1	2.389185	0.99553	-4.48617
45	1	0.679708	0.592192	-4.83278
50	1	1.891352	-0.7154	-4.69276
51	1	2.590933	0.727185	4.126365
52	1	-1.94819	-0.96099	1.38201

Table S3. Selected optimized bond lengths (Å) and angles (°) calculated in the gas phase for [Pt($\mathbf{L}^3$)(μ-1,3-MeCONH)PtCl(MeCN)]$^{2+}$ at the optimized geometry in its singlet and triplet state, and corresponding crystal structure data (atom labelling scheme as in Figures 7 and S37).

	Optimized values		Experimental values
	Singlet state	Triplet state	
Pt1-Pt2	2.627	2.565	2.580
Pt1-N3	1.964	1.981	1.972
Pt1-Cl1	2.298	2.309	2.343
Pt1-N4	1.975	1.976	1.981
Pt2-N1	2.009	2.041	2.006
Pt2-S1	2.310	2.593	2.293
Pt2-N2	2.348	2.212	2.237
Pt2-O1	2.004	2.029	2.018
Pt2-S2	2.310	2.553	2.307
N3-Pt1-N4	173.54	174.31	176.45
N3-Pt1-Cl1	86.52	88.34	89.14
N3-Pt1-Pt2	104.24	101.62	98.41
Cl1-Pt1-N4	87.02	88.67	89.37
Cl1-Pt1-Pt2	169.24	145.76	172.43
N4-Pt1-Pt2	82.21	83.53	83.13
Pt1-Pt2-N1	94.99	97.72	94.44
Pt1-Pt2-S1	93.73	96.45	94.19
Pt1-Pt2-N2	171.78	172.20	173.63
Pt1-Pt2-O1	87.28	87.20	86.86
Pt1-Pt2-S2	93.74	96.45	93.17
N1-Pt2-S1	87.34	85.24	87.28
N1-Pt2-N2	93.23	90.02	91.82
N1-Pt2-O1	177.73	175.07	177.97
N1-Pt2-S2	87.34	85.72	86.97
S1-Pt2-N2	86.64	83.21	87.27
S1-Pt2-O1	92.52	93.95	91.08
S1-Pt2-S2	171.18	164.10	170.77
N2-Pt2-O1	84.50	85.06	86.92
N2-Pt2-S2	86.64	83.21	86.01
O1-Pt2-S2	92.52	93.95	94.87
Cl1-Pt1-Pt2-N2	0.06	89.51	1.99

Table S4. Optimized geometry in orthogonal Cartesian coordinate format calculated for [Pt_2(tfepma)$_2$$Cl_4$] in its singlet state (total charge = 0, spin multiplicity = 1) at DFT level in the gas phase.

Atom number	Z	X	Y	Z
1	78	-0.20338	1.498997	0.007132
2	78	0.125678	-1.09874	-0.21499
3	17	-0.53461	3.831899	0.398239
4	17	0.457791	-3.58112	-0.35758
5	17	-0.03361	-0.77024	-2.54063
6	17	0.201757	-1.05525	2.152832
7	15	-2.42728	1.352723	-0.3607
8	15	2.013255	1.88053	0.288263
9	15	-2.13774	-1.513	-0.17825
10	15	2.417197	-0.92278	-0.32338
11	8	-3.36362	2.090889	0.744474
12	8	-3.02888	2.038421	-1.68522
13	8	2.714795	2.927971	-0.71443
14	8	2.443475	2.568483	1.698655
15	8	-2.75946	-2.00257	1.221045
16	8	-2.71869	-2.67642	-1.11902
17	8	3.074306	-1.13942	-1.76942
18	8	3.306539	-1.9687	0.510281
19	6	-3.06013	2.029106	2.12238
20	1	-2.31694	2.795893	2.382363
21	1	-2.68365	1.038783	2.422219
22	6	-4.33855	2.296767	2.885593
23	6	-3.16245	3.431885	-1.87398
24	1	-2.18307	3.912992	-1.99991
25	1	-3.68673	3.896181	-1.02781
26	6	-3.98729	3.635761	-3.12532
27	6	2.639746	4.334693	-0.63627
28	1	2.664226	4.679303	0.405698
29	1	1.725129	4.704533	-1.11733
30	6	3.847602	4.895512	-1.3546
31	6	1.804657	2.252333	2.917053
32	1	1.029393	1.480581	2.792072
33	1	1.348181	3.160661	3.331707
34	6	2.834317	1.731652	3.897103
35	6	-2.48647	-3.27137	1.788943
36	1	-1.42604	-3.35964	2.060688
37	1	-2.75845	-4.07644	1.092804
38	6	-3.33492	-3.39241	3.034337
39	6	-2.2878	-3.14187	-2.38279
40	1	-1.26288	-3.53037	-2.31854
41	1	-2.33847	-2.35093	-3.14151
42	6	-3.23357	-4.25658	-2.76906
43	6	3.165156	-2.39391	-2.41731
44	1	2.206977	-2.655	-2.88547
45	1	3.461559	-3.18303	-1.71353
50	6	4.231172	-2.27059	-3.483
51	6	3.038677	-2.63864	1.726221
52	1	2.13179	-3.25078	1.635814
53	1	2.932341	-1.93132	2.557803
54	6	4.228534	-3.53221	1.994258
55	6	-4.56391	-0.33295	-0.65039
56	1	-4.77371	-1.30687	-1.1062
57	1	-4.93064	0.454456	-1.31834

58	1	-5.06483	-0.26165	0.32297
59	6	4.515465	0.735921	0.175543
60	1	4.887155	0.358244	1.135271
61	1	4.760705	1.797774	0.070844
62	1	4.988062	0.190014	-0.64961
63	9	-5.26412	1.370924	2.628862
64	9	-4.8536	3.484032	2.578434
65	9	-4.08125	2.276465	4.192612
66	9	-4.16798	4.942179	-3.31891
67	9	-5.18622	3.061092	-3.01517
68	9	-3.39048	3.13226	-4.20084
69	9	3.818801	6.226277	-1.27905
70	9	4.985002	4.473119	-0.79608
71	9	3.87035	4.549645	-2.63695
72	9	3.426025	0.621424	3.435216
73	9	3.790265	2.623639	4.135706
74	9	2.239699	1.43376	5.049542
75	9	-3.05362	-2.43715	3.917764
76	9	-4.63441	-3.31459	2.751007
77	9	-3.10254	-4.57339	3.606766
78	9	-3.21779	-5.24833	-1.88088
79	9	-4.49162	-3.81816	-2.86432
80	9	-2.87443	-4.74661	-3.95354
81	9	3.918362	-1.35834	-4.39752
82	9	4.372224	-3.44629	-4.09557
83	9	5.408924	-1.93222	-2.95467
84	9	4.021493	-4.20883	3.122914

Table S5. Optimized geometry in orthogonal Cartesian coordinate format calculated for [Pt_2(tfepma)$_2Cl_4$] in its triplet state (total charge = 0, spin multiplicity = 3) at DFT level in the gas phase.

Atom number	Z	X	Y	Z
1	78	-0.33478	1.433065	0.074816
2	78	0.2378	-1.11515	-0.2275
3	17	-0.86437	3.634582	0.929841
4	17	0.789283	-3.4206	-0.78217
5	17	-0.08861	0.96222	-2.39629
6	17	0.101324	-1.20885	2.304749
7	15	-2.58939	1.072686	-0.02708
8	15	1.86924	2.002657	0.322727
9	15	-1.96694	-1.71054	-0.40235
10	15	2.496071	-0.73186	-0.3485
11	8	-3.40961	1.483671	1.310518
12	8	-3.4178	1.928205	-1.10251
13	8	2.419498	3.191181	-0.60637
14	8	2.253396	2.622697	1.774973
15	8	-2.60053	-2.63412	0.744477
16	8	-2.31966	-2.61061	-1.69816
17	8	3.155028	-0.93397	-1.80441
18	8	3.485711	-1.66401	0.519317
19	6	-2.93417	1.175199	2.60701
20	1	-2.49219	2.073368	3.058828
21	1	-2.18259	0.369068	2.601439
22	6	-4.10904	0.725473	3.447893
23	6	-3.70883	3.306346	-0.99767
24	1	-2.82089	3.915018	-1.2136
25	1	-4.08615	3.556648	0.002724
26	6	-4.78744	3.614302	-2.01303
27	6	2.121661	4.562487	-0.45222
28	1	2.162824	4.862558	0.603063
29	1	1.128553	4.797204	-0.85705
30	6	3.171364	5.339935	-1.21612
31	6	1.72857	2.114017	2.984027
32	1	1.074813	1.240466	2.826517
33	1	1.15954	2.905289	3.488948
34	6	2.87158	1.682712	3.877362
35	6	-2.04325	-3.85174	1.201617
36	1	-1.0981	-3.66646	1.729796
37	1	-1.87665	-4.55278	0.373018
38	6	-3.03641	-4.46091	2.166123
39	6	-1.7611	-2.35985	-2.97474
40	1	-0.80961	-2.89919	-3.0773
41	1	-1.58911	-1.28717	-3.14903
42	6	-2.73672	-2.86927	-4.01161
43	6	3.58663	-2.16333	-2.348
44	1	2.753616	-2.69006	-2.83127
45	1	4.024125	-2.81003	-1.5762
50	6	4.649567	-1.8569	-3.38007
51	6	3.171461	-2.47244	1.636538
52	1	2.278089	-3.08099	1.445267
53	1	3.016876	-1.86285	2.535216
54	6	4.354755	-3.38688	1.859964
55	6	-4.53786	-0.77889	-0.57782
56	1	-4.6593	-1.5152	-1.38019
57	1	-5.06377	0.137389	-0.86592

58	1	-4.94677	-1.17027	0.361058
59	6	4.460031	1.135116	0.017559
60	1	4.90738	0.812762	0.965269
61	1	4.594393	2.213738	-0.11406
62	1	4.944962	0.62149	-0.82054
63	9	-4.669	-0.3791	2.952505
64	9	-5.05129	1.662971	3.517407
65	9	-3.68839	0.462919	4.683789
66	9	-5.11477	4.903126	-1.92023
67	9	-5.88469	2.888608	-1.7897
68	9	-4.38393	3.373084	-3.25548
69	9	2.942084	6.644168	-1.06193
70	9	4.397598	5.076415	-0.75886
71	9	3.150999	5.061664	-2.51435
72	9	3.577435	0.691185	3.313634
73	9	3.713742	2.679359	4.128338
74	9	2.388301	1.23788	5.033923
75	9	-3.26664	-3.66408	3.205691
76	9	-4.20422	-4.71067	1.575954
77	9	-2.54465	-5.61366	2.622178
78	9	-2.96193	-4.1733	-3.88119
79	9	-3.9127	-2.24214	-3.92429
80	9	-2.23827	-2.64931	-5.22701
81	9	4.180207	-1.10754	-4.37179
82	9	5.098068	-3.00138	-3.89564
83	9	5.685132	-1.21292	-2.83534
84	9	4.099463	-4.19108	2.889938

Table S6. Selected optimized bond lengths (Å) and angles (°) calculated in the gas phase for [$Pt_2(tfepma)_2Cl_4$] at the optimized geometry in its singlet and triplet state, and corresponding crystal structure data (atom labelling scheme as in Figure S38).

	Optimized values		Experimental values[a]
	Singlet state	Triplet state	
Pt1-Pt2	2.628	2.629	2.619
Pt1-Cl1	2.388	2.420	2.354
Pt1-P1	2.267	2.290	2.252
Pt1-P2	2.259	2.285	2.250
Pt1-Cl3	/	2.528	/
Pt2-Cl2	2.509	2.434	2.500
Pt2-Cl3	2.354	3.021	2.323
Pt2-Cl4	2.369	2.538	2.338
Pt2-P3	2.301	2.294	2.294
Pt2-P4	2.301	2.290	2.295
Cl1-Pt1-P1	87.19	86.91	87.04
Cl1-Pt1-Pt2	175.34	165.89	173.39
Cl1-Pt1-P2	87.33	86.75	87.97
Cl1-Pt1-Cl3	/	122.42	/
P1-Pt1-Pt2	93.12	92.52	92.97
P1-Pt1-P2	173.65	173.48	172.92
P1-Pt1-Cl3	/	93.35	
P2-Pt1-Pt2	92.61	93.26	92.57
P2-Pt1-Cl3	/	91.32	/
Cl2-Pt2-Cl3	95.21	/	93.66
Cl2-Pt2-Cl4	94.06	101.80	96.60
Cl2-Pt2-Pt1	178.36	173.39	177.95
Cl2-Pt2-P3	86.63	85.60	87.02
Cl2-Pt2-P4	87.30	87.39	88.30
Cl3-Pt2-Cl4	170.69	/	169.73
Cl3-Pt2-Pt1	86.40	71.69	87.94
Cl3-Pt2-P3	90.58	/	89.07
Cl3-Pt2-P4	88.53	/	92.14
Cl4-Pt2-Pt1	84.34	84.80	81.80
Cl4-Pt2-P3	90.78	96.42	91.12
Cl4-Pt2-P4	91.08	90.85	88.51
P3-Pt2-Pt1	93.04	93.35	91.72
P3-Pt2-P4	173.76	170.79	175.24
P4-Pt2-Pt1	93.06	92.93	92.92
Cl1-Pt1-Pt2-Cl2	24.67	176.62	27.08

[a] Taken from Refs. 46, 47 in the main text of the paper.

Table S7. Details of X-ray data collection and refinement for [Pd(**L**[1])Cl]$_2$[Pd_2Cl_6], [Pt(**L**[1])Cl](BF_4) and [Rh(**L**[1])Cl_2](PF_6).

**Complex**	[Pd(**L**[1])Cl]$_2$[Pd_2Cl_6]	[Pt(**L**[1])Cl](BF_4)	[Rh(**L**[1])Cl_2](PF_6)
Empirical formula	$C_{11}H_{15}Cl_4NOPd_2S_2$	$C_{11}H_{15}BClF_4NOPtS_2$	$C_{11}H_{15}Cl_2F_6NOPRhS_2$
Formula weight	595.96	558.71	560.14
Temperature/K	294(2)	150(2)	294(2)
Crystal system	Monoclinic	Triclinic	Monoclinic
Space group	$P2_1/n$	P-1	$P2_1/c$
a/Å	10.9866(6)	9.0592(14)	9.9357(10)
b/Å	12.5513(6)	10.087(2)	12.309(3)
c/Å	13.8633(7)	10.111(2)	14.771(3)
α/°	90	69.856(2)	90
β/°	110.060(10)	69.238(2)	92.550(10)
γ/°	90	74.787(2)	90
Volume/Å^3	1795.72(19)	800.7(4)	1804.8(6)
Z	4	2	4
ρ_{calc}g/cm^3	2.204	2.317	2.062
μ/mm^{-1}	2.825	9.228	1.622
F(000)	1152	528	1104
Crystal size/mm^3	0.14 × 0.09 × 0.04	0.84 × 0.26 × 0.06	0.21 × 0.10 × 0.09
Radiation	MoKα (λ = 0.71073)	MoKα (λ = 0.71073)	MoKα (λ = 0.71073)
2Θ range for data collection/°	4.110 to 58.566	4.36 to 52.74	6.036 to 49.978
Reflections collected	21966	6703	3166
Unique reflections/ R_{int}	4527/0.0225	3224/0.031	3166/0.0000
Observed reflections [I>=2σ (I)]	3485	3118	2319
Data/restraints/parameters	4527/0/190	3224/21/199	3166/0/226
Goodness-of-fit on F^2	1.033	1.05	1.050
Final R indexes [I > 2σ (I)]	R_1 = 0.0297, wR_2 = 0.0853	R_1 = 0.0302, wR_2 = 0.0802	R_1 = 0.0272, wR_2 = 0.0673
Final R indexes [all data]	R_1 = 0.0417, wR_2 = 0.0873	R_1 = 0.0313, wR_2 = 0.0812	R_1 = 0.0568, wR_2 = 0.0725
Largest diff. peak/hole / e Å^{-3}	0.818/-1.369	1.78/-1.36	0.496/-0.537

Table S8. Details of X-ray data collection and refinement for [Pt(**L**[3])(μ-1,3-MeCONH)PtCl(MeCN)]$(BF_4)_2\cdot H_2O$ and [Rh(**L**[3])Cl_2]$(BF_4)\cdot$MeCN.

**Complex**	[Pt(**L**[3])(μ-1,3-MeCONH)PtCl(MeCN)]$(BF_4)_2\cdot H_2O$	[Rh(**L**[3])Cl_2]$(BF_4)\cdot$MeCN
Empirical formula	$C_{15}H_{25}B_2ClF_8N_4O_2Pt_2S_2$	$C_{13}H_{19}BCl_2F_4NRhS_2$
Formula weight	956.76	542.05
Temperature/K	291(2)	150(2)
Crystal system	Monoclinic	Monoclinic
Space group	$P2_1/n$	$P2_1/n$
a/Å	13.3760(11)	7.0301(7)
b/Å	11.0568(9)	29.579(3)
c/Å	17.6337(14)	9.2899(9)
α/°	90	90
β/°	95.170(10)	90.476(2)
γ/°	90	90
Volume/Å^3	2597.3(4)	1931.7(6)
Z	4	4
ρ_{calc}g/cm^3	2.447	1.864
μ/mm^{-1}	11.103	1.417
F(000)	1784	1080
Crystal size/mm^3	0.20 × 0.07 × 0.05	0.48 × 0.17 × 0.06
Radiation	MoKα (λ = 0.71073)	MoKα (λ = 0.71073)
2Θ range for data collection/°	4.00 to 56.04	4.60 to 55.60
Reflections collected	26897	16408
Independent reflections/ R_{int}	6137/ 0.0379	4528/0.032
Observed reflections [I>=2σ (I)]	4831	4148
Data/restraints/parameters	6137/3/344	4421/0/237
Goodness-of-fit on F^2	0.692	1.15
Final R indexes [I>=2σ (I)]	R_1 = 0.0209, wR_2 = 0.0403	R_1 = 0.0539, wR_2 = 0.136
Final R indexes [all data]	R_1 = 0.0285, wR_2 = 0.0410	R_1 = 0.0568, wR_2 = 0.138
Largest diff. peak/hole / e Å^{-3}	1.482/-0.976	1.67/-2.28
